# Supplementary material for: Is the extreme within-population genome size variation real in Spodoptera frugiperda?
Source: PLoS One. 2025 Sep 30;20(9):e0332711. doi: 10.1371/journal.pone.0332711 (PMC12483198; doi:10.1371/journal.pone.0332711)
Supplement: S1 Table — (DOCX) [file pone.0332711.s001.docx]

Table S1. Chromosomal locations on the NCBI reference genome assembly of BUSCO genes specifically duplicated in Gui et al.’s genome assembly

| Busco gene | chromosome |
| --- | --- |
| 9236at7088 | chr1 |
| 11597at7088 | chr1 |
| 12960at7088 | chr1 |
| 1208at7088 | chr1 |
| 6001at7088 | chr1 |
| 8685at7088 | chr1 |
| 4412at7088 | chr1 |
| 14870at7088 | chr1 |
| 7024at7088 | chr1 |
| 9695at7088 | chr1 |
| 12397at7088 | chr1 |
| 5380at7088 | chr1 |
| 1523at7088 | chr1 |
| 9506at7088 | chr2 |
| 10192at7088 | chr2 |
| 1958at7088 | chr2 |
| 12733at7088 | chr2 |
| 9364at7088 | chr2 |
| 2659at7088 | chr2 |
| 13607at7088 | chr2 |
| 2864at7088 | chr2 |
| 10565at7088 | chr2 |
| 4802at7088 | chr2 |
| 9286at7088 | chr2 |
| 14016at7088 | chr2 |
| 8596at7088 | chr2 |
| 2371at7088 | chr2 |
| 3599at7088 | chr3 |
| 13166at7088 | chr3 |
| 7887at7088 | chr3 |
| 4480at7088 | chr3 |
| 11046at7088 | chr3 |
| 9760at7088 | chr3 |
| 1693at7088 | chr3 |
| 7264at7088 | chr3 |
| 7154at7088 | chr3 |
| 5317at7088 | chr4 |
| 486at7088 | chr4 |
| 6983at7088 | chr4 |
| 4415at7088 | chr4 |
| 3186at7088 | chr4 |
| 12136at7088 | chr4 |
| 8550at7088 | chr4 |
| 6899at7088 | chr4 |
| 14849at7088 | chr4 |
| 15379at7088 | chr4 |
| 325at7088 | chr4 |
| 1660at7088 | chr5 |
| 5095at7088 | chr5 |
| 11633at7088 | chr5 |
| 5541at7088 | chr5 |
| 6555at7088 | chr5 |
| 15633at7088 | chr5 |
| 6186at7088 | chr5 |
| 3919at7088 | chr5 |
| 1911at7088 | chr5 |
| 8804at7088 | chr5 |
| 148at7088 | chr5 |
| 7789at7088 | chr5 |
| 13199at7088 | chr5 |
| 15237at7088 | chr5 |
| 13654at7088 | chr5 |
| 3898at7088 | chr5 |
| 1021at7088 | chr5 |
| 2090at7088 | chr6 |
| 14132at7088 | chr6 |
| 14147at7088 | chr6 |
| 4454at7088 | chr6 |
| 4664at7088 | chr6 |
| 3507at7088 | chr6 |
| 4253at7088 | chr6 |
| 139at7088 | chr6 |
| 3107at7088 | chr6 |
| 4760at7088 | chr6 |
| 6663at7088 | chr6 |
| 12061at7088 | chr6 |
| 4020at7088 | chr6 |
| 14762at7088 | chr6 |
| 7988at7088 | chr7 |
| 10999at7088 | chr7 |
| 4046at7088 | chr7 |
| 1130at7088 | chr7 |
| 3137at7088 | chr7 |
| 12730at7088 | chr7 |
| 2398at7088 | chr7 |
| 18at7088 | chr7 |
| 9106at7088 | chr7 |
| 9332at7088 | chr7 |
| 7837at7088 | chr7 |
| 11965at7088 | chr7 |
| 11616at7088 | chr7 |
| 4663at7088 | chr7 |
| 3528at7088 | chr7 |
| 7986at7088 | chr8 |
| 2452at7088 | chr8 |
| 6021at7088 | chr8 |
| 12006at7088 | chr8 |
| 5082at7088 | chr8 |
| 8585at7088 | chr8 |
| 4820at7088 | chr8 |
| 13230at7088 | chr8 |
| 11648at7088 | chr8 |
| 11237at7088 | chr8 |
| 11958at7088 | chr8 |
| 13714at7088 | chr8 |
| 14668at7088 | chr8 |
| 6582at7088 | chr8 |
| 14232at7088 | chr8 |
| 4241at7088 | chr8 |
| 9656at7088 | chr9 |
| 12704at7088 | chr9 |
| 3353at7088 | chr9 |
| 7817at7088 | chr9 |
| 861at7088 | chr9 |
| 9524at7088 | chr9 |
| 13088at7088 | chr9 |
| 12060at7088 | chr9 |
| 1711at7088 | chr9 |
| 6385at7088 | chr9 |
| 11857at7088 | chr9 |
| 8559at7088 | chr9 |
| 5290at7088 | chr9 |
| 6198at7088 | chr9 |
| 13095at7088 | chr9 |
| 7117at7088 | chr10 |
| 3363at7088 | chr10 |
| 7303at7088 | chr10 |
| 10379at7088 | chr10 |
| 14175at7088 | chr10 |
| 13463at7088 | chr10 |
| 3947at7088 | chr10 |
| 7476at7088 | chr10 |
| 10457at7088 | chr10 |
| 4942at7088 | chr10 |
| 4597at7088 | chr10 |
| 9052at7088 | chr10 |
| 16011at7088 | chr10 |
| 8955at7088 | chr10 |
| 611at7088 | chr10 |
| 437at7088 | chr10 |
| 14247at7088 | chr10 |
| 13112at7088 | chr10 |
| 14069at7088 | chr10 |
| 3358at7088 | chr10 |
| 10960at7088 | chr10 |
| 9576at7088 | chr10 |
| 2813at7088 | chr10 |
| 9386at7088 | chr11 |
| 15593at7088 | chr11 |
| 2762at7088 | chr11 |
| 10990at7088 | chr11 |
| 11969at7088 | chr11 |
| 7668at7088 | chr11 |
| 10029at7088 | chr11 |
| 14836at7088 | chr11 |
| 10913at7088 | chr11 |
| 10917at7088 | chr11 |
| 11973at7088 | chr11 |
| 3323at7088 | chr11 |
| 12396at7088 | chr11 |
| 7919at7088 | chr11 |
| 10164at7088 | chr11 |
| 5665at7088 | chr11 |
| 3351at7088 | chr11 |
| 7586at7088 | chr11 |
| 6302at7088 | chr11 |
| 7237at7088 | chr11 |
| 14127at7088 | chr11 |
| 3200at7088 | chr11 |
| 13034at7088 | chr11 |
| 11192at7088 | chr11 |
| 5094at7088 | chr12 |
| 9781at7088 | chr12 |
| 8885at7088 | chr12 |
| 5690at7088 | chr12 |
| 3899at7088 | chr12 |
| 3510at7088 | chr12 |
| 10244at7088 | chr12 |
| 3339at7088 | chr12 |
| 13059at7088 | chr12 |
| 929at7088 | chr12 |
| 11111at7088 | chr12 |
| 11921at7088 | chr12 |
| 15613at7088 | chr12 |
| 6585at7088 | chr13 |
| 4910at7088 | chr13 |
| 12691at7088 | chr13 |
| 13305at7088 | chr13 |
| 9603at7088 | chr13 |
| 10268at7088 | chr13 |
| 5767at7088 | chr13 |
| 13004at7088 | chr13 |
| 1351at7088 | chr13 |
| 6031at7088 | chr13 |
| 11072at7088 | chr13 |
| 4011at7088 | chr13 |
| 6540at7088 | chr13 |
| 13774at7088 | chr13 |
| 5710at7088 | chr13 |
| 9362at7088 | chr13 |
| 6023at7088 | chr13 |
| 15271at7088 | chr13 |
| 3220at7088 | chr13 |
| 8579at7088 | chr13 |
| 14998at7088 | chr13 |
| 2819at7088 | chr13 |
| 4214at7088 | chr13 |
| 2032at7088 | chr13 |
| 6674at7088 | chr13 |
| 7246at7088 | chr13 |
| 8464at7088 | chr13 |
| 8183at7088 | chr13 |
| 4114at7088 | chr13 |
| 2385at7088 | chr13 |
| 11932at7088 | chr14 |
| 13761at7088 | chr14 |
| 15704at7088 | chr14 |
| 3989at7088 | chr14 |
| 6996at7088 | chr14 |
| 11224at7088 | chr14 |
| 6503at7088 | chr14 |
| 2215at7088 | chr14 |
| 10789at7088 | chr14 |
| 3774at7088 | chr14 |
| 649at7088 | chr14 |
| 6076at7088 | chr14 |
| 12059at7088 | chr15 |
| 7380at7088 | chr15 |
| 2848at7088 | chr15 |
| 6320at7088 | chr15 |
| 1718at7088 | chr15 |
| 7966at7088 | chr15 |
| 10586at7088 | chr15 |
| 7307at7088 | chr15 |
| 9528at7088 | chr15 |
| 4010at7088 | chr15 |
| 11901at7088 | chr15 |
| 2378at7088 | chr15 |
| 13250at7088 | chr15 |
| 5520at7088 | chr15 |
| 16468at7088 | chr15 |
| 14497at7088 | chr15 |
| 12656at7088 | chr15 |
| 8427at7088 | chr15 |
| 11598at7088 | chr15 |
| 11581at7088 | chr15 |
| 13782at7088 | chr15 |
| 1065at7088 | chr16 |
| 6207at7088 | chr16 |
| 1992at7088 | chr16 |
| 1553at7088 | chr16 |
| 3443at7088 | chr17 |
| 12699at7088 | chr17 |
| 14664at7088 | chr17 |
| 5157at7088 | chr17 |
| 14495at7088 | chr18 |
| 11235at7088 | chr18 |
| 10110at7088 | chr18 |
| 1172at7088 | chr18 |
| 10082at7088 | chr18 |
| 15126at7088 | chr18 |
| 8443at7088 | chr18 |
| 12998at7088 | chr18 |
| 7656at7088 | chr18 |
| 7070at7088 | chr18 |
| 7902at7088 | chr18 |
| 9180at7088 | chr18 |
| 11672at7088 | chr18 |
| 16028at7088 | chr18 |
| 200at7088 | chr18 |
| 363at7088 | chr18 |
| 14755at7088 | chr18 |
| 14971at7088 | chr19 |
| 14119at7088 | chr19 |
| 3730at7088 | chr19 |
| 4764at7088 | chr19 |
| 8306at7088 | chr19 |
| 8103at7088 | chr19 |
| 13002at7088 | chr19 |
| 11150at7088 | chr19 |
| 10142at7088 | chr19 |
| 16168at7088 | chr19 |
| 10171at7088 | chr19 |
| 16938at7088 | chr19 |
| 6140at7088 | chr20 |
| 3925at7088 | chr20 |
| 8967at7088 | chr20 |
| 9628at7088 | chr20 |
| 10386at7088 | chr20 |
| 690at7088 | chr20 |
| 7133at7088 | chr20 |
| 5093at7088 | chr20 |
| 12390at7088 | chr20 |
| 242at7088 | chr20 |
| 8254at7088 | chr21 |
| 805at7088 | chr21 |
| 7290at7088 | chr21 |
| 10062at7088 | chr21 |
| 3608at7088 | chr21 |
| 713at7088 | chr21 |
| 5048at7088 | chr21 |
| 9021at7088 | chr21 |
| 9850at7088 | chr21 |
| 12070at7088 | chr21 |
| 10620at7088 | chr21 |
| 9331at7088 | chr21 |
| 6437at7088 | chr21 |
| 6063at7088 | chr22 |
| 5900at7088 | chr22 |
| 5266at7088 | chr22 |
| 5091at7088 | chr22 |
| 7045at7088 | chr22 |
| 1318at7088 | chr22 |
| 6342at7088 | chr22 |
| 1962at7088 | chr22 |
| 13346at7088 | chr22 |
| 5793at7088 | chr22 |
| 12354at7088 | chr22 |
| 10673at7088 | chr22 |
| 10374at7088 | chr22 |
| 3611at7088 | chr22 |
| 1000at7088 | chr22 |
| 11132at7088 | chr22 |
| 8718at7088 | chr22 |
| 9430at7088 | chr22 |
| 6179at7088 | chr22 |
| 9004at7088 | chr22 |
| 6022at7088 | chr22 |
| 4625at7088 | chr22 |
| 5162at7088 | chr22 |
| 4953at7088 | chr22 |
| 5863at7088 | chr22 |
| 15461at7088 | chr22 |
| 6744at7088 | chr22 |
| 9947at7088 | chr22 |
| 647at7088 | chr23 |
| 7106at7088 | chr23 |
| 10124at7088 | chr23 |
| 49at7088 | chr23 |
| 254at7088 | chr23 |
| 15278at7088 | chr24 |
| 3683at7088 | chr24 |
| 2672at7088 | chr24 |
| 9890at7088 | chr24 |
| 9517at7088 | chr24 |
| 4655at7088 | chr24 |
| 3818at7088 | chr24 |
| 4162at7088 | chr24 |
| 2483at7088 | chr24 |
| 12891at7088 | chr24 |
| 8222at7088 | chr24 |
| 5920at7088 | chr24 |
| 9121at7088 | chr24 |
| 8354at7088 | chr24 |
| 10066at7088 | chr24 |
| 6131at7088 | chr24 |
| 9556at7088 | chr24 |
| 9494at7088 | chr24 |
| 1626at7088 | chr24 |
| 7854at7088 | chr24 |
| 2339at7088 | chr24 |
| 13323at7088 | chr25 |
| 8571at7088 | chr25 |
| 1144at7088 | chr25 |
| 9956at7088 | chr26 |
| 8301at7088 | chr26 |
| 11043at7088 | chr26 |
| 5413at7088 | chr26 |
| 11283at7088 | chr26 |
| 8613at7088 | chr26 |
| 5258at7088 | chr26 |
| 7676at7088 | chr26 |
| 13310at7088 | chr26 |
| 13793at7088 | chr26 |
| 2774at7088 | chr26 |
| 5488at7088 | chr26 |
| 4037at7088 | chr27 |
| 2324at7088 | chr27 |
| 6652at7088 | chr27 |
| 13014at7088 | chr27 |
| 14138at7088 | chr27 |
| 6846at7088 | chr27 |
| 3888at7088 | chr27 |
| 5773at7088 | chr27 |
| 8849at7088 | chr27 |
| 1325at7088 | chr27 |
| 10243at7088 | chr27 |
| 1377at7088 | chr27 |
| 7258at7088 | chr27 |
| 701at7088 | chr27 |
| 14512at7088 | chr27 |
| 5937at7088 | chr27 |
| 12029at7088 | chr27 |
| 5500at7088 | chr27 |
| 14577at7088 | chr27 |
| 12831at7088 | chr28 |
| 10553at7088 | chr28 |
| 7277at7088 | chr28 |
| 6926at7088 | chr28 |
| 1583at7088 | chr28 |
| 12821at7088 | chr28 |
| 2796at7088 | chr28 |
| 4481at7088 | chr28 |
| 16103at7088 | chr28 |
| 7727at7088 | chr28 |
| 8479at7088 | chr28 |
| 7774at7088 | chr28 |
| 4880at7088 | chr28 |
| 4306at7088 | chr28 |
| 2629at7088 | chr28 |
| 11110at7088 | chr28 |
| 9360at7088 | chr29 |
| 1068at7088 | chr29 |
| 12896at7088 | chr29 |
| 8107at7088 | chr29 |
| 5625at7088 | chr29 |
| 12471at7088 | chr29 |
| 13483at7088 | chr29 |
| 8854at7088 | chr29 |
| 11011at7088 | chr29 |
| 2982at7088 | chr29 |
| 552at7088 | chr29 |
| 8861at7088 | chr30 |
| 1407at7088 | chr30 |
| 4350at7088 | chr30 |
| 8643at7088 | chr30 |
| 979at7088 | chr30 |
| 7125at7088 | chr30 |
| 6837at7088 | chr30 |
| 307at7088 | chr30 |
| 3793at7088 | chr30 |
| 4742at7088 | chr30 |
| 4790at7088 | chr30 |
| 14098at7088 | chr30 |
| 6622at7088 | chr30 |
| 7855at7088 | chr30 |
| 13043at7088 | chr30 |
| 10547at7088 | chr31 |
| 2560at7088 | chr31 |
| 13568at7088 | chr31 |
| 12082at7088 | chr31 |
| 7435at7088 | chr31 |
| 2163at7088 | chr31 |
| 2547at7088 | chr31 |
| 12503at7088 | chr31 |
| 6937at7088 | chr31 |
