# Supplementary material for: Is the extreme within-population genome size variation real in Spodoptera frugiperda?
Source: PLoS One. 2025 Sep 30;20(9):e0332711. doi: 10.1371/journal.pone.0332711 (PMC12483198; doi:10.1371/journal.pone.0332711)
Supplement: S2 Table — (DOCX) [file pone.0332711.s002.docx]

Table S2. The BUSCO genes found in the Reference genome assembly generated by Gui *et al.* or non-reference sequences generated by Huang *et al.*

| BUSCO Gene | Reference | non-Reference |
| --- | --- | --- |
| 0at7088 | Complete | Missing |
| 10001at7088 | Complete | Missing |
| 10003at7088 | Complete | Missing |
| 10006at7088 | Complete | Missing |
| 1000at7088 | Duplicated | Missing |
| 10013at7088 | Complete | Missing |
| 10014at7088 | Complete | Missing |
| 10015at7088 | Missing | Missing |
| 10017at7088 | Complete | Missing |
| 10020at7088 | Complete | Missing |
| 10023at7088 | Fragmented | Missing |
| 10024at7088 | Complete | Missing |
| 10025at7088 | Complete | Missing |
| 10029at7088 | Duplicated | Missing |
| 1003at7088 | Complete | Missing |
| 10043at7088 | Complete | Missing |
| 10048at7088 | Complete | Missing |
| 1004at7088 | Complete | Missing |
| 10056at7088 | Complete | Missing |
| 10057at7088 | Duplicated | Missing |
| 10059at7088 | Complete | Missing |
| 1005at7088 | Complete | Missing |
| 10062at7088 | Duplicated | Fragmented |
| 10066at7088 | Complete | Missing |
| 10067at7088 | Complete | Missing |
| 10068at7088 | Complete | Missing |
| 10076at7088 | Complete | Missing |
| 10079at7088 | Complete | Missing |
| 1007at7088 | Complete | Missing |
| 10082at7088 | Duplicated | Missing |
| 10086at7088 | Complete | Missing |
| 10087at7088 | Complete | Fragmented |
| 10090at7088 | Complete | Missing |
| 10093at7088 | Complete | Fragmented |
| 10096at7088 | Complete | Missing |
| 10097at7088 | Complete | Missing |
| 1009at7088 | Complete | Missing |
| 10101at7088 | Complete | Missing |
| 10102at7088 | Complete | Missing |
| 10107at7088 | Complete | Missing |
| 10109at7088 | Complete | Missing |
| 1010at7088 | Complete | Missing |
| 10110at7088 | Duplicated | Missing |
| 10114at7088 | Complete | Missing |
| 10118at7088 | Complete | Missing |
| 1011at7088 | Complete | Missing |
| 10121at7088 | Complete | Missing |
| 10124at7088 | Duplicated | Missing |
| 10125at7088 | Complete | Missing |
| 10127at7088 | Complete | Missing |
| 10139at7088 | Complete | Missing |
| 1013at7088 | Complete | Missing |
| 10142at7088 | Duplicated | Missing |
| 10145at7088 | Duplicated | Missing |
| 10146at7088 | Complete | Missing |
| 10149at7088 | Complete | Missing |
| 1014at7088 | Complete | Missing |
| 10150at7088 | Complete | Fragmented |
| 10151at7088 | Complete | Missing |
| 10153at7088 | Complete | Missing |
| 10157at7088 | Complete | Missing |
| 10160at7088 | Complete | Missing |
| 10163at7088 | Complete | Missing |
| 10164at7088 | Duplicated | Missing |
| 10171at7088 | Complete | Missing |
| 10173at7088 | Complete | Missing |
| 10174at7088 | Complete | Missing |
| 10178at7088 | Complete | Missing |
| 10179at7088 | Complete | Missing |
| 10180at7088 | Complete | Missing |
| 10181at7088 | Missing | Missing |
| 10183at7088 | Complete | Missing |
| 10186at7088 | Complete | Missing |
| 10188at7088 | Complete | Missing |
| 10189at7088 | Complete | Missing |
| 10192at7088 | Complete | Missing |
| 10193at7088 | Complete | Missing |
| 10199at7088 | Complete | Missing |
| 101at7088 | Complete | Missing |
| 10202at7088 | Missing | Missing |
| 10206at7088 | Complete | Missing |
| 10208at7088 | Complete | Complete |
| 1020at7088 | Complete | Missing |
| 10212at7088 | Complete | Missing |
| 10213at7088 | Complete | Missing |
| 10214at7088 | Duplicated | Complete |
| 10215at7088 | Complete | Missing |
| 10219at7088 | Complete | Missing |
| 1021at7088 | Duplicated | Missing |
| 10220at7088 | Complete | Missing |
| 10224at7088 | Complete | Missing |
| 10225at7088 | Complete | Missing |
| 10235at7088 | Complete | Missing |
| 10238at7088 | Complete | Missing |
| 10239at7088 | Complete | Missing |
| 10240at7088 | Complete | Missing |
| 10243at7088 | Duplicated | Missing |
| 10244at7088 | Complete | Missing |
| 10248at7088 | Missing | Missing |
| 10250at7088 | Complete | Missing |
| 10255at7088 | Complete | Missing |
| 10256at7088 | Complete | Missing |
| 10259at7088 | Complete | Missing |
| 10261at7088 | Complete | Missing |
| 10267at7088 | Complete | Missing |
| 10268at7088 | Duplicated | Missing |
| 10269at7088 | Complete | Missing |
| 1026at7088 | Complete | Missing |
| 10270at7088 | Missing | Missing |
| 1027at7088 | Complete | Missing |
| 10285at7088 | Complete | Missing |
| 10287at7088 | Complete | Missing |
| 10289at7088 | Complete | Complete |
| 10291at7088 | Missing | Missing |
| 10292at7088 | Complete | Missing |
| 10294at7088 | Complete | Missing |
| 10296at7088 | Complete | Missing |
| 10299at7088 | Complete | Missing |
| 10304at7088 | Complete | Missing |
| 10308at7088 | Missing | Missing |
| 10313at7088 | Complete | Missing |
| 10316at7088 | Complete | Missing |
| 10318at7088 | Missing | Missing |
| 10319at7088 | Complete | Missing |
| 10326at7088 | Missing | Missing |
| 1032at7088 | Complete | Missing |
| 10331at7088 | Complete | Missing |
| 10333at7088 | Complete | Missing |
| 10334at7088 | Complete | Missing |
| 10335at7088 | Complete | Missing |
| 1033at7088 | Complete | Missing |
| 10341at7088 | Complete | Missing |
| 10343at7088 | Complete | Missing |
| 10345at7088 | Complete | Missing |
| 10346at7088 | Complete | Missing |
| 10347at7088 | Complete | Complete |
| 10348at7088 | Fragmented | Missing |
| 10351at7088 | Complete | Missing |
| 10352at7088 | Complete | Missing |
| 10359at7088 | Complete | Complete |
| 10360at7088 | Complete | Missing |
| 10367at7088 | Missing | Missing |
| 10368at7088 | Fragmented | Missing |
| 10369at7088 | Complete | Missing |
| 10374at7088 | Complete | Missing |
| 10377at7088 | Missing | Fragmented |
| 10379at7088 | Duplicated | Missing |
| 1037at7088 | Complete | Missing |
| 10386at7088 | Duplicated | Missing |
| 10387at7088 | Complete | Missing |
| 1038at7088 | Complete | Missing |
| 10391at7088 | Complete | Missing |
| 10392at7088 | Complete | Missing |
| 10396at7088 | Complete | Missing |
| 10397at7088 | Complete | Fragmented |
| 10398at7088 | Complete | Missing |
| 103at7088 | Complete | Missing |
| 10401at7088 | Complete | Missing |
| 10406at7088 | Complete | Missing |
| 10410at7088 | Complete | Missing |
| 10414at7088 | Missing | Missing |
| 10415at7088 | Complete | Missing |
| 10418at7088 | Complete | Missing |
| 10423at7088 | Complete | Missing |
| 10424at7088 | Complete | Missing |
| 10425at7088 | Complete | Missing |
| 10428at7088 | Complete | Missing |
| 10429at7088 | Complete | Missing |
| 10431at7088 | Complete | Missing |
| 10432at7088 | Complete | Fragmented |
| 10437at7088 | Complete | Fragmented |
| 10444at7088 | Complete | Missing |
| 10445at7088 | Complete | Missing |
| 10447at7088 | Complete | Missing |
| 10452at7088 | Complete | Missing |
| 10456at7088 | Complete | Missing |
| 10457at7088 | Complete | Missing |
| 10459at7088 | Complete | Missing |
| 10460at7088 | Complete | Missing |
| 10462at7088 | Complete | Missing |
| 10474at7088 | Missing | Missing |
| 10476at7088 | Complete | Missing |
| 10479at7088 | Duplicated | Missing |
| 1047at7088 | Complete | Missing |
| 10480at7088 | Complete | Missing |
| 10482at7088 | Missing | Missing |
| 10489at7088 | Complete | Missing |
| 10494at7088 | Complete | Fragmented |
| 10496at7088 | Fragmented | Missing |
| 10502at7088 | Complete | Missing |
| 10506at7088 | Complete | Missing |
| 10508at7088 | Complete | Missing |
| 1050at7088 | Complete | Missing |
| 10511at7088 | Complete | Missing |
| 10512at7088 | Complete | Missing |
| 10513at7088 | Complete | Missing |
| 10516at7088 | Complete | Missing |
| 10517at7088 | Duplicated | Missing |
| 10521at7088 | Complete | Missing |
| 10527at7088 | Complete | Missing |
| 10528at7088 | Complete | Missing |
| 10529at7088 | Complete | Missing |
| 10530at7088 | Complete | Missing |
| 10533at7088 | Complete | Fragmented |
| 10536at7088 | Complete | Fragmented |
| 10538at7088 | Complete | Missing |
| 1053at7088 | Complete | Missing |
| 10541at7088 | Complete | Missing |
| 10542at7088 | Missing | Missing |
| 10543at7088 | Complete | Fragmented |
| 10546at7088 | Missing | Missing |
| 10547at7088 | Duplicated | Missing |
| 10549at7088 | Complete | Complete |
| 10550at7088 | Complete | Missing |
| 10553at7088 | Duplicated | Missing |
| 10556at7088 | Complete | Missing |
| 10558at7088 | Complete | Missing |
| 10559at7088 | Complete | Missing |
| 10562at7088 | Fragmented | Missing |
| 10565at7088 | Duplicated | Missing |
| 10568at7088 | Complete | Complete |
| 1056at7088 | Fragmented | Missing |
| 10570at7088 | Complete | Missing |
| 10571at7088 | Complete | Missing |
| 10573at7088 | Complete | Missing |
| 1057at7088 | Complete | Missing |
| 10580at7088 | Complete | Missing |
| 10581at7088 | Complete | Missing |
| 10582at7088 | Complete | Missing |
| 10583at7088 | Missing | Missing |
| 10586at7088 | Complete | Missing |
| 10588at7088 | Complete | Missing |
| 1058at7088 | Complete | Missing |
| 10593at7088 | Missing | Missing |
| 10598at7088 | Complete | Missing |
| 105at7088 | Complete | Missing |
| 10601at7088 | Complete | Duplicated |
| 10603at7088 | Complete | Missing |
| 10609at7088 | Complete | Missing |
| 10613at7088 | Complete | Missing |
| 10619at7088 | Complete | Missing |
| 10620at7088 | Duplicated | Missing |
| 10625at7088 | Complete | Missing |
| 10626at7088 | Complete | Missing |
| 10628at7088 | Complete | Missing |
| 10633at7088 | Complete | Complete |
| 10635at7088 | Complete | Missing |
| 10636at7088 | Complete | Missing |
| 1063at7088 | Missing | Missing |
| 10646at7088 | Complete | Missing |
| 10648at7088 | Missing | Missing |
| 10650at7088 | Complete | Missing |
| 10651at7088 | Complete | Missing |
| 10659at7088 | Complete | Missing |
| 1065at7088 | Complete | Missing |
| 10660at7088 | Complete | Missing |
| 10661at7088 | Complete | Missing |
| 10662at7088 | Fragmented | Missing |
| 10663at7088 | Missing | Missing |
| 10666at7088 | Complete | Missing |
| 10667at7088 | Complete | Missing |
| 10669at7088 | Complete | Missing |
| 1066at7088 | Missing | Missing |
| 10671at7088 | Complete | Missing |
| 10673at7088 | Complete | Missing |
| 10674at7088 | Complete | Missing |
| 10676at7088 | Complete | Missing |
| 10677at7088 | Complete | Missing |
| 10678at7088 | Missing | Missing |
| 10681at7088 | Complete | Missing |
| 10683at7088 | Complete | Complete |
| 10689at7088 | Complete | Missing |
| 1068at7088 | Duplicated | Missing |
| 10690at7088 | Complete | Missing |
| 10692at7088 | Complete | Missing |
| 10693at7088 | Complete | Missing |
| 10694at7088 | Complete | Missing |
| 10698at7088 | Complete | Missing |
| 10699at7088 | Complete | Complete |
| 106at7088 | Complete | Missing |
| 10704at7088 | Complete | Missing |
| 10707at7088 | Complete | Missing |
| 10709at7088 | Complete | Missing |
| 1070at7088 | Complete | Missing |
| 10712at7088 | Fragmented | Missing |
| 10713at7088 | Complete | Missing |
| 10716at7088 | Complete | Missing |
| 10717at7088 | Complete | Missing |
| 10721at7088 | Complete | Missing |
| 10724at7088 | Complete | Missing |
| 10728at7088 | Complete | Missing |
| 10734at7088 | Missing | Missing |
| 10735at7088 | Complete | Missing |
| 10738at7088 | Complete | Missing |
| 10740at7088 | Complete | Missing |
| 10747at7088 | Complete | Missing |
| 10749at7088 | Complete | Missing |
| 10750at7088 | Complete | Missing |
| 1075at7088 | Complete | Missing |
| 10766at7088 | Complete | Complete |
| 10768at7088 | Complete | Missing |
| 1076at7088 | Complete | Missing |
| 10773at7088 | Complete | Missing |
| 10778at7088 | Complete | Fragmented |
| 10780at7088 | Complete | Missing |
| 10782at7088 | Complete | Missing |
| 10783at7088 | Complete | Missing |
| 10784at7088 | Duplicated | Missing |
| 10789at7088 | Fragmented | Missing |
| 1078at7088 | Complete | Missing |
| 10791at7088 | Complete | Missing |
| 10792at7088 | Complete | Missing |
| 10793at7088 | Complete | Missing |
| 10796at7088 | Complete | Missing |
| 10799at7088 | Complete | Missing |
| 1079at7088 | Complete | Missing |
| 10801at7088 | Complete | Missing |
| 1080at7088 | Complete | Missing |
| 10810at7088 | Complete | Missing |
| 10812at7088 | Complete | Missing |
| 10814at7088 | Complete | Missing |
| 10816at7088 | Complete | Missing |
| 10818at7088 | Complete | Missing |
| 10821at7088 | Complete | Missing |
| 10822at7088 | Complete | Missing |
| 10826at7088 | Complete | Missing |
| 10827at7088 | Complete | Missing |
| 10829at7088 | Complete | Missing |
| 1082at7088 | Complete | Missing |
| 10830at7088 | Complete | Missing |
| 1083at7088 | Complete | Missing |
| 10841at7088 | Complete | Missing |
| 1084at7088 | Duplicated | Missing |
| 10853at7088 | Fragmented | Missing |
| 10858at7088 | Complete | Missing |
| 1085at7088 | Complete | Missing |
| 10862at7088 | Complete | Missing |
| 10865at7088 | Missing | Missing |
| 1086at7088 | Complete | Missing |
| 10875at7088 | Complete | Missing |
| 10878at7088 | Complete | Missing |
| 10879at7088 | Complete | Missing |
| 10887at7088 | Complete | Missing |
| 1088at7088 | Missing | Duplicated |
| 10896at7088 | Complete | Missing |
| 10898at7088 | Complete | Missing |
| 1089at7088 | Complete | Missing |
| 10904at7088 | Complete | Missing |
| 10906at7088 | Complete | Missing |
| 10908at7088 | Complete | Missing |
| 1090at7088 | Complete | Missing |
| 10913at7088 | Duplicated | Missing |
| 10915at7088 | Missing | Missing |
| 10916at7088 | Complete | Missing |
| 10917at7088 | Duplicated | Missing |
| 10926at7088 | Complete | Missing |
| 10929at7088 | Complete | Missing |
| 10930at7088 | Complete | Missing |
| 10933at7088 | Missing | Missing |
| 10936at7088 | Complete | Missing |
| 10939at7088 | Complete | Missing |
| 10940at7088 | Complete | Missing |
| 10943at7088 | Complete | Missing |
| 10947at7088 | Fragmented | Missing |
| 10948at7088 | Complete | Missing |
| 1094at7088 | Complete | Missing |
| 10952at7088 | Complete | Missing |
| 10953at7088 | Complete | Missing |
| 10959at7088 | Complete | Missing |
| 10960at7088 | Duplicated | Missing |
| 10963at7088 | Complete | Missing |
| 10965at7088 | Complete | Missing |
| 10966at7088 | Complete | Missing |
| 10973at7088 | Complete | Missing |
| 10976at7088 | Complete | Missing |
| 10977at7088 | Missing | Missing |
| 10981at7088 | Complete | Missing |
| 10982at7088 | Missing | Missing |
| 10983at7088 | Complete | Missing |
| 10988at7088 | Complete | Fragmented |
| 10990at7088 | Complete | Missing |
| 10997at7088 | Complete | Missing |
| 10999at7088 | Complete | Missing |
| 11004at7088 | Complete | Missing |
| 1100at7088 | Fragmented | Missing |
| 11010at7088 | Complete | Missing |
| 11011at7088 | Duplicated | Missing |
| 11014at7088 | Complete | Missing |
| 11018at7088 | Complete | Missing |
| 11020at7088 | Complete | Missing |
| 11022at7088 | Complete | Missing |
| 11023at7088 | Missing | Missing |
| 11026at7088 | Complete | Missing |
| 11029at7088 | Complete | Missing |
| 11030at7088 | Complete | Missing |
| 11032at7088 | Complete | Missing |
| 11033at7088 | Complete | Missing |
| 11034at7088 | Missing | Missing |
| 11039at7088 | Complete | Missing |
| 11041at7088 | Complete | Missing |
| 11043at7088 | Duplicated | Missing |
| 11046at7088 | Duplicated | Missing |
| 11053at7088 | Complete | Missing |
| 11054at7088 | Complete | Missing |
| 11056at7088 | Missing | Missing |
| 11058at7088 | Complete | Missing |
| 11059at7088 | Complete | Missing |
| 11060at7088 | Complete | Missing |
| 11062at7088 | Complete | Missing |
| 11063at7088 | Complete | Missing |
| 11065at7088 | Complete | Missing |
| 11066at7088 | Complete | Missing |
| 11067at7088 | Missing | Missing |
| 11069at7088 | Complete | Missing |
| 11070at7088 | Complete | Missing |
| 11072at7088 | Duplicated | Missing |
| 1107at7088 | Complete | Missing |
| 11080at7088 | Complete | Fragmented |
| 11086at7088 | Complete | Fragmented |
| 11089at7088 | Complete | Missing |
| 1108at7088 | Duplicated | Missing |
| 11090at7088 | Missing | Missing |
| 11095at7088 | Fragmented | Missing |
| 11100at7088 | Complete | Missing |
| 11101at7088 | Complete | Missing |
| 11106at7088 | Complete | Missing |
| 11110at7088 | Duplicated | Missing |
| 11111at7088 | Complete | Missing |
| 11115at7088 | Complete | Fragmented |
| 11119at7088 | Complete | Missing |
| 1111at7088 | Complete | Missing |
| 11122at7088 | Complete | Missing |
| 11124at7088 | Fragmented | Missing |
| 11127at7088 | Complete | Missing |
| 11132at7088 | Duplicated | Missing |
| 1113at7088 | Complete | Missing |
| 11142at7088 | Complete | Missing |
| 11144at7088 | Complete | Missing |
| 11147at7088 | Complete | Missing |
| 11148at7088 | Complete | Missing |
| 1114at7088 | Complete | Missing |
| 11150at7088 | Duplicated | Missing |
| 11151at7088 | Complete | Missing |
| 11158at7088 | Complete | Missing |
| 11160at7088 | Complete | Missing |
| 11161at7088 | Complete | Missing |
| 11165at7088 | Missing | Missing |
| 11166at7088 | Duplicated | Missing |
| 11174at7088 | Complete | Missing |
| 11175at7088 | Complete | Missing |
| 11177at7088 | Complete | Complete |
| 11178at7088 | Complete | Missing |
| 1117at7088 | Complete | Fragmented |
| 11184at7088 | Complete | Missing |
| 11185at7088 | Complete | Missing |
| 11189at7088 | Complete | Complete |
| 1118at7088 | Complete | Complete |
| 11190at7088 | Complete | Missing |
| 11191at7088 | Duplicated | Missing |
| 11192at7088 | Complete | Missing |
| 11195at7088 | Complete | Missing |
| 11198at7088 | Complete | Missing |
| 11201at7088 | Complete | Missing |
| 1120at7088 | Complete | Missing |
| 11213at7088 | Complete | Missing |
| 11214at7088 | Complete | Missing |
| 11215at7088 | Complete | Complete |
| 11218at7088 | Complete | Missing |
| 1121at7088 | Complete | Missing |
| 11221at7088 | Complete | Missing |
| 11224at7088 | Duplicated | Missing |
| 11225at7088 | Complete | Missing |
| 11231at7088 | Complete | Complete |
| 11233at7088 | Complete | Missing |
| 11234at7088 | Missing | Missing |
| 11235at7088 | Complete | Missing |
| 11237at7088 | Fragmented | Duplicated |
| 11239at7088 | Complete | Missing |
| 1123at7088 | Complete | Missing |
| 11240at7088 | Complete | Missing |
| 1124at7088 | Complete | Missing |
| 11256at7088 | Complete | Missing |
| 11258at7088 | Missing | Missing |
| 11260at7088 | Complete | Missing |
| 11262at7088 | Duplicated | Missing |
| 11264at7088 | Complete | Missing |
| 11265at7088 | Complete | Missing |
| 11268at7088 | Complete | Missing |
| 11269at7088 | Fragmented | Missing |
| 1126at7088 | Missing | Missing |
| 11272at7088 | Complete | Missing |
| 11273at7088 | Complete | Missing |
| 11274at7088 | Complete | Fragmented |
| 11275at7088 | Complete | Missing |
| 11282at7088 | Complete | Missing |
| 11283at7088 | Fragmented | Missing |
| 11285at7088 | Complete | Missing |
| 11288at7088 | Complete | Missing |
| 11292at7088 | Complete | Missing |
| 11293at7088 | Complete | Missing |
| 11294at7088 | Complete | Missing |
| 11295at7088 | Missing | Missing |
| 11298at7088 | Complete | Missing |
| 112at7088 | Complete | Missing |
| 11302at7088 | Complete | Missing |
| 11303at7088 | Complete | Missing |
| 1130at7088 | Duplicated | Missing |
| 11311at7088 | Complete | Missing |
| 11312at7088 | Duplicated | Missing |
| 11313at7088 | Complete | Missing |
| 11318at7088 | Complete | Missing |
| 1131at7088 | Complete | Missing |
| 11321at7088 | Complete | Missing |
| 11334at7088 | Complete | Missing |
| 11335at7088 | Complete | Missing |
| 11336at7088 | Complete | Fragmented |
| 11337at7088 | Fragmented | Missing |
| 11338at7088 | Complete | Missing |
| 1133at7088 | Complete | Missing |
| 11342at7088 | Complete | Missing |
| 11346at7088 | Complete | Missing |
| 11349at7088 | Complete | Missing |
| 1134at7088 | Complete | Missing |
| 11350at7088 | Complete | Missing |
| 11353at7088 | Complete | Missing |
| 11356at7088 | Complete | Missing |
| 11359at7088 | Complete | Missing |
| 1135at7088 | Complete | Missing |
| 11363at7088 | Missing | Duplicated |
| 11365at7088 | Complete | Missing |
| 11367at7088 | Missing | Missing |
| 11368at7088 | Complete | Missing |
| 1136at7088 | Complete | Missing |
| 11372at7088 | Complete | Missing |
| 11374at7088 | Complete | Missing |
| 11375at7088 | Complete | Missing |
| 11377at7088 | Complete | Missing |
| 11378at7088 | Complete | Missing |
| 11379at7088 | Complete | Missing |
| 11380at7088 | Complete | Missing |
| 11382at7088 | Fragmented | Missing |
| 11383at7088 | Complete | Missing |
| 11389at7088 | Complete | Missing |
| 11390at7088 | Complete | Fragmented |
| 11393at7088 | Fragmented | Missing |
| 11396at7088 | Complete | Missing |
| 11397at7088 | Complete | Missing |
| 1139at7088 | Complete | Missing |
| 113at7088 | Complete | Missing |
| 11401at7088 | Complete | Missing |
| 11403at7088 | Complete | Missing |
| 11404at7088 | Complete | Missing |
| 11405at7088 | Complete | Missing |
| 1140at7088 | Complete | Missing |
| 11416at7088 | Missing | Missing |
| 11418at7088 | Complete | Missing |
| 1141at7088 | Complete | Missing |
| 11423at7088 | Complete | Missing |
| 11426at7088 | Complete | Missing |
| 11428at7088 | Complete | Missing |
| 11437at7088 | Complete | Missing |
| 11438at7088 | Complete | Missing |
| 1143at7088 | Missing | Missing |
| 11441at7088 | Complete | Missing |
| 11442at7088 | Duplicated | Missing |
| 1144at7088 | Complete | Missing |
| 11450at7088 | Complete | Missing |
| 11451at7088 | Complete | Missing |
| 11454at7088 | Complete | Missing |
| 11456at7088 | Missing | Missing |
| 1145at7088 | Missing | Missing |
| 11462at7088 | Complete | Missing |
| 11474at7088 | Complete | Missing |
| 11479at7088 | Complete | Missing |
| 11482at7088 | Duplicated | Missing |
| 11483at7088 | Complete | Missing |
| 11484at7088 | Complete | Missing |
| 11489at7088 | Complete | Missing |
| 11490at7088 | Complete | Missing |
| 11492at7088 | Complete | Missing |
| 11493at7088 | Complete | Missing |
| 11500at7088 | Complete | Missing |
| 11501at7088 | Complete | Fragmented |
| 11505at7088 | Missing | Missing |
| 11507at7088 | Missing | Missing |
| 1150at7088 | Complete | Missing |
| 11511at7088 | Complete | Missing |
| 11512at7088 | Complete | Missing |
| 11516at7088 | Complete | Missing |
| 11518at7088 | Complete | Missing |
| 11519at7088 | Complete | Missing |
| 11522at7088 | Complete | Missing |
| 11525at7088 | Complete | Missing |
| 1152at7088 | Complete | Missing |
| 11533at7088 | Complete | Missing |
| 11534at7088 | Complete | Fragmented |
| 11536at7088 | Complete | Missing |
| 11537at7088 | Complete | Missing |
| 1153at7088 | Complete | Missing |
| 11546at7088 | Complete | Fragmented |
| 11547at7088 | Complete | Missing |
| 11548at7088 | Complete | Missing |
| 11549at7088 | Complete | Missing |
| 1154at7088 | Complete | Missing |
| 11550at7088 | Duplicated | Missing |
| 11558at7088 | Complete | Missing |
| 11560at7088 | Missing | Missing |
| 11563at7088 | Complete | Missing |
| 11571at7088 | Complete | Missing |
| 11575at7088 | Complete | Missing |
| 11576at7088 | Complete | Missing |
| 11580at7088 | Complete | Missing |
| 11581at7088 | Duplicated | Missing |
| 11582at7088 | Complete | Missing |
| 11587at7088 | Complete | Missing |
| 11589at7088 | Complete | Missing |
| 11592at7088 | Complete | Missing |
| 11593at7088 | Complete | Missing |
| 11594at7088 | Missing | Missing |
| 11597at7088 | Duplicated | Missing |
| 11598at7088 | Duplicated | Missing |
| 11599at7088 | Missing | Missing |
| 11607at7088 | Complete | Missing |
| 11608at7088 | Complete | Missing |
| 11609at7088 | Complete | Missing |
| 11616at7088 | Complete | Missing |
| 11619at7088 | Complete | Missing |
| 1161at7088 | Complete | Missing |
| 11621at7088 | Complete | Complete |
| 11630at7088 | Complete | Missing |
| 11633at7088 | Duplicated | Missing |
| 11638at7088 | Complete | Missing |
| 11644at7088 | Complete | Complete |
| 11648at7088 | Duplicated | Missing |
| 11650at7088 | Complete | Fragmented |
| 11655at7088 | Complete | Missing |
| 11656at7088 | Complete | Missing |
| 11657at7088 | Complete | Missing |
| 11659at7088 | Complete | Fragmented |
| 1165at7088 | Complete | Missing |
| 11660at7088 | Complete | Missing |
| 11662at7088 | Complete | Missing |
| 11664at7088 | Complete | Missing |
| 11665at7088 | Complete | Missing |
| 11666at7088 | Complete | Missing |
| 11669at7088 | Complete | Missing |
| 11672at7088 | Complete | Missing |
| 11677at7088 | Complete | Fragmented |
| 1167at7088 | Complete | Missing |
| 11681at7088 | Complete | Missing |
| 11687at7088 | Complete | Missing |
| 1168at7088 | Complete | Missing |
| 11693at7088 | Complete | Missing |
| 11699at7088 | Complete | Missing |
| 116at7088 | Complete | Missing |
| 11701at7088 | Complete | Missing |
| 11702at7088 | Complete | Missing |
| 11703at7088 | Complete | Missing |
| 11704at7088 | Complete | Missing |
| 11706at7088 | Complete | Missing |
| 11710at7088 | Complete | Missing |
| 11718at7088 | Complete | Missing |
| 11719at7088 | Complete | Missing |
| 1171at7088 | Complete | Missing |
| 11729at7088 | Complete | Missing |
| 1172at7088 | Duplicated | Complete |
| 11731at7088 | Duplicated | Complete |
| 1173at7088 | Complete | Missing |
| 11747at7088 | Complete | Missing |
| 1174at7088 | Complete | Missing |
| 11752at7088 | Complete | Missing |
| 11754at7088 | Complete | Missing |
| 11760at7088 | Complete | Missing |
| 11761at7088 | Complete | Missing |
| 11764at7088 | Complete | Missing |
| 11765at7088 | Complete | Missing |
| 11766at7088 | Complete | Missing |
| 11767at7088 | Complete | Missing |
| 11769at7088 | Complete | Missing |
| 11770at7088 | Complete | Missing |
| 11777at7088 | Complete | Missing |
| 11779at7088 | Complete | Missing |
| 11780at7088 | Complete | Missing |
| 11785at7088 | Complete | Missing |
| 11786at7088 | Complete | Missing |
| 11787at7088 | Complete | Missing |
| 1178at7088 | Complete | Missing |
| 11793at7088 | Complete | Missing |
| 11794at7088 | Complete | Missing |
| 11796at7088 | Complete | Missing |
| 11799at7088 | Complete | Missing |
| 1179at7088 | Complete | Missing |
| 11800at7088 | Missing | Missing |
| 11803at7088 | Complete | Missing |
| 1180at7088 | Missing | Missing |
| 11810at7088 | Complete | Missing |
| 11811at7088 | Complete | Missing |
| 11812at7088 | Complete | Missing |
| 11815at7088 | Complete | Missing |
| 11817at7088 | Complete | Missing |
| 11828at7088 | Complete | Missing |
| 1182at7088 | Complete | Missing |
| 11833at7088 | Complete | Missing |
| 11834at7088 | Complete | Missing |
| 11835at7088 | Complete | Missing |
| 11842at7088 | Complete | Missing |
| 11847at7088 | Complete | Complete |
| 11848at7088 | Complete | Missing |
| 11855at7088 | Complete | Missing |
| 11856at7088 | Complete | Missing |
| 11857at7088 | Duplicated | Missing |
| 11858at7088 | Complete | Missing |
| 11860at7088 | Fragmented | Missing |
| 11864at7088 | Complete | Missing |
| 11870at7088 | Missing | Missing |
| 11872at7088 | Missing | Missing |
| 11877at7088 | Complete | Missing |
| 1187at7088 | Complete | Missing |
| 11880at7088 | Complete | Missing |
| 11881at7088 | Complete | Missing |
| 11882at7088 | Complete | Missing |
| 11890at7088 | Complete | Missing |
| 11894at7088 | Complete | Missing |
| 11898at7088 | Complete | Missing |
| 11901at7088 | Duplicated | Missing |
| 11910at7088 | Complete | Missing |
| 11921at7088 | Complete | Missing |
| 11922at7088 | Complete | Missing |
| 11923at7088 | Complete | Missing |
| 11929at7088 | Complete | Missing |
| 11930at7088 | Complete | Missing |
| 11932at7088 | Duplicated | Missing |
| 11937at7088 | Complete | Missing |
| 11941at7088 | Complete | Missing |
| 11944at7088 | Complete | Complete |
| 11950at7088 | Complete | Fragmented |
| 11956at7088 | Complete | Missing |
| 11957at7088 | Complete | Missing |
| 11958at7088 | Complete | Missing |
| 11960at7088 | Complete | Missing |
| 11961at7088 | Fragmented | Missing |
| 11963at7088 | Complete | Missing |
| 11965at7088 | Complete | Missing |
| 11967at7088 | Complete | Missing |
| 11969at7088 | Duplicated | Missing |
| 11970at7088 | Missing | Missing |
| 11972at7088 | Complete | Missing |
| 11973at7088 | Duplicated | Missing |
| 11977at7088 | Complete | Missing |
| 11978at7088 | Complete | Missing |
| 1197at7088 | Complete | Missing |
| 11982at7088 | Duplicated | Missing |
| 11983at7088 | Complete | Missing |
| 11984at7088 | Complete | Missing |
| 11985at7088 | Complete | Missing |
| 1198at7088 | Complete | Missing |
| 11990at7088 | Complete | Missing |
| 11996at7088 | Complete | Missing |
| 11997at7088 | Complete | Missing |
| 1199at7088 | Complete | Missing |
| 11at7088 | Complete | Missing |
| 12003at7088 | Complete | Missing |
| 12004at7088 | Complete | Missing |
| 12006at7088 | Duplicated | Missing |
| 12008at7088 | Complete | Missing |
| 1200at7088 | Complete | Missing |
| 12011at7088 | Complete | Missing |
| 12012at7088 | Missing | Missing |
| 12013at7088 | Complete | Missing |
| 12016at7088 | Complete | Fragmented |
| 12017at7088 | Complete | Missing |
| 12021at7088 | Complete | Missing |
| 12023at7088 | Complete | Missing |
| 12024at7088 | Complete | Complete |
| 12026at7088 | Complete | Complete |
| 12028at7088 | Complete | Missing |
| 12029at7088 | Duplicated | Missing |
| 12030at7088 | Complete | Missing |
| 12031at7088 | Complete | Missing |
| 12036at7088 | Complete | Missing |
| 12043at7088 | Complete | Missing |
| 12054at7088 | Complete | Missing |
| 12055at7088 | Complete | Missing |
| 12056at7088 | Complete | Missing |
| 12058at7088 | Complete | Missing |
| 12059at7088 | Duplicated | Fragmented |
| 12060at7088 | Complete | Missing |
| 12061at7088 | Missing | Missing |
| 1206at7088 | Complete | Missing |
| 12070at7088 | Duplicated | Missing |
| 12073at7088 | Complete | Missing |
| 12075at7088 | Duplicated | Missing |
| 12076at7088 | Complete | Missing |
| 12082at7088 | Duplicated | Missing |
| 12084at7088 | Complete | Missing |
| 12087at7088 | Complete | Missing |
| 1208at7088 | Duplicated | Missing |
| 12090at7088 | Complete | Fragmented |
| 12091at7088 | Missing | Missing |
| 12093at7088 | Complete | Missing |
| 12094at7088 | Complete | Complete |
| 12095at7088 | Missing | Missing |
| 12096at7088 | Complete | Missing |
| 12101at7088 | Complete | Missing |
| 12113at7088 | Duplicated | Missing |
| 12117at7088 | Complete | Missing |
| 12120at7088 | Complete | Missing |
| 12123at7088 | Complete | Missing |
| 12125at7088 | Complete | Missing |
| 12127at7088 | Complete | Fragmented |
| 1212at7088 | Complete | Missing |
| 12133at7088 | Fragmented | Missing |
| 12136at7088 | Complete | Missing |
| 12139at7088 | Complete | Missing |
| 12140at7088 | Complete | Missing |
| 12141at7088 | Missing | Missing |
| 12143at7088 | Complete | Missing |
| 12148at7088 | Complete | Missing |
| 1214at7088 | Complete | Missing |
| 12152at7088 | Complete | Missing |
| 12153at7088 | Missing | Missing |
| 12154at7088 | Missing | Missing |
| 12164at7088 | Complete | Missing |
| 12173at7088 | Complete | Missing |
| 12174at7088 | Complete | Missing |
| 1217at7088 | Complete | Missing |
| 12180at7088 | Complete | Missing |
| 12183at7088 | Complete | Missing |
| 12184at7088 | Complete | Missing |
| 12185at7088 | Complete | Missing |
| 12187at7088 | Complete | Missing |
| 1218at7088 | Complete | Missing |
| 12193at7088 | Complete | Missing |
| 12198at7088 | Complete | Missing |
| 12204at7088 | Complete | Fragmented |
| 1220at7088 | Complete | Fragmented |
| 12210at7088 | Complete | Missing |
| 12212at7088 | Complete | Missing |
| 12217at7088 | Complete | Missing |
| 12218at7088 | Complete | Missing |
| 12219at7088 | Complete | Missing |
| 12221at7088 | Complete | Missing |
| 12223at7088 | Complete | Missing |
| 12225at7088 | Complete | Missing |
| 12226at7088 | Complete | Missing |
| 12229at7088 | Complete | Missing |
| 1222at7088 | Complete | Missing |
| 12233at7088 | Complete | Missing |
| 12235at7088 | Complete | Missing |
| 12237at7088 | Complete | Complete |
| 1223at7088 | Complete | Missing |
| 12241at7088 | Complete | Missing |
| 12243at7088 | Complete | Missing |
| 12251at7088 | Complete | Missing |
| 12254at7088 | Complete | Missing |
| 12264at7088 | Complete | Missing |
| 12268at7088 | Complete | Missing |
| 12271at7088 | Fragmented | Missing |
| 12273at7088 | Complete | Missing |
| 12274at7088 | Complete | Missing |
| 12275at7088 | Complete | Missing |
| 12276at7088 | Complete | Missing |
| 12293at7088 | Complete | Complete |
| 12295at7088 | Complete | Missing |
| 122at7088 | Complete | Missing |
| 12300at7088 | Complete | Missing |
| 12310at7088 | Complete | Missing |
| 12313at7088 | Complete | Missing |
| 12320at7088 | Complete | Missing |
| 12322at7088 | Complete | Missing |
| 12326at7088 | Complete | Missing |
| 12332at7088 | Complete | Missing |
| 12334at7088 | Complete | Missing |
| 12339at7088 | Complete | Missing |
| 12341at7088 | Complete | Missing |
| 12343at7088 | Complete | Missing |
| 12347at7088 | Missing | Missing |
| 12349at7088 | Complete | Missing |
| 1234at7088 | Fragmented | Missing |
| 12350at7088 | Complete | Missing |
| 12354at7088 | Complete | Missing |
| 12355at7088 | Complete | Missing |
| 1235at7088 | Complete | Missing |
| 12363at7088 | Complete | Missing |
| 12364at7088 | Complete | Missing |
| 12367at7088 | Complete | Missing |
| 12369at7088 | Complete | Missing |
| 1236at7088 | Complete | Fragmented |
| 12375at7088 | Complete | Missing |
| 12378at7088 | Complete | Missing |
| 1237at7088 | Complete | Missing |
| 12386at7088 | Complete | Missing |
| 12389at7088 | Complete | Missing |
| 12390at7088 | Duplicated | Fragmented |
| 12391at7088 | Complete | Missing |
| 12396at7088 | Duplicated | Missing |
| 12397at7088 | Duplicated | Missing |
| 123at7088 | Complete | Missing |
| 12400at7088 | Complete | Missing |
| 12401at7088 | Complete | Missing |
| 12403at7088 | Complete | Missing |
| 12406at7088 | Complete | Missing |
| 12413at7088 | Complete | Missing |
| 12416at7088 | Complete | Missing |
| 12418at7088 | Missing | Missing |
| 12419at7088 | Missing | Missing |
| 12420at7088 | Missing | Missing |
| 12422at7088 | Complete | Missing |
| 12426at7088 | Complete | Missing |
| 12427at7088 | Complete | Duplicated |
| 12429at7088 | Complete | Missing |
| 1242at7088 | Complete | Missing |
| 12442at7088 | Complete | Complete |
| 12443at7088 | Complete | Complete |
| 12444at7088 | Complete | Complete |
| 12445at7088 | Duplicated | Complete |
| 12449at7088 | Complete | Missing |
| 12450at7088 | Complete | Missing |
| 12453at7088 | Complete | Missing |
| 1245at7088 | Complete | Missing |
| 1246at7088 | Complete | Missing |
| 12471at7088 | Complete | Missing |
| 12472at7088 | Complete | Missing |
| 12481at7088 | Complete | Missing |
| 12483at7088 | Fragmented | Missing |
| 12484at7088 | Complete | Missing |
| 1248at7088 | Complete | Missing |
| 12492at7088 | Complete | Missing |
| 12496at7088 | Complete | Missing |
| 12503at7088 | Duplicated | Missing |
| 12504at7088 | Complete | Missing |
| 12505at7088 | Complete | Missing |
| 12507at7088 | Duplicated | Missing |
| 12508at7088 | Complete | Missing |
| 1250at7088 | Complete | Missing |
| 12510at7088 | Complete | Fragmented |
| 12517at7088 | Complete | Missing |
| 1251at7088 | Complete | Missing |
| 12530at7088 | Complete | Missing |
| 12531at7088 | Fragmented | Complete |
| 12535at7088 | Complete | Missing |
| 1253at7088 | Complete | Missing |
| 12540at7088 | Fragmented | Missing |
| 12542at7088 | Complete | Missing |
| 12543at7088 | Complete | Missing |
| 12544at7088 | Complete | Missing |
| 12547at7088 | Complete | Missing |
| 12550at7088 | Complete | Missing |
| 12562at7088 | Complete | Missing |
| 12564at7088 | Complete | Missing |
| 12566at7088 | Complete | Missing |
| 12567at7088 | Complete | Missing |
| 1256at7088 | Fragmented | Missing |
| 12572at7088 | Complete | Missing |
| 12574at7088 | Complete | Missing |
| 12576at7088 | Complete | Missing |
| 12585at7088 | Complete | Missing |
| 12589at7088 | Complete | Missing |
| 1258at7088 | Duplicated | Missing |
| 12598at7088 | Complete | Missing |
| 1259at7088 | Complete | Complete |
| 12603at7088 | Complete | Missing |
| 12605at7088 | Duplicated | Missing |
| 12607at7088 | Complete | Missing |
| 12609at7088 | Complete | Missing |
| 12613at7088 | Complete | Missing |
| 12617at7088 | Complete | Missing |
| 12618at7088 | Complete | Missing |
| 12626at7088 | Missing | Missing |
| 12628at7088 | Complete | Missing |
| 12632at7088 | Complete | Missing |
| 12633at7088 | Complete | Missing |
| 1263at7088 | Complete | Complete |
| 12640at7088 | Complete | Missing |
| 12646at7088 | Complete | Missing |
| 1264at7088 | Fragmented | Missing |
| 12653at7088 | Complete | Missing |
| 12656at7088 | Duplicated | Missing |
| 12660at7088 | Missing | Missing |
| 12668at7088 | Complete | Missing |
| 12669at7088 | Complete | Missing |
| 12671at7088 | Complete | Missing |
| 12673at7088 | Complete | Missing |
| 12676at7088 | Complete | Missing |
| 12679at7088 | Complete | Missing |
| 12680at7088 | Complete | Missing |
| 12691at7088 | Duplicated | Missing |
| 12696at7088 | Complete | Missing |
| 12699at7088 | Complete | Missing |
| 126at7088 | Complete | Missing |
| 12704at7088 | Duplicated | Missing |
| 12705at7088 | Complete | Missing |
| 12706at7088 | Complete | Missing |
| 12707at7088 | Complete | Missing |
| 12709at7088 | Complete | Missing |
| 12710at7088 | Complete | Missing |
| 12712at7088 | Complete | Missing |
| 12713at7088 | Fragmented | Missing |
| 12730at7088 | Duplicated | Missing |
| 12731at7088 | Complete | Missing |
| 12733at7088 | Duplicated | Missing |
| 12736at7088 | Complete | Missing |
| 12738at7088 | Complete | Missing |
| 1273at7088 | Complete | Missing |
| 12744at7088 | Complete | Missing |
| 12748at7088 | Complete | Missing |
| 1275at7088 | Complete | Missing |
| 12763at7088 | Complete | Complete |
| 12765at7088 | Complete | Missing |
| 12771at7088 | Complete | Missing |
| 12773at7088 | Complete | Missing |
| 12774at7088 | Complete | Missing |
| 12777at7088 | Complete | Missing |
| 12778at7088 | Complete | Missing |
| 12779at7088 | Complete | Complete |
| 12789at7088 | Complete | Missing |
| 1278at7088 | Complete | Missing |
| 12790at7088 | Missing | Duplicated |
| 12792at7088 | Complete | Missing |
| 127at7088 | Missing | Missing |
| 12804at7088 | Complete | Missing |
| 12810at7088 | Complete | Fragmented |
| 12813at7088 | Complete | Missing |
| 12815at7088 | Complete | Missing |
| 12817at7088 | Complete | Missing |
| 12819at7088 | Complete | Missing |
| 1281at7088 | Complete | Missing |
| 12820at7088 | Complete | Missing |
| 12821at7088 | Duplicated | Missing |
| 12829at7088 | Fragmented | Missing |
| 12830at7088 | Complete | Missing |
| 12831at7088 | Duplicated | Missing |
| 12834at7088 | Complete | Missing |
| 12838at7088 | Complete | Missing |
| 1283at7088 | Complete | Missing |
| 12842at7088 | Missing | Missing |
| 12847at7088 | Duplicated | Missing |
| 12861at7088 | Complete | Missing |
| 12883at7088 | Complete | Missing |
| 12884at7088 | Complete | Complete |
| 12885at7088 | Complete | Missing |
| 12891at7088 | Duplicated | Missing |
| 12892at7088 | Complete | Missing |
| 12893at7088 | Complete | Missing |
| 12894at7088 | Complete | Missing |
| 12895at7088 | Complete | Missing |
| 12896at7088 | Duplicated | Missing |
| 12899at7088 | Complete | Missing |
| 128at7088 | Complete | Missing |
| 12900at7088 | Missing | Missing |
| 12903at7088 | Missing | Missing |
| 12904at7088 | Complete | Missing |
| 12905at7088 | Complete | Missing |
| 12910at7088 | Complete | Missing |
| 12919at7088 | Complete | Missing |
| 1291at7088 | Complete | Missing |
| 12920at7088 | Complete | Missing |
| 12935at7088 | Complete | Missing |
| 12938at7088 | Fragmented | Missing |
| 12959at7088 | Missing | Missing |
| 1295at7088 | Complete | Missing |
| 12960at7088 | Duplicated | Missing |
| 12964at7088 | Complete | Missing |
| 12967at7088 | Complete | Missing |
| 1298at7088 | Complete | Missing |
| 12990at7088 | Complete | Missing |
| 12993at7088 | Complete | Missing |
| 12998at7088 | Complete | Missing |
| 1299at7088 | Complete | Missing |
| 13002at7088 | Complete | Missing |
| 13003at7088 | Complete | Missing |
| 13004at7088 | Duplicated | Missing |
| 13008at7088 | Complete | Missing |
| 1300at7088 | Complete | Missing |
| 13010at7088 | Complete | Missing |
| 13014at7088 | Duplicated | Missing |
| 13019at7088 | Complete | Missing |
| 13020at7088 | Complete | Missing |
| 13024at7088 | Complete | Missing |
| 13027at7088 | Complete | Missing |
| 13029at7088 | Complete | Missing |
| 13032at7088 | Complete | Missing |
| 13033at7088 | Complete | Missing |
| 13034at7088 | Complete | Missing |
| 13037at7088 | Complete | Missing |
| 13038at7088 | Complete | Missing |
| 13041at7088 | Complete | Missing |
| 13042at7088 | Complete | Missing |
| 13043at7088 | Complete | Missing |
| 13047at7088 | Complete | Fragmented |
| 13049at7088 | Complete | Missing |
| 1304at7088 | Complete | Missing |
| 13053at7088 | Complete | Missing |
| 13057at7088 | Complete | Missing |
| 13058at7088 | Complete | Missing |
| 13059at7088 | Complete | Missing |
| 13065at7088 | Complete | Missing |
| 13066at7088 | Missing | Missing |
| 13067at7088 | Complete | Missing |
| 1306at7088 | Complete | Missing |
| 13070at7088 | Complete | Missing |
| 13072at7088 | Complete | Missing |
| 13075at7088 | Complete | Missing |
| 1307at7088 | Missing | Missing |
| 13081at7088 | Complete | Missing |
| 13082at7088 | Complete | Missing |
| 13085at7088 | Complete | Missing |
| 13088at7088 | Complete | Missing |
| 13091at7088 | Missing | Missing |
| 13093at7088 | Complete | Missing |
| 13095at7088 | Duplicated | Missing |
| 13097at7088 | Complete | Missing |
| 13099at7088 | Complete | Missing |
| 13102at7088 | Complete | Missing |
| 13105at7088 | Complete | Missing |
| 13106at7088 | Complete | Missing |
| 13107at7088 | Complete | Missing |
| 13109at7088 | Complete | Missing |
| 13110at7088 | Complete | Missing |
| 13112at7088 | Duplicated | Missing |
| 13114at7088 | Complete | Missing |
| 13117at7088 | Complete | Missing |
| 13119at7088 | Complete | Missing |
| 13122at7088 | Complete | Missing |
| 13124at7088 | Complete | Missing |
| 13129at7088 | Complete | Missing |
| 13134at7088 | Complete | Missing |
| 1313at7088 | Complete | Missing |
| 13140at7088 | Complete | Missing |
| 13142at7088 | Complete | Missing |
| 1314at7088 | Complete | Missing |
| 13155at7088 | Complete | Missing |
| 13157at7088 | Complete | Missing |
| 13158at7088 | Complete | Missing |
| 13166at7088 | Duplicated | Complete |
| 13168at7088 | Complete | Missing |
| 13169at7088 | Complete | Missing |
| 13171at7088 | Complete | Missing |
| 13172at7088 | Complete | Missing |
| 13173at7088 | Complete | Missing |
| 13174at7088 | Complete | Missing |
| 1317at7088 | Complete | Missing |
| 13181at7088 | Complete | Missing |
| 13182at7088 | Complete | Missing |
| 13184at7088 | Complete | Missing |
| 13185at7088 | Complete | Missing |
| 13188at7088 | Complete | Missing |
| 13189at7088 | Complete | Missing |
| 1318at7088 | Complete | Missing |
| 13193at7088 | Complete | Missing |
| 13194at7088 | Complete | Missing |
| 13198at7088 | Complete | Missing |
| 13199at7088 | Duplicated | Missing |
| 1319at7088 | Complete | Missing |
| 13200at7088 | Complete | Missing |
| 1320at7088 | Complete | Missing |
| 13213at7088 | Complete | Missing |
| 13216at7088 | Complete | Missing |
| 13218at7088 | Complete | Missing |
| 13220at7088 | Complete | Missing |
| 13221at7088 | Fragmented | Missing |
| 13223at7088 | Complete | Missing |
| 13224at7088 | Complete | Complete |
| 13230at7088 | Duplicated | Missing |
| 13243at7088 | Complete | Missing |
| 1324at7088 | Complete | Missing |
| 13250at7088 | Complete | Missing |
| 13255at7088 | Complete | Missing |
| 13259at7088 | Complete | Missing |
| 1325at7088 | Duplicated | Missing |
| 13263at7088 | Complete | Missing |
| 13265at7088 | Complete | Missing |
| 13267at7088 | Complete | Missing |
| 1326at7088 | Complete | Missing |
| 13278at7088 | Complete | Missing |
| 13282at7088 | Complete | Missing |
| 13284at7088 | Complete | Missing |
| 13285at7088 | Complete | Missing |
| 13289at7088 | Complete | Missing |
| 13295at7088 | Complete | Complete |
| 13296at7088 | Missing | Missing |
| 13303at7088 | Complete | Missing |
| 13305at7088 | Complete | Missing |
| 13307at7088 | Complete | Fragmented |
| 13308at7088 | Complete | Missing |
| 1330at7088 | Complete | Missing |
| 13310at7088 | Duplicated | Missing |
| 13314at7088 | Complete | Missing |
| 13315at7088 | Fragmented | Missing |
| 13317at7088 | Missing | Missing |
| 13319at7088 | Duplicated | Missing |
| 13321at7088 | Complete | Missing |
| 13323at7088 | Duplicated | Missing |
| 13326at7088 | Complete | Missing |
| 13332at7088 | Complete | Missing |
| 13338at7088 | Complete | Missing |
| 13340at7088 | Complete | Missing |
| 13341at7088 | Complete | Missing |
| 13346at7088 | Complete | Missing |
| 13347at7088 | Complete | Missing |
| 13348at7088 | Complete | Missing |
| 13351at7088 | Complete | Complete |
| 13356at7088 | Complete | Missing |
| 13358at7088 | Complete | Missing |
| 1335at7088 | Complete | Missing |
| 13365at7088 | Complete | Missing |
| 13369at7088 | Complete | Missing |
| 1336at7088 | Complete | Missing |
| 13373at7088 | Complete | Missing |
| 13375at7088 | Complete | Missing |
| 13381at7088 | Complete | Missing |
| 13388at7088 | Complete | Missing |
| 1338at7088 | Complete | Missing |
| 13394at7088 | Complete | Missing |
| 13395at7088 | Complete | Missing |
| 13398at7088 | Complete | Missing |
| 13399at7088 | Complete | Missing |
| 133at7088 | Complete | Missing |
| 13406at7088 | Complete | Missing |
| 13411at7088 | Fragmented | Missing |
| 13413at7088 | Complete | Missing |
| 13414at7088 | Complete | Missing |
| 13421at7088 | Missing | Missing |
| 13424at7088 | Complete | Missing |
| 13425at7088 | Complete | Missing |
| 13435at7088 | Complete | Missing |
| 13437at7088 | Missing | Missing |
| 1343at7088 | Complete | Missing |
| 13443at7088 | Complete | Fragmented |
| 13445at7088 | Complete | Missing |
| 13447at7088 | Complete | Missing |
| 13451at7088 | Complete | Missing |
| 13463at7088 | Duplicated | Missing |
| 13464at7088 | Complete | Missing |
| 13465at7088 | Complete | Missing |
| 13467at7088 | Complete | Fragmented |
| 13468at7088 | Complete | Missing |
| 1346at7088 | Complete | Missing |
| 13478at7088 | Complete | Missing |
| 1347at7088 | Complete | Missing |
| 13481at7088 | Complete | Missing |
| 13483at7088 | Complete | Missing |
| 13484at7088 | Complete | Complete |
| 1348at7088 | Complete | Missing |
| 13495at7088 | Complete | Missing |
| 13500at7088 | Complete | Missing |
| 13501at7088 | Missing | Missing |
| 13504at7088 | Complete | Missing |
| 13506at7088 | Complete | Missing |
| 1350at7088 | Complete | Missing |
| 13519at7088 | Complete | Missing |
| 1351at7088 | Duplicated | Missing |
| 13527at7088 | Missing | Missing |
| 13528at7088 | Complete | Missing |
| 13530at7088 | Complete | Missing |
| 13532at7088 | Missing | Missing |
| 13533at7088 | Complete | Missing |
| 13534at7088 | Complete | Missing |
| 1353at7088 | Complete | Missing |
| 13549at7088 | Complete | Missing |
| 13554at7088 | Complete | Missing |
| 13555at7088 | Complete | Missing |
| 1355at7088 | Complete | Missing |
| 13561at7088 | Complete | Missing |
| 13565at7088 | Missing | Missing |
| 13568at7088 | Duplicated | Missing |
| 1356at7088 | Complete | Missing |
| 13582at7088 | Complete | Missing |
| 13586at7088 | Complete | Complete |
| 1358at7088 | Complete | Missing |
| 13592at7088 | Complete | Missing |
| 13596at7088 | Complete | Missing |
| 13602at7088 | Complete | Missing |
| 13603at7088 | Complete | Missing |
| 13604at7088 | Complete | Missing |
| 13607at7088 | Duplicated | Missing |
| 13608at7088 | Fragmented | Missing |
| 1360at7088 | Complete | Missing |
| 13612at7088 | Complete | Missing |
| 13617at7088 | Missing | Missing |
| 13620at7088 | Complete | Missing |
| 13623at7088 | Complete | Missing |
| 1362at7088 | Complete | Missing |
| 13634at7088 | Complete | Missing |
| 13637at7088 | Complete | Missing |
| 13638at7088 | Complete | Missing |
| 13644at7088 | Duplicated | Missing |
| 13651at7088 | Complete | Missing |
| 13654at7088 | Complete | Missing |
| 13655at7088 | Complete | Missing |
| 13664at7088 | Complete | Missing |
| 1366at7088 | Complete | Missing |
| 13670at7088 | Complete | Missing |
| 13677at7088 | Complete | Missing |
| 13682at7088 | Complete | Missing |
| 13687at7088 | Complete | Missing |
| 1368at7088 | Complete | Missing |
| 13698at7088 | Complete | Missing |
| 1369at7088 | Complete | Missing |
| 136at7088 | Complete | Missing |
| 13703at7088 | Complete | Missing |
| 13710at7088 | Complete | Missing |
| 13714at7088 | Duplicated | Missing |
| 13718at7088 | Complete | Missing |
| 13724at7088 | Complete | Missing |
| 1372at7088 | Complete | Missing |
| 13730at7088 | Complete | Missing |
| 13731at7088 | Complete | Missing |
| 13733at7088 | Missing | Missing |
| 13737at7088 | Complete | Missing |
| 13748at7088 | Duplicated | Missing |
| 13753at7088 | Complete | Missing |
| 13755at7088 | Complete | Missing |
| 13761at7088 | Duplicated | Missing |
| 13763at7088 | Complete | Missing |
| 13774at7088 | Complete | Missing |
| 13775at7088 | Complete | Missing |
| 13777at7088 | Complete | Missing |
| 1377at7088 | Duplicated | Missing |
| 13782at7088 | Duplicated | Missing |
| 13787at7088 | Complete | Missing |
| 13790at7088 | Complete | Missing |
| 13793at7088 | Duplicated | Missing |
| 13796at7088 | Fragmented | Missing |
| 13798at7088 | Complete | Missing |
| 137at7088 | Complete | Missing |
| 13803at7088 | Missing | Missing |
| 13805at7088 | Missing | Missing |
| 13808at7088 | Missing | Missing |
| 13809at7088 | Complete | Missing |
| 1380at7088 | Complete | Missing |
| 13811at7088 | Complete | Missing |
| 13813at7088 | Complete | Missing |
| 13815at7088 | Complete | Missing |
| 13829at7088 | Complete | Missing |
| 1382at7088 | Complete | Missing |
| 13834at7088 | Complete | Missing |
| 13858at7088 | Complete | Missing |
| 13859at7088 | Complete | Missing |
| 13870at7088 | Complete | Missing |
| 13877at7088 | Complete | Missing |
| 1388at7088 | Complete | Missing |
| 13890at7088 | Complete | Missing |
| 13897at7088 | Complete | Missing |
| 138at7088 | Complete | Missing |
| 13902at7088 | Complete | Missing |
| 13904at7088 | Complete | Missing |
| 13907at7088 | Fragmented | Missing |
| 1390at7088 | Complete | Missing |
| 13916at7088 | Complete | Missing |
| 1391at7088 | Complete | Missing |
| 1392at7088 | Complete | Missing |
| 13936at7088 | Complete | Missing |
| 13941at7088 | Complete | Missing |
| 13943at7088 | Complete | Missing |
| 13947at7088 | Complete | Missing |
| 13949at7088 | Missing | Missing |
| 13952at7088 | Complete | Missing |
| 13955at7088 | Complete | Missing |
| 1395at7088 | Complete | Missing |
| 13961at7088 | Complete | Missing |
| 1396at7088 | Complete | Missing |
| 13972at7088 | Complete | Missing |
| 13977at7088 | Missing | Missing |
| 1397at7088 | Complete | Missing |
| 13984at7088 | Complete | Missing |
| 13986at7088 | Complete | Fragmented |
| 13989at7088 | Complete | Missing |
| 1398at7088 | Complete | Missing |
| 13991at7088 | Complete | Missing |
| 13994at7088 | Complete | Fragmented |
| 13995at7088 | Complete | Missing |
| 13998at7088 | Complete | Missing |
| 139at7088 | Complete | Missing |
| 13at7088 | Complete | Missing |
| 14000at7088 | Missing | Missing |
| 14009at7088 | Fragmented | Missing |
| 14011at7088 | Complete | Missing |
| 14013at7088 | Complete | Missing |
| 14016at7088 | Duplicated | Missing |
| 14018at7088 | Complete | Missing |
| 1401at7088 | Complete | Fragmented |
| 14028at7088 | Complete | Missing |
| 14032at7088 | Complete | Missing |
| 14037at7088 | Complete | Missing |
| 1403at7088 | Complete | Missing |
| 14046at7088 | Duplicated | Missing |
| 14061at7088 | Complete | Missing |
| 14069at7088 | Duplicated | Missing |
| 14070at7088 | Complete | Missing |
| 14072at7088 | Complete | Missing |
| 14075at7088 | Complete | Missing |
| 1407at7088 | Complete | Missing |
| 14082at7088 | Complete | Complete |
| 14083at7088 | Complete | Missing |
| 14094at7088 | Complete | Fragmented |
| 14098at7088 | Duplicated | Missing |
| 140at7088 | Complete | Missing |
| 14101at7088 | Complete | Missing |
| 14104at7088 | Missing | Missing |
| 14105at7088 | Complete | Missing |
| 14106at7088 | Complete | Missing |
| 14107at7088 | Complete | Missing |
| 14112at7088 | Complete | Missing |
| 14119at7088 | Duplicated | Missing |
| 1411at7088 | Complete | Fragmented |
| 14123at7088 | Complete | Missing |
| 14127at7088 | Duplicated | Missing |
| 14130at7088 | Complete | Missing |
| 14132at7088 | Complete | Missing |
| 14137at7088 | Complete | Missing |
| 14138at7088 | Duplicated | Missing |
| 14139at7088 | Complete | Missing |
| 1413at7088 | Complete | Missing |
| 14141at7088 | Complete | Missing |
| 14144at7088 | Complete | Missing |
| 14147at7088 | Complete | Missing |
| 14149at7088 | Complete | Missing |
| 14156at7088 | Complete | Missing |
| 14165at7088 | Complete | Missing |
| 14167at7088 | Complete | Missing |
| 14175at7088 | Duplicated | Missing |
| 14176at7088 | Complete | Missing |
| 14177at7088 | Complete | Missing |
| 14179at7088 | Complete | Missing |
| 1417at7088 | Complete | Missing |
| 14181at7088 | Complete | Missing |
| 14184at7088 | Complete | Missing |
| 14186at7088 | Complete | Missing |
| 1418at7088 | Complete | Missing |
| 14190at7088 | Complete | Missing |
| 14194at7088 | Complete | Duplicated |
| 1419at7088 | Duplicated | Missing |
| 14203at7088 | Complete | Duplicated |
| 14205at7088 | Complete | Missing |
| 14213at7088 | Complete | Missing |
| 14214at7088 | Complete | Missing |
| 14216at7088 | Missing | Missing |
| 1421at7088 | Complete | Fragmented |
| 14229at7088 | Complete | Missing |
| 1422at7088 | Complete | Missing |
| 14230at7088 | Complete | Missing |
| 14231at7088 | Complete | Missing |
| 14232at7088 | Duplicated | Missing |
| 14239at7088 | Complete | Missing |
| 14240at7088 | Complete | Missing |
| 14245at7088 | Complete | Missing |
| 14247at7088 | Duplicated | Complete |
| 14249at7088 | Complete | Missing |
| 14250at7088 | Complete | Missing |
| 14251at7088 | Complete | Missing |
| 14261at7088 | Complete | Missing |
| 14265at7088 | Complete | Complete |
| 14273at7088 | Complete | Missing |
| 14278at7088 | Missing | Missing |
| 14284at7088 | Complete | Missing |
| 14287at7088 | Complete | Missing |
| 14288at7088 | Complete | Missing |
| 14290at7088 | Duplicated | Missing |
| 14292at7088 | Fragmented | Missing |
| 14294at7088 | Complete | Missing |
| 14296at7088 | Complete | Missing |
| 14309at7088 | Complete | Missing |
| 14313at7088 | Complete | Missing |
| 14314at7088 | Complete | Missing |
| 14318at7088 | Complete | Missing |
| 14319at7088 | Complete | Missing |
| 1431at7088 | Complete | Fragmented |
| 14326at7088 | Complete | Missing |
| 14327at7088 | Complete | Missing |
| 1432at7088 | Complete | Missing |
| 14331at7088 | Complete | Missing |
| 14332at7088 | Complete | Missing |
| 14333at7088 | Complete | Missing |
| 14334at7088 | Complete | Missing |
| 14335at7088 | Complete | Missing |
| 14336at7088 | Complete | Missing |
| 14338at7088 | Complete | Missing |
| 14339at7088 | Complete | Missing |
| 14343at7088 | Complete | Missing |
| 14346at7088 | Missing | Missing |
| 14348at7088 | Complete | Missing |
| 14349at7088 | Complete | Missing |
| 14355at7088 | Complete | Missing |
| 14365at7088 | Complete | Missing |
| 14369at7088 | Complete | Missing |
| 1436at7088 | Complete | Missing |
| 14372at7088 | Complete | Missing |
| 1437at7088 | Complete | Missing |
| 14389at7088 | Missing | Missing |
| 14391at7088 | Complete | Missing |
| 14393at7088 | Complete | Missing |
| 14395at7088 | Fragmented | Missing |
| 14403at7088 | Complete | Missing |
| 14404at7088 | Missing | Missing |
| 14406at7088 | Complete | Missing |
| 1440at7088 | Complete | Missing |
| 14417at7088 | Complete | Missing |
| 14418at7088 | Complete | Missing |
| 1441at7088 | Missing | Missing |
| 14421at7088 | Complete | Missing |
| 14422at7088 | Complete | Missing |
| 14424at7088 | Complete | Missing |
| 1442at7088 | Missing | Missing |
| 14433at7088 | Complete | Missing |
| 1443at7088 | Complete | Missing |
| 14443at7088 | Missing | Missing |
| 14444at7088 | Complete | Missing |
| 14449at7088 | Missing | Missing |
| 1444at7088 | Duplicated | Missing |
| 14451at7088 | Duplicated | Missing |
| 14456at7088 | Complete | Missing |
| 14465at7088 | Complete | Missing |
| 14467at7088 | Complete | Missing |
| 14468at7088 | Missing | Duplicated |
| 1446at7088 | Complete | Missing |
| 14475at7088 | Complete | Missing |
| 14495at7088 | Duplicated | Missing |
| 14497at7088 | Duplicated | Missing |
| 1449at7088 | Fragmented | Missing |
| 14510at7088 | Complete | Missing |
| 14511at7088 | Complete | Missing |
| 14512at7088 | Duplicated | Missing |
| 14514at7088 | Complete | Missing |
| 1451at7088 | Complete | Missing |
| 14521at7088 | Complete | Missing |
| 14524at7088 | Complete | Missing |
| 14530at7088 | Complete | Missing |
| 14532at7088 | Duplicated | Missing |
| 14536at7088 | Complete | Missing |
| 1453at7088 | Complete | Missing |
| 14540at7088 | Missing | Missing |
| 14548at7088 | Complete | Missing |
| 14549at7088 | Complete | Missing |
| 14556at7088 | Missing | Missing |
| 14566at7088 | Complete | Missing |
| 14573at7088 | Complete | Missing |
| 14577at7088 | Duplicated | Missing |
| 14580at7088 | Complete | Missing |
| 14582at7088 | Complete | Missing |
| 14592at7088 | Missing | Missing |
| 14593at7088 | Complete | Missing |
| 14594at7088 | Complete | Missing |
| 14598at7088 | Complete | Missing |
| 14599at7088 | Complete | Missing |
| 1459at7088 | Duplicated | Missing |
| 145at7088 | Complete | Missing |
| 14604at7088 | Missing | Missing |
| 14614at7088 | Complete | Missing |
| 1461at7088 | Complete | Fragmented |
| 14621at7088 | Missing | Missing |
| 14623at7088 | Complete | Missing |
| 14628at7088 | Complete | Missing |
| 14633at7088 | Complete | Missing |
| 14636at7088 | Complete | Missing |
| 1463at7088 | Complete | Missing |
| 14641at7088 | Complete | Fragmented |
| 14653at7088 | Complete | Missing |
| 14655at7088 | Complete | Missing |
| 1465at7088 | Complete | Missing |
| 14664at7088 | Duplicated | Missing |
| 14668at7088 | Duplicated | Missing |
| 14675at7088 | Complete | Missing |
| 14687at7088 | Complete | Missing |
| 14688at7088 | Complete | Missing |
| 14689at7088 | Complete | Missing |
| 1468at7088 | Fragmented | Missing |
| 14690at7088 | Complete | Missing |
| 14697at7088 | Complete | Missing |
| 14698at7088 | Complete | Missing |
| 1469at7088 | Complete | Missing |
| 14701at7088 | Complete | Missing |
| 14706at7088 | Complete | Missing |
| 14709at7088 | Complete | Missing |
| 14711at7088 | Missing | Missing |
| 14714at7088 | Complete | Missing |
| 14724at7088 | Complete | Missing |
| 14727at7088 | Complete | Missing |
| 14729at7088 | Complete | Missing |
| 1472at7088 | Complete | Missing |
| 14735at7088 | Complete | Missing |
| 14738at7088 | Complete | Missing |
| 14739at7088 | Missing | Missing |
| 1473at7088 | Complete | Fragmented |
| 14740at7088 | Fragmented | Missing |
| 14749at7088 | Complete | Missing |
| 14753at7088 | Complete | Missing |
| 14755at7088 | Duplicated | Missing |
| 14758at7088 | Complete | Missing |
| 14762at7088 | Duplicated | Missing |
| 1477at7088 | Complete | Missing |
| 14783at7088 | Complete | Complete |
| 14785at7088 | Duplicated | Missing |
| 14798at7088 | Complete | Missing |
| 1479at7088 | Duplicated | Missing |
| 14814at7088 | Complete | Missing |
| 14815at7088 | Complete | Missing |
| 14829at7088 | Complete | Missing |
| 14831at7088 | Complete | Missing |
| 14835at7088 | Missing | Missing |
| 14836at7088 | Duplicated | Missing |
| 14844at7088 | Complete | Missing |
| 14845at7088 | Complete | Missing |
| 14849at7088 | Duplicated | Missing |
| 14850at7088 | Complete | Missing |
| 14870at7088 | Duplicated | Missing |
| 14883at7088 | Complete | Missing |
| 14886at7088 | Complete | Missing |
| 14887at7088 | Complete | Missing |
| 14889at7088 | Complete | Missing |
| 1488at7088 | Complete | Missing |
| 14894at7088 | Complete | Missing |
| 14895at7088 | Complete | Missing |
| 14896at7088 | Complete | Missing |
| 1489at7088 | Complete | Missing |
| 148at7088 | Fragmented | Missing |
| 14909at7088 | Complete | Missing |
| 1490at7088 | Complete | Fragmented |
| 14916at7088 | Complete | Missing |
| 14918at7088 | Fragmented | Missing |
| 14920at7088 | Complete | Complete |
| 14928at7088 | Complete | Complete |
| 14930at7088 | Duplicated | Missing |
| 14938at7088 | Fragmented | Missing |
| 14940at7088 | Duplicated | Missing |
| 14943at7088 | Complete | Complete |
| 14944at7088 | Complete | Missing |
| 14948at7088 | Complete | Missing |
| 14949at7088 | Complete | Missing |
| 14955at7088 | Complete | Missing |
| 14960at7088 | Complete | Missing |
| 1496at7088 | Complete | Missing |
| 14971at7088 | Complete | Missing |
| 14973at7088 | Complete | Missing |
| 14982at7088 | Complete | Missing |
| 14986at7088 | Complete | Missing |
| 14987at7088 | Complete | Missing |
| 14988at7088 | Complete | Missing |
| 14995at7088 | Fragmented | Missing |
| 14998at7088 | Duplicated | Missing |
| 15003at7088 | Complete | Missing |
| 15007at7088 | Missing | Fragmented |
| 1500at7088 | Complete | Missing |
| 15010at7088 | Complete | Missing |
| 15011at7088 | Complete | Missing |
| 15016at7088 | Complete | Missing |
| 15019at7088 | Complete | Missing |
| 1501at7088 | Fragmented | Missing |
| 15035at7088 | Complete | Complete |
| 15036at7088 | Missing | Missing |
| 15039at7088 | Complete | Missing |
| 1503at7088 | Complete | Missing |
| 15043at7088 | Complete | Missing |
| 1504at7088 | Missing | Missing |
| 15050at7088 | Missing | Missing |
| 15055at7088 | Complete | Missing |
| 1505at7088 | Complete | Complete |
| 15065at7088 | Missing | Missing |
| 1506at7088 | Complete | Missing |
| 15078at7088 | Complete | Missing |
| 1507at7088 | Complete | Missing |
| 15080at7088 | Missing | Missing |
| 15087at7088 | Complete | Missing |
| 1508at7088 | Complete | Fragmented |
| 15091at7088 | Complete | Missing |
| 1509at7088 | Complete | Missing |
| 15100at7088 | Complete | Missing |
| 15107at7088 | Complete | Missing |
| 15109at7088 | Complete | Fragmented |
| 1510at7088 | Complete | Missing |
| 15116at7088 | Fragmented | Missing |
| 15117at7088 | Missing | Missing |
| 15118at7088 | Missing | Missing |
| 15119at7088 | Complete | Missing |
| 1511at7088 | Complete | Missing |
| 15123at7088 | Complete | Missing |
| 15125at7088 | Fragmented | Missing |
| 15126at7088 | Complete | Missing |
| 15136at7088 | Complete | Missing |
| 15145at7088 | Complete | Missing |
| 15149at7088 | Complete | Missing |
| 1514at7088 | Complete | Missing |
| 15151at7088 | Complete | Complete |
| 1515at7088 | Complete | Missing |
| 15162at7088 | Duplicated | Missing |
| 15172at7088 | Complete | Missing |
| 15186at7088 | Complete | Missing |
| 15190at7088 | Complete | Missing |
| 15191at7088 | Complete | Missing |
| 15192at7088 | Complete | Missing |
| 15193at7088 | Complete | Missing |
| 15195at7088 | Complete | Missing |
| 1519at7088 | Complete | Missing |
| 15205at7088 | Complete | Missing |
| 15207at7088 | Complete | Missing |
| 15219at7088 | Complete | Missing |
| 15223at7088 | Complete | Missing |
| 15225at7088 | Complete | Missing |
| 15226at7088 | Complete | Missing |
| 15234at7088 | Complete | Missing |
| 15237at7088 | Duplicated | Missing |
| 1523at7088 | Duplicated | Missing |
| 15240at7088 | Complete | Missing |
| 15241at7088 | Complete | Complete |
| 15247at7088 | Complete | Missing |
| 15248at7088 | Missing | Missing |
| 1525at7088 | Complete | Missing |
| 15262at7088 | Missing | Missing |
| 15269at7088 | Duplicated | Complete |
| 15270at7088 | Complete | Missing |
| 15271at7088 | Complete | Missing |
| 15272at7088 | Complete | Missing |
| 15278at7088 | Duplicated | Missing |
| 15284at7088 | Complete | Missing |
| 15289at7088 | Complete | Missing |
| 15291at7088 | Complete | Missing |
| 15293at7088 | Complete | Missing |
| 1530at7088 | Missing | Missing |
| 15311at7088 | Complete | Missing |
| 15312at7088 | Complete | Missing |
| 1531at7088 | Complete | Missing |
| 15320at7088 | Complete | Missing |
| 15325at7088 | Complete | Missing |
| 1532at7088 | Fragmented | Missing |
| 15337at7088 | Missing | Missing |
| 15342at7088 | Complete | Missing |
| 15355at7088 | Complete | Missing |
| 15368at7088 | Complete | Fragmented |
| 15379at7088 | Duplicated | Missing |
| 1537at7088 | Complete | Missing |
| 15382at7088 | Complete | Missing |
| 15405at7088 | Complete | Missing |
| 15435at7088 | Complete | Missing |
| 15437at7088 | Complete | Missing |
| 1543at7088 | Complete | Missing |
| 15440at7088 | Complete | Missing |
| 1544at7088 | Complete | Missing |
| 15450at7088 | Complete | Missing |
| 15453at7088 | Complete | Missing |
| 1545at7088 | Missing | Fragmented |
| 15461at7088 | Complete | Missing |
| 15464at7088 | Fragmented | Missing |
| 15470at7088 | Complete | Missing |
| 1547at7088 | Complete | Missing |
| 15484at7088 | Complete | Missing |
| 15488at7088 | Missing | Missing |
| 1549at7088 | Complete | Missing |
| 15500at7088 | Complete | Missing |
| 15506at7088 | Missing | Missing |
| 15508at7088 | Complete | Missing |
| 15509at7088 | Complete | Missing |
| 1550at7088 | Complete | Missing |
| 15518at7088 | Complete | Missing |
| 1551at7088 | Complete | Missing |
| 15522at7088 | Complete | Missing |
| 15525at7088 | Complete | Missing |
| 15539at7088 | Missing | Complete |
| 1553at7088 | Duplicated | Missing |
| 15552at7088 | Complete | Missing |
| 1555at7088 | Complete | Missing |
| 15565at7088 | Complete | Missing |
| 15566at7088 | Complete | Missing |
| 15570at7088 | Complete | Missing |
| 1557at7088 | Complete | Missing |
| 15581at7088 | Missing | Missing |
| 15583at7088 | Complete | Missing |
| 1558at7088 | Complete | Missing |
| 15593at7088 | Duplicated | Missing |
| 155at7088 | Complete | Missing |
| 15613at7088 | Duplicated | Complete |
| 15617at7088 | Complete | Fragmented |
| 15626at7088 | Missing | Missing |
| 1562at7088 | Complete | Missing |
| 15633at7088 | Duplicated | Missing |
| 15635at7088 | Complete | Missing |
| 1563at7088 | Complete | Missing |
| 15648at7088 | Complete | Missing |
| 1564at7088 | Missing | Missing |
| 15652at7088 | Complete | Missing |
| 1565at7088 | Complete | Missing |
| 1566at7088 | Complete | Missing |
| 15672at7088 | Missing | Missing |
| 15673at7088 | Complete | Fragmented |
| 1567at7088 | Complete | Missing |
| 15680at7088 | Complete | Missing |
| 1568at7088 | Complete | Missing |
| 15695at7088 | Missing | Missing |
| 15704at7088 | Duplicated | Missing |
| 15705at7088 | Complete | Missing |
| 15707at7088 | Complete | Missing |
| 1571at7088 | Complete | Missing |
| 15737at7088 | Complete | Missing |
| 15738at7088 | Complete | Missing |
| 1574at7088 | Complete | Missing |
| 15754at7088 | Complete | Missing |
| 15758at7088 | Complete | Missing |
| 1575at7088 | Complete | Missing |
| 1576at7088 | Complete | Missing |
| 15771at7088 | Complete | Missing |
| 15779at7088 | Complete | Complete |
| 1577at7088 | Complete | Missing |
| 15781at7088 | Complete | Complete |
| 15805at7088 | Complete | Missing |
| 15808at7088 | Complete | Fragmented |
| 15816at7088 | Fragmented | Missing |
| 15819at7088 | Complete | Missing |
| 15831at7088 | Complete | Missing |
| 1583at7088 | Duplicated | Missing |
| 15853at7088 | Complete | Missing |
| 15860at7088 | Complete | Missing |
| 15864at7088 | Complete | Missing |
| 1586at7088 | Complete | Complete |
| 1587at7088 | Complete | Missing |
| 15894at7088 | Fragmented | Missing |
| 1589at7088 | Complete | Missing |
| 158at7088 | Complete | Missing |
| 1590at7088 | Complete | Missing |
| 1591at7088 | Complete | Missing |
| 15922at7088 | Complete | Missing |
| 1592at7088 | Complete | Missing |
| 15939at7088 | Complete | Missing |
| 15940at7088 | Complete | Missing |
| 1594at7088 | Complete | Missing |
| 1595at7088 | Complete | Missing |
| 15966at7088 | Complete | Missing |
| 1596at7088 | Complete | Missing |
| 15973at7088 | Complete | Missing |
| 1598at7088 | Complete | Missing |
| 159at7088 | Complete | Missing |
| 16009at7088 | Complete | Missing |
| 1600at7088 | Complete | Missing |
| 16011at7088 | Duplicated | Complete |
| 16012at7088 | Complete | Missing |
| 1601at7088 | Complete | Missing |
| 16028at7088 | Duplicated | Missing |
| 16035at7088 | Complete | Missing |
| 16049at7088 | Complete | Missing |
| 1605at7088 | Complete | Missing |
| 16066at7088 | Missing | Missing |
| 16069at7088 | Missing | Missing |
| 1606at7088 | Complete | Fragmented |
| 16071at7088 | Complete | Missing |
| 1607at7088 | Complete | Missing |
| 16088at7088 | Complete | Missing |
| 1608at7088 | Complete | Missing |
| 1609at7088 | Complete | Missing |
| 160at7088 | Complete | Missing |
| 16103at7088 | Duplicated | Missing |
| 16112at7088 | Complete | Missing |
| 16115at7088 | Complete | Missing |
| 1612at7088 | Duplicated | Complete |
| 1613at7088 | Complete | Missing |
| 16140at7088 | Complete | Missing |
| 1614at7088 | Complete | Missing |
| 16159at7088 | Complete | Missing |
| 16168at7088 | Missing | Missing |
| 16180at7088 | Duplicated | Missing |
| 16196at7088 | Complete | Missing |
| 1619at7088 | Complete | Missing |
| 1623at7088 | Complete | Missing |
| 16248at7088 | Complete | Missing |
| 16250at7088 | Complete | Missing |
| 16252at7088 | Complete | Missing |
| 1625at7088 | Complete | Missing |
| 1626at7088 | Duplicated | Missing |
| 1627at7088 | Complete | Missing |
| 16282at7088 | Complete | Missing |
| 1628at7088 | Fragmented | Fragmented |
| 16293at7088 | Complete | Missing |
| 16306at7088 | Complete | Missing |
| 16335at7088 | Duplicated | Missing |
| 1635at7088 | Complete | Missing |
| 1636at7088 | Complete | Missing |
| 1637at7088 | Complete | Missing |
| 1638at7088 | Complete | Missing |
| 163at7088 | Complete | Missing |
| 16400at7088 | Complete | Complete |
| 1641at7088 | Complete | Missing |
| 1642at7088 | Complete | Missing |
| 1644at7088 | Complete | Missing |
| 1645at7088 | Complete | Missing |
| 16468at7088 | Complete | Missing |
| 1647at7088 | Complete | Missing |
| 1648at7088 | Complete | Missing |
| 1650at7088 | Missing | Missing |
| 16513at7088 | Complete | Missing |
| 16527at7088 | Missing | Missing |
| 1652at7088 | Missing | Missing |
| 1657at7088 | Complete | Missing |
| 16607at7088 | Complete | Missing |
| 1660at7088 | Duplicated | Fragmented |
| 1661at7088 | Complete | Missing |
| 1662at7088 | Complete | Missing |
| 16641at7088 | Complete | Complete |
| 16647at7088 | Complete | Missing |
| 1665at7088 | Complete | Missing |
| 16663at7088 | Complete | Missing |
| 16668at7088 | Missing | Missing |
| 1667at7088 | Complete | Missing |
| 1670at7088 | Complete | Missing |
| 16739at7088 | Missing | Missing |
| 1678at7088 | Fragmented | Missing |
| 1679at7088 | Complete | Missing |
| 1681at7088 | Complete | Missing |
| 1682at7088 | Complete | Missing |
| 1683at7088 | Complete | Missing |
| 1684at7088 | Complete | Missing |
| 16855at7088 | Complete | Missing |
| 1685at7088 | Complete | Missing |
| 1686at7088 | Complete | Missing |
| 1687at7088 | Complete | Missing |
| 16938at7088 | Missing | Missing |
| 1693at7088 | Duplicated | Missing |
| 1695at7088 | Missing | Missing |
| 1696at7088 | Complete | Missing |
| 16982at7088 | Complete | Missing |
| 1699at7088 | Complete | Missing |
| 1700at7088 | Complete | Missing |
| 1701at7088 | Complete | Missing |
| 1705at7088 | Complete | Missing |
| 1707at7088 | Missing | Missing |
| 1709at7088 | Complete | Missing |
| 1711at7088 | Duplicated | Missing |
| 1718at7088 | Complete | Missing |
| 1723at7088 | Complete | Missing |
| 1724at7088 | Complete | Missing |
| 1725at7088 | Complete | Missing |
| 1728at7088 | Complete | Missing |
| 1729at7088 | Complete | Missing |
| 1731at7088 | Complete | Missing |
| 1734at7088 | Complete | Missing |
| 1735at7088 | Complete | Missing |
| 1738at7088 | Complete | Missing |
| 173at7088 | Complete | Missing |
| 1742at7088 | Complete | Missing |
| 1744at7088 | Complete | Missing |
| 1745at7088 | Complete | Missing |
| 1746at7088 | Complete | Missing |
| 1750at7088 | Complete | Missing |
| 1752at7088 | Complete | Missing |
| 1753at7088 | Complete | Missing |
| 1755at7088 | Complete | Missing |
| 1758at7088 | Complete | Missing |
| 1760at7088 | Complete | Fragmented |
| 1761at7088 | Fragmented | Missing |
| 1764at7088 | Complete | Missing |
| 1768at7088 | Complete | Missing |
| 1770at7088 | Fragmented | Missing |
| 1772at7088 | Complete | Missing |
| 1775at7088 | Complete | Missing |
| 177at7088 | Complete | Missing |
| 1783at7088 | Complete | Missing |
| 1785at7088 | Complete | Missing |
| 1787at7088 | Complete | Missing |
| 1789at7088 | Complete | Missing |
| 1793at7088 | Duplicated | Missing |
| 179at7088 | Complete | Missing |
| 1800at7088 | Complete | Missing |
| 1801at7088 | Complete | Missing |
| 1806at7088 | Complete | Missing |
| 1809at7088 | Complete | Missing |
| 1810at7088 | Complete | Fragmented |
| 1811at7088 | Complete | Missing |
| 1812at7088 | Complete | Missing |
| 1819at7088 | Complete | Missing |
| 1822at7088 | Complete | Missing |
| 1826at7088 | Complete | Missing |
| 1827at7088 | Complete | Missing |
| 1828at7088 | Complete | Fragmented |
| 1829at7088 | Complete | Missing |
| 1835at7088 | Duplicated | Missing |
| 1836at7088 | Complete | Missing |
| 1837at7088 | Complete | Missing |
| 183at7088 | Missing | Missing |
| 1841at7088 | Complete | Missing |
| 1845at7088 | Complete | Missing |
| 1846at7088 | Missing | Missing |
| 1847at7088 | Complete | Missing |
| 184at7088 | Complete | Missing |
| 1851at7088 | Complete | Missing |
| 1857at7088 | Complete | Missing |
| 1862at7088 | Complete | Missing |
| 1865at7088 | Fragmented | Missing |
| 1868at7088 | Missing | Missing |
| 1869at7088 | Complete | Missing |
| 186at7088 | Complete | Missing |
| 1871at7088 | Complete | Missing |
| 1872at7088 | Fragmented | Missing |
| 1873at7088 | Complete | Missing |
| 1874at7088 | Complete | Missing |
| 1881at7088 | Complete | Missing |
| 1882at7088 | Complete | Missing |
| 1884at7088 | Complete | Missing |
| 1885at7088 | Complete | Missing |
| 1889at7088 | Complete | Missing |
| 1890at7088 | Complete | Missing |
| 1892at7088 | Complete | Missing |
| 1893at7088 | Complete | Complete |
| 18at7088 | Duplicated | Missing |
| 1900at7088 | Complete | Missing |
| 1901at7088 | Complete | Missing |
| 1902at7088 | Complete | Missing |
| 1904at7088 | Complete | Missing |
| 1907at7088 | Fragmented | Missing |
| 190at7088 | Complete | Missing |
| 1910at7088 | Complete | Missing |
| 1911at7088 | Missing | Missing |
| 1913at7088 | Complete | Missing |
| 1918at7088 | Complete | Missing |
| 1922at7088 | Complete | Missing |
| 1924at7088 | Missing | Missing |
| 1927at7088 | Complete | Missing |
| 1928at7088 | Complete | Missing |
| 1929at7088 | Complete | Missing |
| 1931at7088 | Complete | Missing |
| 1932at7088 | Complete | Missing |
| 1934at7088 | Complete | Missing |
| 1935at7088 | Complete | Missing |
| 1936at7088 | Complete | Missing |
| 1938at7088 | Complete | Missing |
| 1939at7088 | Complete | Missing |
| 193at7088 | Complete | Missing |
| 1940at7088 | Complete | Missing |
| 1941at7088 | Complete | Missing |
| 1942at7088 | Complete | Missing |
| 1943at7088 | Complete | Fragmented |
| 1945at7088 | Complete | Missing |
| 1946at7088 | Complete | Missing |
| 1947at7088 | Complete | Missing |
| 1950at7088 | Duplicated | Missing |
| 1951at7088 | Complete | Missing |
| 1952at7088 | Complete | Complete |
| 1953at7088 | Complete | Missing |
| 1955at7088 | Complete | Missing |
| 1956at7088 | Complete | Missing |
| 1958at7088 | Duplicated | Missing |
| 1959at7088 | Complete | Missing |
| 1960at7088 | Complete | Missing |
| 1962at7088 | Complete | Missing |
| 1968at7088 | Complete | Missing |
| 196at7088 | Complete | Missing |
| 1970at7088 | Duplicated | Missing |
| 1974at7088 | Complete | Fragmented |
| 1982at7088 | Missing | Fragmented |
| 1984at7088 | Complete | Missing |
| 1985at7088 | Complete | Missing |
| 1987at7088 | Complete | Missing |
| 1990at7088 | Complete | Missing |
| 1992at7088 | Duplicated | Missing |
| 1993at7088 | Fragmented | Duplicated |
| 1994at7088 | Complete | Missing |
| 1999at7088 | Complete | Missing |
| 1at7088 | Complete | Missing |
| 2001at7088 | Complete | Missing |
| 2003at7088 | Complete | Missing |
| 200at7088 | Complete | Missing |
| 2015at7088 | Complete | Missing |
| 2019at7088 | Complete | Missing |
| 201at7088 | Missing | Missing |
| 2021at7088 | Complete | Missing |
| 2023at7088 | Complete | Missing |
| 2026at7088 | Complete | Missing |
| 2028at7088 | Complete | Missing |
| 2029at7088 | Missing | Missing |
| 2030at7088 | Complete | Missing |
| 2031at7088 | Complete | Missing |
| 2032at7088 | Duplicated | Missing |
| 2034at7088 | Complete | Missing |
| 2035at7088 | Complete | Missing |
| 2036at7088 | Complete | Complete |
| 2037at7088 | Complete | Complete |
| 2038at7088 | Complete | Missing |
| 2041at7088 | Complete | Missing |
| 204at7088 | Complete | Missing |
| 2051at7088 | Fragmented | Missing |
| 2052at7088 | Complete | Missing |
| 2054at7088 | Complete | Missing |
| 2055at7088 | Complete | Missing |
| 2056at7088 | Complete | Missing |
| 2060at7088 | Complete | Missing |
| 2067at7088 | Complete | Missing |
| 2068at7088 | Complete | Missing |
| 206at7088 | Complete | Missing |
| 2072at7088 | Complete | Missing |
| 2073at7088 | Complete | Missing |
| 2078at7088 | Complete | Missing |
| 2079at7088 | Complete | Missing |
| 207at7088 | Complete | Missing |
| 2081at7088 | Duplicated | Missing |
| 2084at7088 | Complete | Missing |
| 2086at7088 | Complete | Missing |
| 2087at7088 | Complete | Missing |
| 208at7088 | Complete | Missing |
| 2090at7088 | Duplicated | Missing |
| 2097at7088 | Complete | Missing |
| 2099at7088 | Complete | Missing |
| 20at7088 | Complete | Missing |
| 2101at7088 | Complete | Missing |
| 2103at7088 | Complete | Missing |
| 2109at7088 | Missing | Missing |
| 210at7088 | Complete | Missing |
| 2110at7088 | Complete | Missing |
| 2118at7088 | Complete | Missing |
| 211at7088 | Complete | Missing |
| 2121at7088 | Complete | Missing |
| 2122at7088 | Complete | Missing |
| 2124at7088 | Complete | Missing |
| 2126at7088 | Complete | Missing |
| 212at7088 | Fragmented | Missing |
| 2130at7088 | Complete | Missing |
| 2131at7088 | Complete | Missing |
| 2133at7088 | Complete | Missing |
| 2136at7088 | Complete | Missing |
| 2137at7088 | Complete | Missing |
| 2138at7088 | Complete | Missing |
| 2139at7088 | Complete | Missing |
| 2141at7088 | Complete | Missing |
| 2143at7088 | Complete | Missing |
| 2145at7088 | Complete | Missing |
| 2149at7088 | Complete | Missing |
| 2150at7088 | Complete | Missing |
| 2154at7088 | Duplicated | Complete |
| 2156at7088 | Complete | Missing |
| 2158at7088 | Complete | Missing |
| 215at7088 | Complete | Missing |
| 2160at7088 | Complete | Missing |
| 2163at7088 | Duplicated | Missing |
| 2164at7088 | Complete | Missing |
| 2166at7088 | Complete | Missing |
| 2169at7088 | Complete | Missing |
| 2173at7088 | Complete | Missing |
| 2174at7088 | Complete | Missing |
| 2177at7088 | Complete | Missing |
| 2178at7088 | Complete | Missing |
| 2181at7088 | Complete | Missing |
| 2182at7088 | Complete | Missing |
| 2183at7088 | Complete | Missing |
| 2185at7088 | Fragmented | Missing |
| 2188at7088 | Complete | Missing |
| 2190at7088 | Complete | Missing |
| 2191at7088 | Complete | Missing |
| 2194at7088 | Complete | Missing |
| 2201at7088 | Complete | Missing |
| 2208at7088 | Complete | Fragmented |
| 220at7088 | Complete | Missing |
| 2211at7088 | Complete | Missing |
| 2215at7088 | Duplicated | Missing |
| 2220at7088 | Complete | Missing |
| 2222at7088 | Fragmented | Missing |
| 2223at7088 | Missing | Missing |
| 2225at7088 | Complete | Missing |
| 2227at7088 | Complete | Missing |
| 2230at7088 | Complete | Missing |
| 2233at7088 | Complete | Missing |
| 2234at7088 | Complete | Missing |
| 2236at7088 | Complete | Missing |
| 2240at7088 | Complete | Missing |
| 2242at7088 | Complete | Complete |
| 2243at7088 | Complete | Missing |
| 2248at7088 | Complete | Missing |
| 2250at7088 | Missing | Missing |
| 2251at7088 | Complete | Missing |
| 2255at7088 | Complete | Missing |
| 2258at7088 | Complete | Missing |
| 2259at7088 | Complete | Missing |
| 2261at7088 | Complete | Missing |
| 2269at7088 | Complete | Missing |
| 2271at7088 | Complete | Missing |
| 2272at7088 | Complete | Missing |
| 2273at7088 | Missing | Missing |
| 2276at7088 | Complete | Missing |
| 2278at7088 | Complete | Missing |
| 2279at7088 | Complete | Missing |
| 2285at7088 | Complete | Missing |
| 2287at7088 | Complete | Missing |
| 2289at7088 | Complete | Missing |
| 2290at7088 | Complete | Missing |
| 2294at7088 | Complete | Missing |
| 2295at7088 | Complete | Fragmented |
| 2301at7088 | Complete | Missing |
| 2304at7088 | Complete | Missing |
| 2313at7088 | Complete | Missing |
| 2318at7088 | Complete | Missing |
| 231at7088 | Complete | Missing |
| 2321at7088 | Complete | Missing |
| 2322at7088 | Complete | Missing |
| 2324at7088 | Complete | Missing |
| 2325at7088 | Complete | Missing |
| 2327at7088 | Complete | Missing |
| 2329at7088 | Fragmented | Missing |
| 232at7088 | Complete | Missing |
| 2331at7088 | Complete | Missing |
| 2332at7088 | Complete | Missing |
| 2334at7088 | Fragmented | Missing |
| 2335at7088 | Complete | Complete |
| 2338at7088 | Complete | Missing |
| 2339at7088 | Complete | Missing |
| 2340at7088 | Complete | Missing |
| 2350at7088 | Complete | Fragmented |
| 2351at7088 | Duplicated | Missing |
| 2353at7088 | Complete | Fragmented |
| 2357at7088 | Duplicated | Missing |
| 2359at7088 | Missing | Missing |
| 235at7088 | Complete | Missing |
| 2362at7088 | Complete | Missing |
| 2363at7088 | Complete | Missing |
| 2366at7088 | Complete | Missing |
| 2367at7088 | Complete | Missing |
| 236at7088 | Complete | Missing |
| 2370at7088 | Complete | Missing |
| 2371at7088 | Duplicated | Missing |
| 2372at7088 | Complete | Missing |
| 2378at7088 | Duplicated | Fragmented |
| 2379at7088 | Complete | Missing |
| 2384at7088 | Complete | Missing |
| 2385at7088 | Complete | Missing |
| 2386at7088 | Complete | Missing |
| 2390at7088 | Complete | Missing |
| 2394at7088 | Complete | Missing |
| 2398at7088 | Duplicated | Missing |
| 2403at7088 | Complete | Missing |
| 2408at7088 | Complete | Missing |
| 2409at7088 | Missing | Missing |
| 2410at7088 | Complete | Fragmented |
| 2411at7088 | Complete | Missing |
| 2414at7088 | Complete | Missing |
| 2415at7088 | Complete | Missing |
| 2418at7088 | Complete | Missing |
| 2420at7088 | Complete | Missing |
| 2421at7088 | Complete | Missing |
| 2424at7088 | Complete | Missing |
| 2428at7088 | Complete | Missing |
| 242at7088 | Complete | Missing |
| 2432at7088 | Missing | Missing |
| 2434at7088 | Complete | Missing |
| 2435at7088 | Complete | Missing |
| 2440at7088 | Complete | Missing |
| 2442at7088 | Complete | Missing |
| 2447at7088 | Complete | Complete |
| 2448at7088 | Complete | Missing |
| 2449at7088 | Fragmented | Missing |
| 2451at7088 | Complete | Missing |
| 2452at7088 | Duplicated | Complete |
| 2453at7088 | Complete | Missing |
| 2454at7088 | Complete | Missing |
| 2455at7088 | Complete | Complete |
| 2456at7088 | Complete | Missing |
| 2459at7088 | Complete | Missing |
| 2460at7088 | Complete | Missing |
| 2461at7088 | Complete | Missing |
| 2465at7088 | Complete | Missing |
| 2469at7088 | Fragmented | Missing |
| 246at7088 | Complete | Missing |
| 2470at7088 | Complete | Missing |
| 2475at7088 | Complete | Missing |
| 2481at7088 | Duplicated | Missing |
| 2482at7088 | Complete | Missing |
| 2483at7088 | Duplicated | Missing |
| 2486at7088 | Duplicated | Missing |
| 248at7088 | Complete | Missing |
| 2490at7088 | Complete | Missing |
| 2496at7088 | Missing | Missing |
| 2497at7088 | Complete | Missing |
| 2498at7088 | Complete | Missing |
| 2501at7088 | Complete | Missing |
| 2504at7088 | Complete | Missing |
| 2507at7088 | Duplicated | Missing |
| 2509at7088 | Complete | Fragmented |
| 250at7088 | Complete | Missing |
| 2510at7088 | Complete | Missing |
| 2517at7088 | Complete | Missing |
| 2518at7088 | Complete | Missing |
| 2519at7088 | Complete | Missing |
| 251at7088 | Complete | Missing |
| 2522at7088 | Complete | Duplicated |
| 2523at7088 | Complete | Missing |
| 2527at7088 | Complete | Missing |
| 2528at7088 | Complete | Missing |
| 2529at7088 | Complete | Missing |
| 2530at7088 | Complete | Missing |
| 2535at7088 | Complete | Missing |
| 2543at7088 | Complete | Missing |
| 2545at7088 | Complete | Missing |
| 2546at7088 | Complete | Missing |
| 2547at7088 | Duplicated | Missing |
| 2548at7088 | Missing | Missing |
| 254at7088 | Complete | Missing |
| 2552at7088 | Complete | Missing |
| 2553at7088 | Complete | Missing |
| 2554at7088 | Complete | Missing |
| 2557at7088 | Complete | Missing |
| 2560at7088 | Complete | Missing |
| 2563at7088 | Complete | Missing |
| 2566at7088 | Missing | Missing |
| 2568at7088 | Complete | Missing |
| 2577at7088 | Missing | Missing |
| 2580at7088 | Complete | Fragmented |
| 2583at7088 | Complete | Missing |
| 2585at7088 | Missing | Missing |
| 2587at7088 | Complete | Missing |
| 2589at7088 | Complete | Missing |
| 258at7088 | Duplicated | Missing |
| 2594at7088 | Complete | Missing |
| 2596at7088 | Duplicated | Missing |
| 2599at7088 | Complete | Missing |
| 259at7088 | Complete | Missing |
| 25at7088 | Complete | Missing |
| 2600at7088 | Complete | Missing |
| 2603at7088 | Complete | Missing |
| 2608at7088 | Complete | Missing |
| 2610at7088 | Complete | Missing |
| 2614at7088 | Complete | Missing |
| 2615at7088 | Complete | Missing |
| 2618at7088 | Complete | Missing |
| 2621at7088 | Complete | Missing |
| 2624at7088 | Complete | Missing |
| 2629at7088 | Complete | Missing |
| 2633at7088 | Complete | Missing |
| 2635at7088 | Complete | Missing |
| 2637at7088 | Complete | Missing |
| 2639at7088 | Complete | Missing |
| 263at7088 | Complete | Missing |
| 2640at7088 | Fragmented | Missing |
| 2643at7088 | Complete | Missing |
| 2645at7088 | Complete | Missing |
| 2647at7088 | Duplicated | Missing |
| 2649at7088 | Complete | Missing |
| 2652at7088 | Complete | Missing |
| 2653at7088 | Complete | Missing |
| 2657at7088 | Complete | Missing |
| 2658at7088 | Complete | Missing |
| 2659at7088 | Duplicated | Missing |
| 2662at7088 | Complete | Missing |
| 2663at7088 | Complete | Missing |
| 2666at7088 | Complete | Missing |
| 2668at7088 | Complete | Missing |
| 266at7088 | Complete | Missing |
| 2671at7088 | Complete | Missing |
| 2672at7088 | Duplicated | Missing |
| 2679at7088 | Complete | Missing |
| 2686at7088 | Complete | Missing |
| 2687at7088 | Complete | Missing |
| 2690at7088 | Complete | Missing |
| 2694at7088 | Missing | Missing |
| 2696at7088 | Complete | Missing |
| 2699at7088 | Complete | Missing |
| 26at7088 | Complete | Missing |
| 2700at7088 | Complete | Missing |
| 2701at7088 | Complete | Missing |
| 2703at7088 | Complete | Missing |
| 2704at7088 | Complete | Missing |
| 270at7088 | Complete | Missing |
| 2710at7088 | Complete | Missing |
| 2714at7088 | Complete | Missing |
| 2715at7088 | Complete | Missing |
| 2718at7088 | Complete | Missing |
| 2719at7088 | Complete | Missing |
| 271at7088 | Complete | Missing |
| 2721at7088 | Complete | Missing |
| 2729at7088 | Complete | Missing |
| 2730at7088 | Complete | Missing |
| 2731at7088 | Complete | Missing |
| 2733at7088 | Complete | Missing |
| 2734at7088 | Complete | Missing |
| 2738at7088 | Complete | Missing |
| 273at7088 | Complete | Missing |
| 2740at7088 | Complete | Missing |
| 2743at7088 | Complete | Missing |
| 2744at7088 | Complete | Missing |
| 2746at7088 | Duplicated | Missing |
| 2747at7088 | Complete | Missing |
| 274at7088 | Complete | Missing |
| 2750at7088 | Complete | Missing |
| 2754at7088 | Complete | Missing |
| 2756at7088 | Complete | Missing |
| 2758at7088 | Missing | Missing |
| 2761at7088 | Complete | Missing |
| 2762at7088 | Duplicated | Missing |
| 2766at7088 | Duplicated | Missing |
| 2768at7088 | Complete | Missing |
| 2770at7088 | Complete | Missing |
| 2772at7088 | Complete | Fragmented |
| 2774at7088 | Duplicated | Missing |
| 2775at7088 | Complete | Missing |
| 2787at7088 | Complete | Missing |
| 2788at7088 | Complete | Missing |
| 278at7088 | Complete | Missing |
| 2791at7088 | Complete | Missing |
| 2796at7088 | Duplicated | Missing |
| 2797at7088 | Complete | Missing |
| 2798at7088 | Complete | Missing |
| 27at7088 | Complete | Missing |
| 2802at7088 | Complete | Missing |
| 2803at7088 | Missing | Missing |
| 2804at7088 | Complete | Missing |
| 2805at7088 | Complete | Missing |
| 2811at7088 | Complete | Missing |
| 2813at7088 | Duplicated | Missing |
| 2815at7088 | Complete | Missing |
| 2816at7088 | Complete | Missing |
| 2818at7088 | Complete | Missing |
| 2819at7088 | Duplicated | Missing |
| 281at7088 | Complete | Missing |
| 2821at7088 | Complete | Missing |
| 2826at7088 | Complete | Missing |
| 2829at7088 | Complete | Missing |
| 2830at7088 | Complete | Missing |
| 2833at7088 | Complete | Missing |
| 2834at7088 | Complete | Missing |
| 2835at7088 | Complete | Missing |
| 2842at7088 | Complete | Missing |
| 2845at7088 | Complete | Missing |
| 2848at7088 | Duplicated | Missing |
| 2855at7088 | Complete | Missing |
| 2857at7088 | Complete | Missing |
| 2859at7088 | Complete | Missing |
| 2860at7088 | Complete | Missing |
| 2864at7088 | Complete | Missing |
| 2873at7088 | Complete | Fragmented |
| 2877at7088 | Complete | Missing |
| 2884at7088 | Missing | Missing |
| 2885at7088 | Complete | Missing |
| 288at7088 | Complete | Missing |
| 2890at7088 | Missing | Missing |
| 2891at7088 | Complete | Missing |
| 2893at7088 | Complete | Missing |
| 2895at7088 | Complete | Missing |
| 28at7088 | Fragmented | Missing |
| 2902at7088 | Complete | Missing |
| 2905at7088 | Complete | Missing |
| 2906at7088 | Complete | Missing |
| 2910at7088 | Complete | Missing |
| 2912at7088 | Complete | Missing |
| 2913at7088 | Complete | Missing |
| 2917at7088 | Complete | Missing |
| 2918at7088 | Complete | Missing |
| 2926at7088 | Complete | Missing |
| 2928at7088 | Complete | Missing |
| 2930at7088 | Fragmented | Missing |
| 2931at7088 | Complete | Missing |
| 2932at7088 | Complete | Missing |
| 2934at7088 | Missing | Missing |
| 2935at7088 | Complete | Fragmented |
| 2936at7088 | Complete | Missing |
| 2938at7088 | Complete | Missing |
| 2939at7088 | Complete | Complete |
| 2940at7088 | Complete | Missing |
| 2942at7088 | Complete | Missing |
| 2943at7088 | Complete | Fragmented |
| 2945at7088 | Complete | Missing |
| 2948at7088 | Complete | Missing |
| 2949at7088 | Complete | Missing |
| 2952at7088 | Missing | Missing |
| 2953at7088 | Complete | Missing |
| 2959at7088 | Complete | Missing |
| 2965at7088 | Complete | Missing |
| 2966at7088 | Complete | Missing |
| 296at7088 | Complete | Missing |
| 2972at7088 | Complete | Missing |
| 2976at7088 | Complete | Missing |
| 2978at7088 | Missing | Missing |
| 2979at7088 | Complete | Missing |
| 2980at7088 | Complete | Missing |
| 2981at7088 | Complete | Missing |
| 2982at7088 | Duplicated | Missing |
| 2984at7088 | Complete | Complete |
| 2987at7088 | Complete | Missing |
| 2990at7088 | Complete | Missing |
| 2991at7088 | Complete | Missing |
| 2994at7088 | Complete | Missing |
| 2995at7088 | Complete | Missing |
| 2998at7088 | Complete | Missing |
| 2999at7088 | Complete | Missing |
| 2at7088 | Complete | Missing |
| 3000at7088 | Complete | Fragmented |
| 3003at7088 | Complete | Missing |
| 3004at7088 | Complete | Fragmented |
| 3005at7088 | Complete | Missing |
| 3006at7088 | Complete | Missing |
| 3010at7088 | Complete | Missing |
| 3012at7088 | Missing | Duplicated |
| 3016at7088 | Duplicated | Missing |
| 3018at7088 | Complete | Missing |
| 3021at7088 | Complete | Missing |
| 3023at7088 | Missing | Missing |
| 3025at7088 | Complete | Missing |
| 3026at7088 | Complete | Missing |
| 3027at7088 | Complete | Missing |
| 3029at7088 | Complete | Missing |
| 302at7088 | Complete | Missing |
| 3030at7088 | Complete | Missing |
| 3032at7088 | Complete | Missing |
| 303at7088 | Complete | Missing |
| 3040at7088 | Complete | Missing |
| 3042at7088 | Complete | Missing |
| 3045at7088 | Complete | Missing |
| 3047at7088 | Fragmented | Missing |
| 3048at7088 | Complete | Complete |
| 3051at7088 | Complete | Missing |
| 3053at7088 | Duplicated | Complete |
| 3055at7088 | Complete | Missing |
| 3057at7088 | Complete | Missing |
| 3059at7088 | Complete | Missing |
| 3065at7088 | Complete | Missing |
| 3066at7088 | Complete | Missing |
| 3067at7088 | Complete | Missing |
| 3069at7088 | Duplicated | Missing |
| 3071at7088 | Complete | Missing |
| 3074at7088 | Complete | Missing |
| 3075at7088 | Complete | Missing |
| 3078at7088 | Complete | Missing |
| 3079at7088 | Complete | Missing |
| 307at7088 | Complete | Missing |
| 3080at7088 | Complete | Missing |
| 3084at7088 | Complete | Missing |
| 3086at7088 | Complete | Missing |
| 3093at7088 | Complete | Missing |
| 3094at7088 | Complete | Missing |
| 3104at7088 | Complete | Missing |
| 3105at7088 | Complete | Missing |
| 3107at7088 | Duplicated | Missing |
| 3110at7088 | Complete | Missing |
| 3114at7088 | Complete | Missing |
| 3115at7088 | Complete | Missing |
| 3118at7088 | Complete | Missing |
| 311at7088 | Complete | Fragmented |
| 3122at7088 | Complete | Missing |
| 3123at7088 | Complete | Missing |
| 3125at7088 | Complete | Missing |
| 3126at7088 | Complete | Missing |
| 3127at7088 | Complete | Missing |
| 3128at7088 | Complete | Missing |
| 3129at7088 | Complete | Missing |
| 3131at7088 | Complete | Missing |
| 3132at7088 | Complete | Missing |
| 3133at7088 | Complete | Missing |
| 3135at7088 | Complete | Missing |
| 3137at7088 | Duplicated | Missing |
| 3144at7088 | Complete | Missing |
| 3145at7088 | Complete | Missing |
| 3152at7088 | Complete | Missing |
| 3153at7088 | Complete | Missing |
| 3155at7088 | Complete | Missing |
| 3159at7088 | Complete | Missing |
| 315at7088 | Complete | Missing |
| 3160at7088 | Complete | Missing |
| 3161at7088 | Complete | Missing |
| 3162at7088 | Fragmented | Missing |
| 3163at7088 | Complete | Missing |
| 3165at7088 | Complete | Missing |
| 3166at7088 | Missing | Missing |
| 3167at7088 | Complete | Missing |
| 316at7088 | Complete | Missing |
| 3174at7088 | Complete | Missing |
| 3175at7088 | Complete | Missing |
| 3178at7088 | Complete | Missing |
| 3180at7088 | Missing | Fragmented |
| 3183at7088 | Complete | Missing |
| 3186at7088 | Duplicated | Missing |
| 3187at7088 | Complete | Missing |
| 3188at7088 | Complete | Missing |
| 318at7088 | Complete | Missing |
| 3195at7088 | Complete | Missing |
| 3197at7088 | Complete | Missing |
| 3198at7088 | Complete | Missing |
| 31at7088 | Complete | Missing |
| 3200at7088 | Duplicated | Missing |
| 3204at7088 | Complete | Missing |
| 3207at7088 | Complete | Missing |
| 3209at7088 | Complete | Missing |
| 3211at7088 | Complete | Missing |
| 3212at7088 | Missing | Missing |
| 3216at7088 | Complete | Missing |
| 3218at7088 | Complete | Missing |
| 3219at7088 | Complete | Missing |
| 321at7088 | Fragmented | Missing |
| 3220at7088 | Duplicated | Missing |
| 3221at7088 | Complete | Missing |
| 3224at7088 | Complete | Missing |
| 3225at7088 | Complete | Missing |
| 3226at7088 | Complete | Missing |
| 3228at7088 | Complete | Missing |
| 322at7088 | Missing | Missing |
| 3231at7088 | Missing | Missing |
| 3234at7088 | Complete | Missing |
| 3237at7088 | Missing | Missing |
| 3241at7088 | Complete | Missing |
| 3242at7088 | Complete | Missing |
| 3245at7088 | Complete | Missing |
| 3248at7088 | Complete | Fragmented |
| 324at7088 | Complete | Missing |
| 3250at7088 | Complete | Missing |
| 3251at7088 | Complete | Missing |
| 3253at7088 | Complete | Complete |
| 3254at7088 | Complete | Missing |
| 3255at7088 | Complete | Missing |
| 3257at7088 | Complete | Fragmented |
| 3258at7088 | Complete | Missing |
| 3259at7088 | Complete | Missing |
| 325at7088 | Missing | Missing |
| 3265at7088 | Complete | Missing |
| 3269at7088 | Complete | Missing |
| 326at7088 | Complete | Missing |
| 3272at7088 | Complete | Missing |
| 3273at7088 | Complete | Missing |
| 3275at7088 | Complete | Missing |
| 3276at7088 | Complete | Missing |
| 3277at7088 | Complete | Missing |
| 3278at7088 | Complete | Missing |
| 3279at7088 | Complete | Missing |
| 327at7088 | Complete | Fragmented |
| 3284at7088 | Complete | Missing |
| 3287at7088 | Complete | Missing |
| 328at7088 | Fragmented | Missing |
| 3290at7088 | Complete | Missing |
| 3293at7088 | Complete | Missing |
| 3297at7088 | Complete | Missing |
| 3298at7088 | Complete | Missing |
| 3306at7088 | Complete | Missing |
| 3307at7088 | Complete | Missing |
| 3312at7088 | Complete | Missing |
| 3313at7088 | Complete | Missing |
| 3314at7088 | Complete | Complete |
| 3315at7088 | Complete | Missing |
| 3323at7088 | Complete | Missing |
| 3324at7088 | Complete | Missing |
| 3326at7088 | Complete | Missing |
| 3327at7088 | Complete | Missing |
| 332at7088 | Complete | Missing |
| 3330at7088 | Complete | Missing |
| 3331at7088 | Complete | Missing |
| 3332at7088 | Complete | Missing |
| 3338at7088 | Complete | Fragmented |
| 3339at7088 | Complete | Missing |
| 3343at7088 | Complete | Missing |
| 3345at7088 | Complete | Missing |
| 3348at7088 | Complete | Missing |
| 3351at7088 | Complete | Missing |
| 3352at7088 | Fragmented | Missing |
| 3353at7088 | Complete | Missing |
| 3356at7088 | Complete | Missing |
| 3358at7088 | Complete | Missing |
| 3359at7088 | Complete | Missing |
| 335at7088 | Complete | Missing |
| 3361at7088 | Complete | Missing |
| 3362at7088 | Complete | Missing |
| 3363at7088 | Duplicated | Missing |
| 3364at7088 | Complete | Fragmented |
| 3365at7088 | Complete | Missing |
| 3366at7088 | Complete | Missing |
| 3368at7088 | Complete | Missing |
| 3372at7088 | Complete | Missing |
| 3373at7088 | Complete | Missing |
| 3379at7088 | Complete | Missing |
| 3381at7088 | Fragmented | Missing |
| 3383at7088 | Complete | Missing |
| 3384at7088 | Complete | Missing |
| 3387at7088 | Complete | Missing |
| 3388at7088 | Complete | Missing |
| 3389at7088 | Complete | Missing |
| 338at7088 | Complete | Missing |
| 3390at7088 | Complete | Missing |
| 3393at7088 | Complete | Missing |
| 3396at7088 | Complete | Missing |
| 33at7088 | Complete | Missing |
| 3400at7088 | Complete | Fragmented |
| 3401at7088 | Complete | Missing |
| 3402at7088 | Missing | Missing |
| 3404at7088 | Complete | Missing |
| 3409at7088 | Complete | Missing |
| 340at7088 | Complete | Missing |
| 3410at7088 | Complete | Missing |
| 3415at7088 | Complete | Missing |
| 3416at7088 | Complete | Missing |
| 3417at7088 | Complete | Missing |
| 3419at7088 | Complete | Missing |
| 3424at7088 | Complete | Complete |
| 342at7088 | Complete | Missing |
| 3430at7088 | Complete | Missing |
| 3431at7088 | Complete | Missing |
| 3433at7088 | Complete | Missing |
| 3435at7088 | Complete | Missing |
| 3442at7088 | Duplicated | Missing |
| 3443at7088 | Missing | Missing |
| 3447at7088 | Complete | Missing |
| 3451at7088 | Complete | Missing |
| 3453at7088 | Complete | Missing |
| 345at7088 | Complete | Missing |
| 3460at7088 | Complete | Missing |
| 3468at7088 | Complete | Missing |
| 3471at7088 | Complete | Missing |
| 3473at7088 | Complete | Missing |
| 3474at7088 | Complete | Missing |
| 3477at7088 | Complete | Missing |
| 3478at7088 | Complete | Duplicated |
| 3479at7088 | Complete | Missing |
| 3480at7088 | Complete | Missing |
| 3485at7088 | Complete | Missing |
| 3486at7088 | Complete | Missing |
| 3489at7088 | Complete | Missing |
| 3491at7088 | Missing | Missing |
| 3493at7088 | Complete | Missing |
| 3494at7088 | Complete | Missing |
| 3496at7088 | Missing | Missing |
| 3497at7088 | Complete | Missing |
| 34at7088 | Complete | Fragmented |
| 3503at7088 | Complete | Missing |
| 3504at7088 | Complete | Missing |
| 3507at7088 | Duplicated | Missing |
| 3508at7088 | Complete | Missing |
| 350at7088 | Complete | Missing |
| 3510at7088 | Duplicated | Missing |
| 3514at7088 | Complete | Missing |
| 3516at7088 | Complete | Missing |
| 3517at7088 | Complete | Missing |
| 3519at7088 | Complete | Missing |
| 3520at7088 | Complete | Missing |
| 3521at7088 | Complete | Missing |
| 3524at7088 | Complete | Missing |
| 3526at7088 | Complete | Missing |
| 3528at7088 | Duplicated | Missing |
| 3529at7088 | Complete | Fragmented |
| 3537at7088 | Complete | Missing |
| 3539at7088 | Complete | Missing |
| 353at7088 | Complete | Fragmented |
| 3541at7088 | Complete | Missing |
| 3542at7088 | Complete | Missing |
| 3548at7088 | Complete | Missing |
| 3549at7088 | Complete | Missing |
| 354at7088 | Complete | Missing |
| 3551at7088 | Complete | Missing |
| 3556at7088 | Complete | Missing |
| 3558at7088 | Complete | Missing |
| 355at7088 | Complete | Missing |
| 3563at7088 | Complete | Missing |
| 3564at7088 | Complete | Missing |
| 3567at7088 | Complete | Missing |
| 356at7088 | Complete | Missing |
| 3571at7088 | Complete | Missing |
| 3577at7088 | Complete | Missing |
| 357at7088 | Complete | Missing |
| 3580at7088 | Missing | Missing |
| 3584at7088 | Complete | Missing |
| 3587at7088 | Complete | Missing |
| 3589at7088 | Complete | Missing |
| 3592at7088 | Complete | Missing |
| 3594at7088 | Complete | Missing |
| 3595at7088 | Complete | Missing |
| 3596at7088 | Complete | Missing |
| 3597at7088 | Complete | Missing |
| 3598at7088 | Complete | Missing |
| 3599at7088 | Duplicated | Fragmented |
| 3600at7088 | Missing | Duplicated |
| 3602at7088 | Duplicated | Missing |
| 3604at7088 | Complete | Missing |
| 3607at7088 | Missing | Missing |
| 3608at7088 | Duplicated | Missing |
| 360at7088 | Complete | Missing |
| 3611at7088 | Duplicated | Missing |
| 3616at7088 | Complete | Missing |
| 3618at7088 | Complete | Missing |
| 3631at7088 | Complete | Missing |
| 3633at7088 | Complete | Missing |
| 3634at7088 | Complete | Missing |
| 363at7088 | Complete | Missing |
| 3641at7088 | Complete | Missing |
| 3648at7088 | Complete | Missing |
| 3653at7088 | Complete | Missing |
| 3656at7088 | Complete | Missing |
| 3658at7088 | Complete | Missing |
| 3659at7088 | Complete | Missing |
| 3660at7088 | Complete | Missing |
| 3662at7088 | Complete | Missing |
| 3663at7088 | Complete | Missing |
| 3664at7088 | Complete | Complete |
| 3665at7088 | Complete | Complete |
| 3666at7088 | Complete | Missing |
| 3667at7088 | Complete | Missing |
| 3668at7088 | Complete | Missing |
| 366at7088 | Complete | Missing |
| 3671at7088 | Complete | Missing |
| 3672at7088 | Complete | Missing |
| 3673at7088 | Complete | Complete |
| 3675at7088 | Complete | Missing |
| 3678at7088 | Complete | Missing |
| 367at7088 | Complete | Missing |
| 3680at7088 | Complete | Missing |
| 3681at7088 | Complete | Missing |
| 3683at7088 | Duplicated | Missing |
| 3684at7088 | Complete | Missing |
| 3686at7088 | Complete | Missing |
| 3689at7088 | Complete | Missing |
| 3695at7088 | Complete | Missing |
| 36at7088 | Complete | Missing |
| 3709at7088 | Complete | Missing |
| 3718at7088 | Complete | Missing |
| 3722at7088 | Complete | Missing |
| 3725at7088 | Complete | Missing |
| 3726at7088 | Complete | Missing |
| 3727at7088 | Complete | Missing |
| 3730at7088 | Duplicated | Missing |
| 3735at7088 | Complete | Missing |
| 3742at7088 | Complete | Missing |
| 3743at7088 | Complete | Missing |
| 3745at7088 | Complete | Missing |
| 3749at7088 | Complete | Missing |
| 3753at7088 | Duplicated | Missing |
| 3756at7088 | Complete | Missing |
| 375at7088 | Complete | Missing |
| 3760at7088 | Fragmented | Missing |
| 3767at7088 | Complete | Missing |
| 3773at7088 | Complete | Missing |
| 3774at7088 | Complete | Missing |
| 3775at7088 | Complete | Missing |
| 3779at7088 | Complete | Missing |
| 3780at7088 | Complete | Missing |
| 3785at7088 | Complete | Missing |
| 3791at7088 | Complete | Missing |
| 3793at7088 | Duplicated | Fragmented |
| 3794at7088 | Complete | Missing |
| 3795at7088 | Complete | Missing |
| 3799at7088 | Complete | Missing |
| 37at7088 | Complete | Missing |
| 3800at7088 | Complete | Missing |
| 3802at7088 | Complete | Missing |
| 3804at7088 | Complete | Missing |
| 3807at7088 | Complete | Missing |
| 380at7088 | Complete | Missing |
| 3811at7088 | Complete | Missing |
| 3817at7088 | Complete | Missing |
| 3818at7088 | Complete | Missing |
| 3821at7088 | Complete | Missing |
| 3823at7088 | Complete | Missing |
| 3825at7088 | Missing | Missing |
| 3827at7088 | Complete | Missing |
| 3829at7088 | Complete | Missing |
| 382at7088 | Missing | Missing |
| 3830at7088 | Complete | Missing |
| 3833at7088 | Complete | Missing |
| 3834at7088 | Complete | Missing |
| 3839at7088 | Complete | Missing |
| 383at7088 | Complete | Missing |
| 3840at7088 | Complete | Missing |
| 3842at7088 | Complete | Missing |
| 3845at7088 | Complete | Missing |
| 384at7088 | Complete | Missing |
| 3853at7088 | Complete | Missing |
| 3855at7088 | Complete | Missing |
| 3859at7088 | Complete | Missing |
| 385at7088 | Complete | Missing |
| 3860at7088 | Complete | Missing |
| 3864at7088 | Complete | Missing |
| 3865at7088 | Complete | Missing |
| 3866at7088 | Complete | Missing |
| 3869at7088 | Complete | Missing |
| 386at7088 | Complete | Missing |
| 3870at7088 | Complete | Missing |
| 3872at7088 | Complete | Missing |
| 3873at7088 | Complete | Missing |
| 3874at7088 | Complete | Missing |
| 3876at7088 | Complete | Missing |
| 3879at7088 | Complete | Missing |
| 3882at7088 | Duplicated | Missing |
| 3885at7088 | Complete | Missing |
| 3887at7088 | Complete | Missing |
| 3888at7088 | Missing | Missing |
| 3889at7088 | Complete | Missing |
| 3890at7088 | Complete | Missing |
| 3898at7088 | Missing | Missing |
| 3899at7088 | Duplicated | Missing |
| 389at7088 | Complete | Missing |
| 38at7088 | Complete | Missing |
| 3904at7088 | Complete | Missing |
| 3905at7088 | Fragmented | Fragmented |
| 3908at7088 | Complete | Missing |
| 3911at7088 | Complete | Missing |
| 3912at7088 | Complete | Missing |
| 3915at7088 | Complete | Missing |
| 3916at7088 | Complete | Missing |
| 3919at7088 | Complete | Missing |
| 391at7088 | Complete | Missing |
| 3921at7088 | Complete | Missing |
| 3923at7088 | Complete | Missing |
| 3924at7088 | Complete | Missing |
| 3925at7088 | Duplicated | Missing |
| 3926at7088 | Complete | Missing |
| 3929at7088 | Complete | Missing |
| 392at7088 | Complete | Missing |
| 3931at7088 | Complete | Missing |
| 3938at7088 | Complete | Missing |
| 3939at7088 | Complete | Missing |
| 393at7088 | Complete | Missing |
| 3946at7088 | Complete | Missing |
| 3947at7088 | Duplicated | Missing |
| 3949at7088 | Complete | Missing |
| 394at7088 | Complete | Missing |
| 3955at7088 | Complete | Missing |
| 3956at7088 | Duplicated | Missing |
| 3963at7088 | Complete | Missing |
| 3964at7088 | Complete | Missing |
| 3966at7088 | Complete | Missing |
| 3969at7088 | Missing | Missing |
| 396at7088 | Complete | Missing |
| 3973at7088 | Complete | Missing |
| 3975at7088 | Complete | Missing |
| 3982at7088 | Complete | Missing |
| 3985at7088 | Complete | Missing |
| 3989at7088 | Duplicated | Missing |
| 3990at7088 | Complete | Missing |
| 3994at7088 | Duplicated | Complete |
| 3997at7088 | Complete | Missing |
| 3998at7088 | Complete | Missing |
| 3at7088 | Complete | Missing |
| 4002at7088 | Complete | Missing |
| 4008at7088 | Complete | Missing |
| 4009at7088 | Complete | Missing |
| 4010at7088 | Duplicated | Missing |
| 4011at7088 | Complete | Missing |
| 4013at7088 | Duplicated | Missing |
| 4017at7088 | Complete | Missing |
| 401at7088 | Complete | Missing |
| 4020at7088 | Duplicated | Missing |
| 4021at7088 | Complete | Missing |
| 4024at7088 | Complete | Fragmented |
| 4025at7088 | Complete | Missing |
| 4027at7088 | Complete | Missing |
| 4029at7088 | Complete | Missing |
| 4030at7088 | Complete | Missing |
| 4031at7088 | Complete | Missing |
| 4032at7088 | Complete | Complete |
| 4036at7088 | Missing | Missing |
| 4037at7088 | Duplicated | Missing |
| 4041at7088 | Complete | Missing |
| 4043at7088 | Complete | Missing |
| 4046at7088 | Duplicated | Missing |
| 4052at7088 | Complete | Missing |
| 4059at7088 | Complete | Missing |
| 4063at7088 | Duplicated | Missing |
| 4064at7088 | Complete | Missing |
| 4066at7088 | Complete | Missing |
| 4067at7088 | Fragmented | Missing |
| 4068at7088 | Complete | Missing |
| 4069at7088 | Complete | Missing |
| 4070at7088 | Missing | Missing |
| 4073at7088 | Complete | Missing |
| 4077at7088 | Complete | Missing |
| 4078at7088 | Complete | Missing |
| 4079at7088 | Complete | Missing |
| 4081at7088 | Complete | Missing |
| 4085at7088 | Complete | Missing |
| 4089at7088 | Complete | Missing |
| 408at7088 | Complete | Missing |
| 4090at7088 | Complete | Missing |
| 4092at7088 | Complete | Missing |
| 4093at7088 | Complete | Missing |
| 4100at7088 | Complete | Missing |
| 4108at7088 | Complete | Missing |
| 4109at7088 | Complete | Complete |
| 4110at7088 | Missing | Missing |
| 4114at7088 | Duplicated | Missing |
| 4118at7088 | Complete | Missing |
| 4119at7088 | Complete | Missing |
| 4121at7088 | Complete | Missing |
| 4123at7088 | Complete | Missing |
| 412at7088 | Complete | Missing |
| 4130at7088 | Complete | Missing |
| 4131at7088 | Complete | Fragmented |
| 4132at7088 | Complete | Missing |
| 4141at7088 | Complete | Missing |
| 4142at7088 | Complete | Missing |
| 4144at7088 | Complete | Missing |
| 4147at7088 | Complete | Missing |
| 4150at7088 | Complete | Missing |
| 4153at7088 | Complete | Missing |
| 4162at7088 | Duplicated | Missing |
| 4163at7088 | Complete | Missing |
| 4165at7088 | Complete | Missing |
| 4167at7088 | Complete | Missing |
| 4172at7088 | Missing | Missing |
| 4173at7088 | Complete | Complete |
| 4174at7088 | Complete | Fragmented |
| 4175at7088 | Complete | Missing |
| 4176at7088 | Complete | Missing |
| 4178at7088 | Complete | Missing |
| 417at7088 | Complete | Missing |
| 4180at7088 | Complete | Missing |
| 4181at7088 | Complete | Missing |
| 4182at7088 | Complete | Missing |
| 4185at7088 | Complete | Missing |
| 4189at7088 | Complete | Missing |
| 4190at7088 | Complete | Missing |
| 4197at7088 | Duplicated | Missing |
| 4198at7088 | Complete | Missing |
| 4199at7088 | Complete | Missing |
| 41at7088 | Complete | Missing |
| 4200at7088 | Missing | Missing |
| 4202at7088 | Complete | Missing |
| 4204at7088 | Complete | Missing |
| 4208at7088 | Complete | Missing |
| 420at7088 | Complete | Missing |
| 4212at7088 | Missing | Missing |
| 4214at7088 | Duplicated | Missing |
| 4219at7088 | Complete | Missing |
| 4223at7088 | Complete | Missing |
| 4226at7088 | Complete | Missing |
| 4227at7088 | Complete | Missing |
| 4229at7088 | Complete | Missing |
| 4235at7088 | Complete | Missing |
| 4237at7088 | Complete | Missing |
| 4238at7088 | Missing | Missing |
| 4241at7088 | Duplicated | Missing |
| 4242at7088 | Duplicated | Missing |
| 4243at7088 | Complete | Missing |
| 4244at7088 | Complete | Missing |
| 4247at7088 | Complete | Duplicated |
| 4251at7088 | Complete | Missing |
| 4253at7088 | Complete | Missing |
| 4256at7088 | Complete | Missing |
| 4260at7088 | Complete | Missing |
| 4263at7088 | Complete | Missing |
| 4264at7088 | Complete | Missing |
| 4266at7088 | Fragmented | Missing |
| 4267at7088 | Complete | Missing |
| 4268at7088 | Complete | Missing |
| 4269at7088 | Complete | Missing |
| 4270at7088 | Complete | Missing |
| 4271at7088 | Complete | Missing |
| 4272at7088 | Complete | Missing |
| 4275at7088 | Missing | Missing |
| 4279at7088 | Complete | Missing |
| 427at7088 | Complete | Missing |
| 4286at7088 | Complete | Missing |
| 4287at7088 | Missing | Missing |
| 4288at7088 | Complete | Missing |
| 4297at7088 | Complete | Missing |
| 4298at7088 | Complete | Missing |
| 429at7088 | Complete | Missing |
| 4300at7088 | Complete | Missing |
| 4301at7088 | Complete | Missing |
| 4304at7088 | Duplicated | Missing |
| 4305at7088 | Complete | Missing |
| 4306at7088 | Complete | Missing |
| 4307at7088 | Complete | Missing |
| 4308at7088 | Complete | Missing |
| 430at7088 | Complete | Missing |
| 4315at7088 | Fragmented | Missing |
| 4317at7088 | Complete | Missing |
| 4318at7088 | Complete | Missing |
| 4320at7088 | Complete | Missing |
| 4322at7088 | Complete | Missing |
| 4323at7088 | Complete | Missing |
| 4325at7088 | Missing | Missing |
| 4331at7088 | Complete | Fragmented |
| 4333at7088 | Complete | Missing |
| 4335at7088 | Complete | Missing |
| 4346at7088 | Complete | Missing |
| 4348at7088 | Complete | Missing |
| 434at7088 | Complete | Missing |
| 4350at7088 | Duplicated | Missing |
| 4352at7088 | Complete | Missing |
| 4354at7088 | Missing | Missing |
| 4360at7088 | Complete | Missing |
| 4362at7088 | Missing | Fragmented |
| 4364at7088 | Complete | Missing |
| 4368at7088 | Complete | Missing |
| 4369at7088 | Complete | Missing |
| 4370at7088 | Complete | Missing |
| 4371at7088 | Complete | Missing |
| 4377at7088 | Missing | Missing |
| 4379at7088 | Complete | Missing |
| 437at7088 | Complete | Missing |
| 4383at7088 | Complete | Missing |
| 4392at7088 | Complete | Missing |
| 4398at7088 | Complete | Missing |
| 43at7088 | Complete | Missing |
| 4400at7088 | Complete | Missing |
| 4401at7088 | Complete | Missing |
| 4402at7088 | Complete | Missing |
| 4403at7088 | Complete | Missing |
| 4405at7088 | Complete | Missing |
| 4406at7088 | Complete | Missing |
| 4412at7088 | Duplicated | Missing |
| 4414at7088 | Complete | Missing |
| 4415at7088 | Complete | Missing |
| 4416at7088 | Complete | Missing |
| 4417at7088 | Complete | Missing |
| 4418at7088 | Complete | Missing |
| 4420at7088 | Complete | Missing |
| 4422at7088 | Missing | Missing |
| 4423at7088 | Complete | Missing |
| 4425at7088 | Complete | Missing |
| 4427at7088 | Fragmented | Missing |
| 4434at7088 | Complete | Missing |
| 4439at7088 | Complete | Missing |
| 443at7088 | Complete | Missing |
| 4440at7088 | Complete | Missing |
| 4443at7088 | Complete | Missing |
| 4449at7088 | Complete | Missing |
| 4451at7088 | Complete | Missing |
| 4454at7088 | Duplicated | Missing |
| 4455at7088 | Complete | Missing |
| 4457at7088 | Complete | Missing |
| 445at7088 | Complete | Missing |
| 4462at7088 | Complete | Missing |
| 4469at7088 | Complete | Missing |
| 4471at7088 | Complete | Missing |
| 4473at7088 | Complete | Fragmented |
| 4477at7088 | Complete | Missing |
| 4479at7088 | Complete | Missing |
| 4480at7088 | Complete | Missing |
| 4481at7088 | Duplicated | Missing |
| 4483at7088 | Complete | Missing |
| 4485at7088 | Complete | Missing |
| 4486at7088 | Complete | Missing |
| 4487at7088 | Complete | Missing |
| 4492at7088 | Complete | Missing |
| 4493at7088 | Complete | Complete |
| 4497at7088 | Complete | Missing |
| 4499at7088 | Complete | Missing |
| 4500at7088 | Complete | Missing |
| 4502at7088 | Complete | Missing |
| 4504at7088 | Complete | Missing |
| 450at7088 | Missing | Missing |
| 4524at7088 | Complete | Missing |
| 4527at7088 | Complete | Missing |
| 4528at7088 | Complete | Missing |
| 4533at7088 | Complete | Missing |
| 4539at7088 | Complete | Missing |
| 4541at7088 | Complete | Missing |
| 4545at7088 | Complete | Missing |
| 4546at7088 | Complete | Missing |
| 4549at7088 | Complete | Missing |
| 4554at7088 | Complete | Missing |
| 4555at7088 | Complete | Missing |
| 4557at7088 | Complete | Missing |
| 455at7088 | Complete | Complete |
| 4560at7088 | Complete | Missing |
| 456at7088 | Complete | Missing |
| 4576at7088 | Complete | Missing |
| 4579at7088 | Complete | Missing |
| 458at7088 | Complete | Missing |
| 4595at7088 | Complete | Missing |
| 4597at7088 | Duplicated | Missing |
| 4602at7088 | Complete | Missing |
| 4605at7088 | Missing | Missing |
| 4608at7088 | Complete | Missing |
| 4609at7088 | Complete | Missing |
| 4610at7088 | Complete | Missing |
| 4616at7088 | Complete | Missing |
| 461at7088 | Complete | Missing |
| 4621at7088 | Complete | Fragmented |
| 4623at7088 | Complete | Missing |
| 4624at7088 | Complete | Missing |
| 4625at7088 | Duplicated | Missing |
| 4626at7088 | Complete | Missing |
| 4627at7088 | Complete | Missing |
| 4630at7088 | Complete | Missing |
| 4631at7088 | Complete | Missing |
| 4632at7088 | Complete | Missing |
| 4633at7088 | Complete | Missing |
| 4639at7088 | Complete | Missing |
| 4645at7088 | Complete | Missing |
| 4649at7088 | Complete | Complete |
| 464at7088 | Complete | Missing |
| 4650at7088 | Complete | Missing |
| 4653at7088 | Complete | Missing |
| 4654at7088 | Complete | Missing |
| 4655at7088 | Complete | Missing |
| 4656at7088 | Complete | Missing |
| 4657at7088 | Missing | Missing |
| 4663at7088 | Duplicated | Missing |
| 4664at7088 | Complete | Missing |
| 4668at7088 | Complete | Missing |
| 4670at7088 | Complete | Missing |
| 4671at7088 | Complete | Missing |
| 4674at7088 | Complete | Missing |
| 4677at7088 | Complete | Missing |
| 467at7088 | Missing | Fragmented |
| 4680at7088 | Complete | Missing |
| 4681at7088 | Complete | Missing |
| 4685at7088 | Complete | Missing |
| 4688at7088 | Complete | Missing |
| 4689at7088 | Complete | Missing |
| 468at7088 | Complete | Missing |
| 4690at7088 | Complete | Missing |
| 4692at7088 | Complete | Missing |
| 4694at7088 | Complete | Missing |
| 4698at7088 | Complete | Missing |
| 46at7088 | Complete | Fragmented |
| 4707at7088 | Complete | Missing |
| 4709at7088 | Complete | Missing |
| 4710at7088 | Complete | Missing |
| 4718at7088 | Complete | Missing |
| 4719at7088 | Complete | Missing |
| 4723at7088 | Complete | Missing |
| 4724at7088 | Complete | Missing |
| 4726at7088 | Complete | Missing |
| 4729at7088 | Complete | Missing |
| 4731at7088 | Missing | Missing |
| 4735at7088 | Complete | Missing |
| 4739at7088 | Complete | Missing |
| 4740at7088 | Complete | Missing |
| 4742at7088 | Duplicated | Missing |
| 4744at7088 | Complete | Missing |
| 4746at7088 | Complete | Missing |
| 4748at7088 | Complete | Missing |
| 4749at7088 | Complete | Missing |
| 4750at7088 | Complete | Missing |
| 4752at7088 | Complete | Missing |
| 4753at7088 | Complete | Complete |
| 4760at7088 | Complete | Missing |
| 4761at7088 | Complete | Fragmented |
| 4764at7088 | Duplicated | Missing |
| 4768at7088 | Complete | Missing |
| 4776at7088 | Missing | Missing |
| 4778at7088 | Duplicated | Missing |
| 4779at7088 | Fragmented | Missing |
| 4782at7088 | Complete | Complete |
| 4784at7088 | Complete | Missing |
| 4789at7088 | Complete | Missing |
| 478at7088 | Complete | Missing |
| 4790at7088 | Complete | Missing |
| 4795at7088 | Complete | Missing |
| 4799at7088 | Complete | Missing |
| 479at7088 | Complete | Missing |
| 47at7088 | Complete | Missing |
| 4802at7088 | Duplicated | Missing |
| 4806at7088 | Complete | Missing |
| 4810at7088 | Complete | Missing |
| 4811at7088 | Fragmented | Missing |
| 4812at7088 | Complete | Missing |
| 4816at7088 | Complete | Missing |
| 481at7088 | Complete | Missing |
| 4820at7088 | Duplicated | Missing |
| 4821at7088 | Complete | Missing |
| 4823at7088 | Complete | Missing |
| 4824at7088 | Complete | Missing |
| 4834at7088 | Complete | Missing |
| 4837at7088 | Complete | Missing |
| 4838at7088 | Complete | Missing |
| 4839at7088 | Duplicated | Missing |
| 4842at7088 | Complete | Missing |
| 4845at7088 | Complete | Missing |
| 4847at7088 | Complete | Missing |
| 4848at7088 | Complete | Missing |
| 4849at7088 | Complete | Missing |
| 4851at7088 | Complete | Missing |
| 4854at7088 | Complete | Complete |
| 4855at7088 | Complete | Missing |
| 4858at7088 | Complete | Complete |
| 4860at7088 | Complete | Missing |
| 4864at7088 | Complete | Missing |
| 4866at7088 | Complete | Fragmented |
| 4869at7088 | Missing | Fragmented |
| 486at7088 | Duplicated | Missing |
| 4870at7088 | Complete | Missing |
| 4872at7088 | Complete | Missing |
| 4877at7088 | Complete | Missing |
| 4878at7088 | Complete | Missing |
| 4880at7088 | Duplicated | Missing |
| 4884at7088 | Complete | Missing |
| 4887at7088 | Complete | Missing |
| 4889at7088 | Complete | Missing |
| 488at7088 | Complete | Missing |
| 4891at7088 | Complete | Missing |
| 4893at7088 | Complete | Missing |
| 4894at7088 | Complete | Missing |
| 4896at7088 | Complete | Missing |
| 4899at7088 | Complete | Missing |
| 489at7088 | Complete | Missing |
| 48at7088 | Complete | Missing |
| 4900at7088 | Complete | Missing |
| 4908at7088 | Complete | Missing |
| 490at7088 | Complete | Missing |
| 4910at7088 | Duplicated | Missing |
| 4915at7088 | Complete | Missing |
| 4916at7088 | Complete | Missing |
| 4918at7088 | Complete | Missing |
| 4922at7088 | Complete | Missing |
| 4923at7088 | Complete | Missing |
| 4929at7088 | Duplicated | Missing |
| 492at7088 | Complete | Missing |
| 4930at7088 | Complete | Missing |
| 4932at7088 | Complete | Missing |
| 4934at7088 | Missing | Missing |
| 4935at7088 | Complete | Missing |
| 4936at7088 | Complete | Fragmented |
| 4942at7088 | Duplicated | Missing |
| 4948at7088 | Complete | Missing |
| 4949at7088 | Complete | Missing |
| 494at7088 | Complete | Missing |
| 4950at7088 | Complete | Missing |
| 4952at7088 | Complete | Missing |
| 4953at7088 | Duplicated | Missing |
| 4954at7088 | Complete | Missing |
| 4955at7088 | Complete | Missing |
| 4956at7088 | Complete | Missing |
| 4958at7088 | Complete | Missing |
| 4959at7088 | Complete | Missing |
| 495at7088 | Complete | Missing |
| 4964at7088 | Complete | Missing |
| 4967at7088 | Complete | Missing |
| 4969at7088 | Complete | Missing |
| 496at7088 | Complete | Duplicated |
| 4974at7088 | Complete | Missing |
| 4975at7088 | Complete | Missing |
| 4989at7088 | Complete | Missing |
| 498at7088 | Complete | Missing |
| 4990at7088 | Complete | Missing |
| 4993at7088 | Complete | Missing |
| 4996at7088 | Complete | Missing |
| 4998at7088 | Complete | Missing |
| 499at7088 | Complete | Missing |
| 49at7088 | Fragmented | Missing |
| 4at7088 | Complete | Missing |
| 5002at7088 | Complete | Missing |
| 5004at7088 | Complete | Missing |
| 5006at7088 | Complete | Missing |
| 5009at7088 | Complete | Missing |
| 5012at7088 | Complete | Fragmented |
| 5016at7088 | Complete | Fragmented |
| 5019at7088 | Complete | Missing |
| 5021at7088 | Complete | Missing |
| 5026at7088 | Complete | Missing |
| 5030at7088 | Complete | Fragmented |
| 5031at7088 | Complete | Missing |
| 5035at7088 | Complete | Missing |
| 5036at7088 | Complete | Missing |
| 5038at7088 | Complete | Fragmented |
| 5040at7088 | Complete | Missing |
| 5044at7088 | Complete | Missing |
| 5046at7088 | Complete | Missing |
| 5048at7088 | Duplicated | Missing |
| 504at7088 | Complete | Missing |
| 5050at7088 | Missing | Missing |
| 5051at7088 | Missing | Missing |
| 5053at7088 | Complete | Missing |
| 5055at7088 | Complete | Missing |
| 5056at7088 | Complete | Missing |
| 5057at7088 | Complete | Missing |
| 505at7088 | Complete | Missing |
| 5064at7088 | Complete | Missing |
| 5066at7088 | Complete | Fragmented |
| 506at7088 | Complete | Missing |
| 5070at7088 | Complete | Missing |
| 5074at7088 | Complete | Missing |
| 5075at7088 | Complete | Missing |
| 5078at7088 | Complete | Missing |
| 5082at7088 | Duplicated | Missing |
| 5083at7088 | Duplicated | Missing |
| 5091at7088 | Duplicated | Complete |
| 5093at7088 | Fragmented | Missing |
| 5094at7088 | Duplicated | Missing |
| 5095at7088 | Fragmented | Missing |
| 5098at7088 | Complete | Missing |
| 5099at7088 | Complete | Missing |
| 509at7088 | Complete | Missing |
| 50at7088 | Complete | Missing |
| 5101at7088 | Complete | Missing |
| 5107at7088 | Complete | Missing |
| 5108at7088 | Complete | Missing |
| 5110at7088 | Complete | Missing |
| 5114at7088 | Complete | Missing |
| 511at7088 | Complete | Missing |
| 5120at7088 | Complete | Missing |
| 5128at7088 | Complete | Missing |
| 5129at7088 | Complete | Missing |
| 5130at7088 | Missing | Missing |
| 5135at7088 | Complete | Missing |
| 5139at7088 | Complete | Missing |
| 513at7088 | Complete | Missing |
| 5144at7088 | Complete | Missing |
| 5145at7088 | Complete | Missing |
| 5146at7088 | Complete | Missing |
| 5147at7088 | Complete | Missing |
| 5149at7088 | Complete | Missing |
| 5151at7088 | Complete | Missing |
| 5157at7088 | Duplicated | Missing |
| 5159at7088 | Complete | Missing |
| 515at7088 | Complete | Missing |
| 5162at7088 | Duplicated | Missing |
| 5164at7088 | Complete | Missing |
| 5166at7088 | Complete | Missing |
| 5168at7088 | Complete | Missing |
| 516at7088 | Complete | Missing |
| 5172at7088 | Complete | Missing |
| 5173at7088 | Complete | Missing |
| 517at7088 | Fragmented | Missing |
| 5181at7088 | Complete | Missing |
| 5182at7088 | Complete | Missing |
| 5183at7088 | Complete | Fragmented |
| 5184at7088 | Complete | Missing |
| 5187at7088 | Complete | Missing |
| 518at7088 | Complete | Missing |
| 5190at7088 | Fragmented | Missing |
| 5191at7088 | Complete | Missing |
| 5193at7088 | Complete | Missing |
| 5194at7088 | Missing | Missing |
| 5196at7088 | Complete | Missing |
| 5197at7088 | Complete | Missing |
| 5199at7088 | Complete | Missing |
| 519at7088 | Complete | Missing |
| 51at7088 | Missing | Missing |
| 5201at7088 | Complete | Missing |
| 5203at7088 | Complete | Missing |
| 5205at7088 | Missing | Missing |
| 5206at7088 | Complete | Missing |
| 5213at7088 | Complete | Fragmented |
| 5216at7088 | Complete | Missing |
| 5219at7088 | Complete | Missing |
| 5223at7088 | Complete | Missing |
| 5227at7088 | Complete | Missing |
| 522at7088 | Complete | Fragmented |
| 5239at7088 | Complete | Missing |
| 5242at7088 | Complete | Missing |
| 5244at7088 | Complete | Complete |
| 5249at7088 | Complete | Missing |
| 5250at7088 | Complete | Missing |
| 5251at7088 | Complete | Missing |
| 5252at7088 | Complete | Missing |
| 5256at7088 | Complete | Missing |
| 5258at7088 | Complete | Missing |
| 5260at7088 | Complete | Missing |
| 5265at7088 | Complete | Missing |
| 5266at7088 | Complete | Missing |
| 526at7088 | Complete | Missing |
| 5270at7088 | Complete | Fragmented |
| 5277at7088 | Complete | Missing |
| 527at7088 | Complete | Missing |
| 5281at7088 | Missing | Fragmented |
| 5284at7088 | Complete | Missing |
| 5290at7088 | Duplicated | Missing |
| 5291at7088 | Duplicated | Missing |
| 5292at7088 | Complete | Missing |
| 5293at7088 | Missing | Missing |
| 5295at7088 | Complete | Missing |
| 5299at7088 | Complete | Missing |
| 52at7088 | Complete | Missing |
| 5301at7088 | Complete | Missing |
| 5302at7088 | Complete | Missing |
| 5303at7088 | Complete | Missing |
| 530at7088 | Complete | Missing |
| 5311at7088 | Complete | Missing |
| 5312at7088 | Complete | Missing |
| 5313at7088 | Complete | Missing |
| 5314at7088 | Duplicated | Missing |
| 5317at7088 | Duplicated | Missing |
| 5319at7088 | Complete | Missing |
| 531at7088 | Complete | Missing |
| 5321at7088 | Complete | Missing |
| 5322at7088 | Complete | Missing |
| 5323at7088 | Complete | Fragmented |
| 5326at7088 | Complete | Missing |
| 5327at7088 | Complete | Missing |
| 5328at7088 | Complete | Missing |
| 532at7088 | Complete | Missing |
| 5333at7088 | Complete | Missing |
| 5337at7088 | Complete | Missing |
| 5341at7088 | Complete | Missing |
| 5342at7088 | Complete | Missing |
| 5351at7088 | Complete | Missing |
| 5355at7088 | Complete | Missing |
| 5361at7088 | Fragmented | Missing |
| 5362at7088 | Complete | Missing |
| 5364at7088 | Complete | Missing |
| 5369at7088 | Complete | Missing |
| 5370at7088 | Complete | Missing |
| 5371at7088 | Complete | Missing |
| 5373at7088 | Complete | Missing |
| 5377at7088 | Complete | Missing |
| 5380at7088 | Duplicated | Missing |
| 5381at7088 | Complete | Missing |
| 5383at7088 | Complete | Missing |
| 5385at7088 | Complete | Missing |
| 538at7088 | Complete | Missing |
| 5391at7088 | Complete | Missing |
| 5392at7088 | Complete | Missing |
| 5394at7088 | Complete | Missing |
| 5397at7088 | Complete | Missing |
| 5398at7088 | Complete | Fragmented |
| 5404at7088 | Complete | Missing |
| 5406at7088 | Complete | Missing |
| 5410at7088 | Complete | Missing |
| 5413at7088 | Duplicated | Missing |
| 5415at7088 | Complete | Missing |
| 5423at7088 | Duplicated | Missing |
| 5424at7088 | Complete | Missing |
| 5425at7088 | Complete | Missing |
| 5427at7088 | Complete | Complete |
| 543at7088 | Fragmented | Missing |
| 5440at7088 | Complete | Missing |
| 5443at7088 | Complete | Missing |
| 5446at7088 | Complete | Fragmented |
| 544at7088 | Complete | Missing |
| 5450at7088 | Complete | Missing |
| 5453at7088 | Complete | Missing |
| 5455at7088 | Complete | Missing |
| 5457at7088 | Complete | Missing |
| 5463at7088 | Complete | Missing |
| 5464at7088 | Complete | Missing |
| 5474at7088 | Complete | Missing |
| 547at7088 | Complete | Missing |
| 5480at7088 | Complete | Missing |
| 5483at7088 | Missing | Missing |
| 5486at7088 | Complete | Complete |
| 5487at7088 | Complete | Missing |
| 5488at7088 | Fragmented | Missing |
| 5496at7088 | Complete | Missing |
| 54at7088 | Complete | Missing |
| 5500at7088 | Complete | Missing |
| 5506at7088 | Complete | Missing |
| 5507at7088 | Complete | Missing |
| 5508at7088 | Complete | Missing |
| 550at7088 | Complete | Missing |
| 5520at7088 | Duplicated | Missing |
| 5523at7088 | Missing | Missing |
| 552at7088 | Complete | Missing |
| 5530at7088 | Complete | Missing |
| 5531at7088 | Complete | Missing |
| 5533at7088 | Complete | Missing |
| 5535at7088 | Complete | Missing |
| 5536at7088 | Complete | Missing |
| 5538at7088 | Complete | Missing |
| 5539at7088 | Complete | Missing |
| 553at7088 | Complete | Missing |
| 5541at7088 | Complete | Missing |
| 5547at7088 | Complete | Missing |
| 5550at7088 | Complete | Missing |
| 5555at7088 | Complete | Missing |
| 5556at7088 | Complete | Complete |
| 5557at7088 | Missing | Missing |
| 5558at7088 | Complete | Missing |
| 5559at7088 | Complete | Missing |
| 5560at7088 | Complete | Missing |
| 5563at7088 | Complete | Missing |
| 5565at7088 | Duplicated | Missing |
| 5567at7088 | Complete | Missing |
| 556at7088 | Complete | Missing |
| 5570at7088 | Complete | Missing |
| 5571at7088 | Complete | Missing |
| 5576at7088 | Complete | Missing |
| 5580at7088 | Complete | Complete |
| 5584at7088 | Complete | Missing |
| 5585at7088 | Fragmented | Missing |
| 5586at7088 | Complete | Missing |
| 5587at7088 | Complete | Missing |
| 5589at7088 | Complete | Missing |
| 558at7088 | Missing | Missing |
| 5595at7088 | Complete | Missing |
| 5598at7088 | Duplicated | Missing |
| 5599at7088 | Complete | Missing |
| 559at7088 | Complete | Missing |
| 5603at7088 | Complete | Missing |
| 5606at7088 | Complete | Missing |
| 5611at7088 | Complete | Missing |
| 5614at7088 | Complete | Missing |
| 5616at7088 | Complete | Missing |
| 5622at7088 | Complete | Missing |
| 5623at7088 | Complete | Missing |
| 5625at7088 | Complete | Missing |
| 5626at7088 | Complete | Complete |
| 5627at7088 | Complete | Missing |
| 5628at7088 | Complete | Missing |
| 562at7088 | Complete | Missing |
| 5634at7088 | Complete | Missing |
| 5636at7088 | Complete | Missing |
| 5638at7088 | Missing | Missing |
| 5639at7088 | Complete | Missing |
| 563at7088 | Complete | Missing |
| 5643at7088 | Complete | Missing |
| 5648at7088 | Complete | Missing |
| 5650at7088 | Complete | Fragmented |
| 5651at7088 | Complete | Missing |
| 5653at7088 | Missing | Missing |
| 5665at7088 | Duplicated | Missing |
| 5668at7088 | Complete | Missing |
| 566at7088 | Complete | Missing |
| 5671at7088 | Complete | Missing |
| 5672at7088 | Complete | Missing |
| 5674at7088 | Complete | Missing |
| 5676at7088 | Complete | Missing |
| 5679at7088 | Complete | Fragmented |
| 5689at7088 | Complete | Missing |
| 5690at7088 | Duplicated | Missing |
| 5693at7088 | Complete | Missing |
| 5694at7088 | Fragmented | Missing |
| 5699at7088 | Complete | Missing |
| 569at7088 | Complete | Missing |
| 56at7088 | Complete | Missing |
| 5700at7088 | Complete | Missing |
| 5702at7088 | Complete | Missing |
| 5703at7088 | Complete | Missing |
| 5705at7088 | Complete | Missing |
| 5708at7088 | Fragmented | Missing |
| 5710at7088 | Duplicated | Missing |
| 5711at7088 | Missing | Missing |
| 5712at7088 | Complete | Complete |
| 5713at7088 | Fragmented | Missing |
| 5715at7088 | Complete | Missing |
| 5716at7088 | Complete | Missing |
| 5717at7088 | Complete | Missing |
| 571at7088 | Complete | Missing |
| 5721at7088 | Complete | Missing |
| 5722at7088 | Complete | Missing |
| 5723at7088 | Complete | Missing |
| 5724at7088 | Complete | Missing |
| 5728at7088 | Complete | Missing |
| 572at7088 | Complete | Missing |
| 5732at7088 | Complete | Missing |
| 5733at7088 | Complete | Missing |
| 5734at7088 | Complete | Missing |
| 5735at7088 | Missing | Fragmented |
| 5738at7088 | Complete | Missing |
| 5740at7088 | Complete | Missing |
| 5742at7088 | Complete | Missing |
| 5743at7088 | Complete | Missing |
| 5748at7088 | Complete | Missing |
| 5759at7088 | Complete | Missing |
| 5761at7088 | Missing | Missing |
| 5762at7088 | Complete | Missing |
| 5765at7088 | Complete | Missing |
| 5767at7088 | Complete | Missing |
| 5772at7088 | Complete | Missing |
| 5773at7088 | Duplicated | Missing |
| 5775at7088 | Complete | Missing |
| 5776at7088 | Complete | Missing |
| 5781at7088 | Complete | Missing |
| 5782at7088 | Complete | Missing |
| 5786at7088 | Complete | Missing |
| 5787at7088 | Complete | Missing |
| 578at7088 | Complete | Missing |
| 5792at7088 | Complete | Complete |
| 5793at7088 | Complete | Missing |
| 5794at7088 | Complete | Missing |
| 5795at7088 | Complete | Missing |
| 5797at7088 | Complete | Missing |
| 5798at7088 | Complete | Missing |
| 5799at7088 | Complete | Missing |
| 57at7088 | Complete | Missing |
| 5802at7088 | Complete | Missing |
| 5806at7088 | Missing | Missing |
| 5810at7088 | Complete | Missing |
| 5812at7088 | Complete | Missing |
| 5817at7088 | Duplicated | Missing |
| 5819at7088 | Complete | Missing |
| 5820at7088 | Complete | Missing |
| 5821at7088 | Complete | Missing |
| 5822at7088 | Complete | Missing |
| 5825at7088 | Missing | Missing |
| 5826at7088 | Complete | Missing |
| 5827at7088 | Complete | Missing |
| 5828at7088 | Complete | Missing |
| 5832at7088 | Complete | Missing |
| 5833at7088 | Complete | Missing |
| 5835at7088 | Complete | Missing |
| 5837at7088 | Fragmented | Missing |
| 583at7088 | Complete | Missing |
| 5840at7088 | Complete | Missing |
| 5844at7088 | Complete | Missing |
| 584at7088 | Complete | Missing |
| 5851at7088 | Missing | Missing |
| 5852at7088 | Complete | Missing |
| 5858at7088 | Duplicated | Missing |
| 5863at7088 | Complete | Missing |
| 5864at7088 | Complete | Missing |
| 5866at7088 | Complete | Missing |
| 5873at7088 | Complete | Missing |
| 5878at7088 | Complete | Fragmented |
| 5881at7088 | Complete | Missing |
| 5884at7088 | Complete | Missing |
| 5885at7088 | Complete | Missing |
| 5887at7088 | Complete | Missing |
| 5889at7088 | Complete | Missing |
| 5890at7088 | Complete | Missing |
| 5891at7088 | Complete | Missing |
| 5898at7088 | Missing | Missing |
| 589at7088 | Complete | Missing |
| 5900at7088 | Duplicated | Missing |
| 590at7088 | Fragmented | Missing |
| 5912at7088 | Duplicated | Missing |
| 5916at7088 | Complete | Missing |
| 5917at7088 | Complete | Missing |
| 5919at7088 | Complete | Missing |
| 5920at7088 | Complete | Missing |
| 5922at7088 | Complete | Fragmented |
| 5925at7088 | Complete | Missing |
| 5933at7088 | Complete | Missing |
| 5936at7088 | Fragmented | Missing |
| 5937at7088 | Duplicated | Missing |
| 5938at7088 | Complete | Missing |
| 5945at7088 | Complete | Missing |
| 5949at7088 | Missing | Fragmented |
| 5950at7088 | Complete | Missing |
| 5952at7088 | Complete | Missing |
| 5954at7088 | Complete | Missing |
| 5957at7088 | Fragmented | Missing |
| 5963at7088 | Complete | Missing |
| 5964at7088 | Complete | Fragmented |
| 5967at7088 | Complete | Missing |
| 5969at7088 | Complete | Missing |
| 5974at7088 | Complete | Missing |
| 5975at7088 | Complete | Missing |
| 5978at7088 | Complete | Missing |
| 5979at7088 | Complete | Missing |
| 5980at7088 | Complete | Missing |
| 5982at7088 | Complete | Missing |
| 5987at7088 | Complete | Missing |
| 598at7088 | Complete | Missing |
| 5991at7088 | Complete | Missing |
| 599at7088 | Complete | Fragmented |
| 59at7088 | Fragmented | Missing |
| 6000at7088 | Complete | Missing |
| 6001at7088 | Duplicated | Missing |
| 6006at7088 | Complete | Missing |
| 6007at7088 | Complete | Missing |
| 600at7088 | Complete | Missing |
| 6012at7088 | Complete | Missing |
| 6013at7088 | Complete | Missing |
| 601at7088 | Complete | Missing |
| 6020at7088 | Complete | Missing |
| 6021at7088 | Duplicated | Missing |
| 6022at7088 | Complete | Missing |
| 6023at7088 | Complete | Missing |
| 6025at7088 | Complete | Missing |
| 6031at7088 | Duplicated | Fragmented |
| 6032at7088 | Complete | Missing |
| 6033at7088 | Complete | Missing |
| 6038at7088 | Complete | Missing |
| 603at7088 | Duplicated | Missing |
| 6043at7088 | Complete | Missing |
| 6044at7088 | Complete | Missing |
| 6047at7088 | Complete | Missing |
| 6050at7088 | Complete | Missing |
| 6051at7088 | Complete | Missing |
| 6055at7088 | Complete | Missing |
| 6061at7088 | Complete | Missing |
| 6062at7088 | Complete | Missing |
| 6063at7088 | Duplicated | Missing |
| 6066at7088 | Complete | Missing |
| 6067at7088 | Complete | Complete |
| 6074at7088 | Complete | Missing |
| 6076at7088 | Duplicated | Missing |
| 6077at7088 | Complete | Missing |
| 6079at7088 | Complete | Missing |
| 6080at7088 | Complete | Missing |
| 6081at7088 | Complete | Missing |
| 6083at7088 | Missing | Missing |
| 6084at7088 | Complete | Missing |
| 6085at7088 | Complete | Missing |
| 6089at7088 | Complete | Missing |
| 608at7088 | Complete | Missing |
| 6091at7088 | Complete | Missing |
| 6093at7088 | Complete | Missing |
| 6097at7088 | Complete | Missing |
| 6099at7088 | Complete | Missing |
| 609at7088 | Complete | Missing |
| 60at7088 | Missing | Missing |
| 6101at7088 | Complete | Missing |
| 6104at7088 | Complete | Missing |
| 6105at7088 | Complete | Missing |
| 6108at7088 | Complete | Missing |
| 6109at7088 | Complete | Missing |
| 6110at7088 | Complete | Missing |
| 6112at7088 | Complete | Missing |
| 6115at7088 | Complete | Missing |
| 6116at7088 | Complete | Missing |
| 6117at7088 | Complete | Missing |
| 6119at7088 | Complete | Missing |
| 611at7088 | Duplicated | Missing |
| 6122at7088 | Complete | Missing |
| 6123at7088 | Complete | Missing |
| 6126at7088 | Complete | Missing |
| 6129at7088 | Complete | Missing |
| 6130at7088 | Complete | Missing |
| 6131at7088 | Complete | Missing |
| 6135at7088 | Complete | Missing |
| 6139at7088 | Complete | Missing |
| 613at7088 | Complete | Missing |
| 6140at7088 | Duplicated | Missing |
| 6143at7088 | Complete | Missing |
| 6145at7088 | Fragmented | Missing |
| 614at7088 | Complete | Missing |
| 6156at7088 | Complete | Missing |
| 6157at7088 | Complete | Missing |
| 6159at7088 | Complete | Missing |
| 6162at7088 | Complete | Missing |
| 6166at7088 | Complete | Missing |
| 616at7088 | Complete | Complete |
| 6171at7088 | Complete | Missing |
| 6172at7088 | Complete | Missing |
| 6173at7088 | Complete | Fragmented |
| 6174at7088 | Complete | Missing |
| 6175at7088 | Duplicated | Complete |
| 6178at7088 | Complete | Complete |
| 6179at7088 | Duplicated | Missing |
| 6180at7088 | Duplicated | Missing |
| 6181at7088 | Complete | Missing |
| 6186at7088 | Duplicated | Missing |
| 6191at7088 | Complete | Missing |
| 6196at7088 | Missing | Missing |
| 6198at7088 | Duplicated | Missing |
| 6199at7088 | Complete | Missing |
| 6201at7088 | Missing | Missing |
| 6205at7088 | Complete | Missing |
| 6207at7088 | Duplicated | Missing |
| 6208at7088 | Complete | Missing |
| 6209at7088 | Complete | Missing |
| 620at7088 | Complete | Missing |
| 6210at7088 | Complete | Missing |
| 6213at7088 | Complete | Missing |
| 6214at7088 | Complete | Missing |
| 6215at7088 | Complete | Missing |
| 6216at7088 | Complete | Missing |
| 6218at7088 | Complete | Missing |
| 6219at7088 | Complete | Missing |
| 621at7088 | Complete | Missing |
| 6221at7088 | Complete | Missing |
| 6225at7088 | Complete | Missing |
| 6226at7088 | Complete | Missing |
| 622at7088 | Duplicated | Missing |
| 6230at7088 | Complete | Fragmented |
| 6239at7088 | Complete | Missing |
| 6240at7088 | Complete | Missing |
| 6241at7088 | Duplicated | Missing |
| 6242at7088 | Complete | Missing |
| 6243at7088 | Complete | Missing |
| 6247at7088 | Complete | Missing |
| 6249at7088 | Complete | Missing |
| 6253at7088 | Complete | Missing |
| 6255at7088 | Fragmented | Missing |
| 6261at7088 | Complete | Missing |
| 6263at7088 | Complete | Missing |
| 6264at7088 | Complete | Missing |
| 6265at7088 | Complete | Missing |
| 6267at7088 | Complete | Missing |
| 6269at7088 | Fragmented | Missing |
| 6273at7088 | Complete | Missing |
| 6279at7088 | Complete | Missing |
| 627at7088 | Complete | Missing |
| 6284at7088 | Complete | Missing |
| 6285at7088 | Missing | Missing |
| 6287at7088 | Complete | Missing |
| 6289at7088 | Complete | Missing |
| 628at7088 | Complete | Fragmented |
| 6290at7088 | Complete | Missing |
| 6294at7088 | Complete | Missing |
| 6295at7088 | Missing | Missing |
| 6299at7088 | Complete | Missing |
| 6300at7088 | Complete | Missing |
| 6302at7088 | Duplicated | Missing |
| 6308at7088 | Fragmented | Missing |
| 6309at7088 | Complete | Missing |
| 6311at7088 | Missing | Missing |
| 6320at7088 | Complete | Missing |
| 6321at7088 | Complete | Missing |
| 6325at7088 | Complete | Missing |
| 6329at7088 | Complete | Missing |
| 632at7088 | Complete | Missing |
| 6330at7088 | Complete | Missing |
| 6332at7088 | Complete | Fragmented |
| 6335at7088 | Complete | Missing |
| 6336at7088 | Complete | Missing |
| 6339at7088 | Complete | Missing |
| 633at7088 | Complete | Missing |
| 6342at7088 | Complete | Complete |
| 6343at7088 | Complete | Missing |
| 6346at7088 | Complete | Missing |
| 6347at7088 | Complete | Missing |
| 6348at7088 | Complete | Missing |
| 6349at7088 | Complete | Missing |
| 6351at7088 | Complete | Missing |
| 6352at7088 | Complete | Missing |
| 6353at7088 | Complete | Missing |
| 6358at7088 | Complete | Missing |
| 6365at7088 | Duplicated | Missing |
| 6366at7088 | Complete | Missing |
| 6373at7088 | Complete | Missing |
| 6374at7088 | Complete | Missing |
| 637at7088 | Complete | Missing |
| 6381at7088 | Complete | Missing |
| 6385at7088 | Duplicated | Missing |
| 6386at7088 | Complete | Missing |
| 638at7088 | Complete | Fragmented |
| 6394at7088 | Complete | Missing |
| 6395at7088 | Complete | Missing |
| 639at7088 | Complete | Missing |
| 63at7088 | Complete | Missing |
| 6401at7088 | Complete | Missing |
| 6403at7088 | Complete | Missing |
| 6404at7088 | Duplicated | Complete |
| 6407at7088 | Complete | Missing |
| 6410at7088 | Fragmented | Missing |
| 6413at7088 | Complete | Missing |
| 6414at7088 | Complete | Missing |
| 6420at7088 | Complete | Missing |
| 6421at7088 | Complete | Missing |
| 6427at7088 | Complete | Missing |
| 642at7088 | Complete | Missing |
| 6432at7088 | Complete | Missing |
| 6433at7088 | Complete | Missing |
| 6436at7088 | Complete | Missing |
| 6437at7088 | Duplicated | Missing |
| 6438at7088 | Complete | Missing |
| 6439at7088 | Complete | Missing |
| 643at7088 | Complete | Missing |
| 6443at7088 | Complete | Missing |
| 6446at7088 | Complete | Missing |
| 6449at7088 | Complete | Missing |
| 6450at7088 | Missing | Missing |
| 6453at7088 | Fragmented | Missing |
| 6454at7088 | Complete | Missing |
| 6462at7088 | Complete | Missing |
| 6464at7088 | Complete | Missing |
| 6467at7088 | Complete | Missing |
| 6471at7088 | Complete | Missing |
| 6477at7088 | Missing | Missing |
| 647at7088 | Complete | Missing |
| 6481at7088 | Complete | Missing |
| 6486at7088 | Complete | Missing |
| 6487at7088 | Complete | Missing |
| 6499at7088 | Complete | Missing |
| 649at7088 | Complete | Missing |
| 64at7088 | Complete | Missing |
| 6501at7088 | Complete | Missing |
| 6502at7088 | Complete | Missing |
| 6503at7088 | Duplicated | Missing |
| 6508at7088 | Complete | Fragmented |
| 6512at7088 | Duplicated | Missing |
| 6513at7088 | Complete | Missing |
| 6515at7088 | Complete | Missing |
| 6518at7088 | Complete | Missing |
| 6522at7088 | Complete | Missing |
| 6523at7088 | Complete | Missing |
| 6525at7088 | Missing | Missing |
| 6528at7088 | Duplicated | Missing |
| 652at7088 | Duplicated | Missing |
| 6538at7088 | Complete | Missing |
| 6540at7088 | Complete | Missing |
| 6542at7088 | Missing | Missing |
| 6548at7088 | Complete | Missing |
| 6552at7088 | Complete | Missing |
| 6555at7088 | Duplicated | Fragmented |
| 6556at7088 | Complete | Missing |
| 6558at7088 | Complete | Missing |
| 655at7088 | Complete | Missing |
| 6562at7088 | Complete | Missing |
| 6570at7088 | Complete | Missing |
| 6571at7088 | Complete | Missing |
| 6572at7088 | Complete | Missing |
| 6573at7088 | Fragmented | Missing |
| 6579at7088 | Complete | Missing |
| 657at7088 | Complete | Missing |
| 6582at7088 | Duplicated | Missing |
| 6583at7088 | Complete | Missing |
| 6585at7088 | Duplicated | Missing |
| 6589at7088 | Complete | Missing |
| 658at7088 | Complete | Missing |
| 6594at7088 | Complete | Missing |
| 6597at7088 | Complete | Missing |
| 6598at7088 | Complete | Missing |
| 659at7088 | Complete | Missing |
| 65at7088 | Complete | Missing |
| 6601at7088 | Complete | Complete |
| 6602at7088 | Complete | Missing |
| 6606at7088 | Complete | Missing |
| 6607at7088 | Duplicated | Missing |
| 660at7088 | Complete | Missing |
| 6614at7088 | Complete | Missing |
| 6616at7088 | Complete | Missing |
| 6620at7088 | Complete | Missing |
| 6622at7088 | Duplicated | Missing |
| 6627at7088 | Complete | Missing |
| 6628at7088 | Complete | Missing |
| 662at7088 | Complete | Missing |
| 6636at7088 | Complete | Missing |
| 6637at7088 | Complete | Missing |
| 6642at7088 | Complete | Missing |
| 6644at7088 | Complete | Missing |
| 6645at7088 | Complete | Missing |
| 6646at7088 | Complete | Missing |
| 664at7088 | Complete | Missing |
| 6652at7088 | Duplicated | Missing |
| 6655at7088 | Complete | Missing |
| 6657at7088 | Duplicated | Missing |
| 6663at7088 | Missing | Missing |
| 6668at7088 | Complete | Missing |
| 666at7088 | Complete | Missing |
| 6670at7088 | Complete | Missing |
| 6671at7088 | Complete | Missing |
| 6673at7088 | Complete | Duplicated |
| 6674at7088 | Duplicated | Missing |
| 6678at7088 | Fragmented | Missing |
| 667at7088 | Complete | Missing |
| 6680at7088 | Complete | Missing |
| 6681at7088 | Complete | Missing |
| 6683at7088 | Complete | Missing |
| 6684at7088 | Complete | Missing |
| 6687at7088 | Complete | Missing |
| 6688at7088 | Complete | Missing |
| 6690at7088 | Complete | Missing |
| 6691at7088 | Complete | Missing |
| 66at7088 | Complete | Missing |
| 6701at7088 | Complete | Missing |
| 6703at7088 | Complete | Missing |
| 6707at7088 | Complete | Missing |
| 670at7088 | Complete | Missing |
| 6720at7088 | Missing | Missing |
| 6722at7088 | Missing | Missing |
| 6723at7088 | Missing | Missing |
| 6725at7088 | Complete | Missing |
| 6728at7088 | Complete | Missing |
| 672at7088 | Complete | Fragmented |
| 6737at7088 | Complete | Missing |
| 6742at7088 | Duplicated | Missing |
| 6744at7088 | Duplicated | Missing |
| 6745at7088 | Complete | Missing |
| 6751at7088 | Complete | Missing |
| 6753at7088 | Complete | Missing |
| 6754at7088 | Complete | Missing |
| 6757at7088 | Missing | Missing |
| 675at7088 | Complete | Missing |
| 6769at7088 | Complete | Missing |
| 6770at7088 | Complete | Missing |
| 6771at7088 | Complete | Missing |
| 6775at7088 | Complete | Missing |
| 6779at7088 | Complete | Missing |
| 6780at7088 | Complete | Missing |
| 6782at7088 | Complete | Missing |
| 6783at7088 | Complete | Missing |
| 6786at7088 | Duplicated | Missing |
| 6787at7088 | Complete | Missing |
| 6788at7088 | Complete | Missing |
| 6789at7088 | Complete | Missing |
| 678at7088 | Complete | Missing |
| 6791at7088 | Complete | Missing |
| 6795at7088 | Complete | Missing |
| 6796at7088 | Fragmented | Missing |
| 6797at7088 | Complete | Missing |
| 6798at7088 | Complete | Missing |
| 6809at7088 | Complete | Complete |
| 680at7088 | Complete | Missing |
| 6810at7088 | Complete | Missing |
| 6811at7088 | Complete | Missing |
| 6817at7088 | Complete | Missing |
| 6818at7088 | Fragmented | Missing |
| 6819at7088 | Fragmented | Missing |
| 681at7088 | Complete | Missing |
| 6821at7088 | Complete | Missing |
| 6823at7088 | Complete | Missing |
| 6825at7088 | Complete | Missing |
| 6826at7088 | Complete | Missing |
| 6830at7088 | Complete | Missing |
| 6835at7088 | Duplicated | Missing |
| 6837at7088 | Complete | Missing |
| 6841at7088 | Complete | Missing |
| 6845at7088 | Complete | Missing |
| 6846at7088 | Duplicated | Missing |
| 6847at7088 | Complete | Missing |
| 6853at7088 | Complete | Missing |
| 6855at7088 | Complete | Missing |
| 6857at7088 | Complete | Missing |
| 6860at7088 | Complete | Missing |
| 6861at7088 | Complete | Missing |
| 6862at7088 | Complete | Missing |
| 6871at7088 | Complete | Missing |
| 6873at7088 | Complete | Missing |
| 6874at7088 | Complete | Missing |
| 6884at7088 | Complete | Missing |
| 6885at7088 | Complete | Missing |
| 6893at7088 | Complete | Missing |
| 6896at7088 | Complete | Missing |
| 6899at7088 | Duplicated | Missing |
| 68at7088 | Complete | Missing |
| 6900at7088 | Complete | Missing |
| 6903at7088 | Complete | Missing |
| 6906at7088 | Complete | Missing |
| 6908at7088 | Complete | Missing |
| 6909at7088 | Complete | Missing |
| 690at7088 | Duplicated | Missing |
| 6912at7088 | Complete | Missing |
| 6913at7088 | Complete | Missing |
| 6914at7088 | Complete | Missing |
| 6918at7088 | Complete | Missing |
| 6919at7088 | Complete | Missing |
| 6926at7088 | Duplicated | Missing |
| 692at7088 | Complete | Missing |
| 6935at7088 | Complete | Missing |
| 6936at7088 | Complete | Fragmented |
| 6937at7088 | Duplicated | Missing |
| 6938at7088 | Complete | Missing |
| 6940at7088 | Complete | Missing |
| 6944at7088 | Complete | Missing |
| 6950at7088 | Complete | Missing |
| 6951at7088 | Complete | Missing |
| 6955at7088 | Complete | Missing |
| 6959at7088 | Complete | Fragmented |
| 695at7088 | Complete | Missing |
| 6967at7088 | Complete | Missing |
| 696at7088 | Complete | Missing |
| 6971at7088 | Complete | Missing |
| 6979at7088 | Complete | Missing |
| 6980at7088 | Complete | Missing |
| 6982at7088 | Complete | Missing |
| 6983at7088 | Duplicated | Missing |
| 6985at7088 | Complete | Missing |
| 6987at7088 | Complete | Missing |
| 6988at7088 | Complete | Missing |
| 6989at7088 | Complete | Missing |
| 6991at7088 | Complete | Missing |
| 6996at7088 | Duplicated | Missing |
| 69at7088 | Complete | Missing |
| 6at7088 | Complete | Missing |
| 7001at7088 | Complete | Missing |
| 7002at7088 | Complete | Missing |
| 7004at7088 | Complete | Missing |
| 7005at7088 | Missing | Missing |
| 7007at7088 | Complete | Missing |
| 7009at7088 | Complete | Missing |
| 7010at7088 | Complete | Missing |
| 7017at7088 | Complete | Missing |
| 701at7088 | Complete | Missing |
| 7024at7088 | Complete | Missing |
| 7025at7088 | Complete | Missing |
| 7040at7088 | Fragmented | Missing |
| 7041at7088 | Complete | Missing |
| 7044at7088 | Complete | Missing |
| 7045at7088 | Complete | Missing |
| 7047at7088 | Complete | Missing |
| 7048at7088 | Complete | Fragmented |
| 7051at7088 | Complete | Missing |
| 7052at7088 | Complete | Missing |
| 7053at7088 | Complete | Missing |
| 7056at7088 | Complete | Missing |
| 7058at7088 | Complete | Missing |
| 705at7088 | Complete | Fragmented |
| 7060at7088 | Complete | Missing |
| 7062at7088 | Fragmented | Missing |
| 7064at7088 | Complete | Missing |
| 7067at7088 | Complete | Missing |
| 7070at7088 | Duplicated | Missing |
| 7071at7088 | Complete | Missing |
| 7081at7088 | Complete | Missing |
| 7082at7088 | Complete | Missing |
| 7084at7088 | Complete | Missing |
| 7087at7088 | Complete | Missing |
| 7088at7088 | Complete | Complete |
| 7089at7088 | Complete | Missing |
| 708at7088 | Complete | Missing |
| 7090at7088 | Missing | Missing |
| 7092at7088 | Complete | Missing |
| 7097at7088 | Complete | Missing |
| 70at7088 | Complete | Missing |
| 7101at7088 | Complete | Missing |
| 7106at7088 | Duplicated | Missing |
| 7108at7088 | Complete | Missing |
| 7112at7088 | Complete | Complete |
| 7114at7088 | Complete | Missing |
| 7115at7088 | Complete | Missing |
| 7117at7088 | Complete | Fragmented |
| 7118at7088 | Complete | Missing |
| 7119at7088 | Complete | Missing |
| 711at7088 | Complete | Missing |
| 7122at7088 | Complete | Missing |
| 7123at7088 | Complete | Missing |
| 7125at7088 | Duplicated | Missing |
| 7126at7088 | Complete | Missing |
| 7128at7088 | Complete | Missing |
| 7129at7088 | Complete | Missing |
| 7133at7088 | Duplicated | Missing |
| 713at7088 | Complete | Missing |
| 7142at7088 | Complete | Missing |
| 7146at7088 | Complete | Missing |
| 7147at7088 | Complete | Missing |
| 714at7088 | Complete | Missing |
| 7153at7088 | Complete | Missing |
| 7154at7088 | Duplicated | Missing |
| 7156at7088 | Complete | Complete |
| 7159at7088 | Complete | Missing |
| 7160at7088 | Complete | Missing |
| 7162at7088 | Complete | Missing |
| 7163at7088 | Complete | Missing |
| 7166at7088 | Complete | Missing |
| 716at7088 | Complete | Missing |
| 7170at7088 | Complete | Missing |
| 7172at7088 | Complete | Missing |
| 7176at7088 | Complete | Missing |
| 7177at7088 | Complete | Missing |
| 717at7088 | Complete | Missing |
| 7181at7088 | Missing | Missing |
| 7182at7088 | Complete | Missing |
| 7184at7088 | Complete | Missing |
| 7185at7088 | Complete | Missing |
| 7188at7088 | Complete | Missing |
| 718at7088 | Complete | Missing |
| 7192at7088 | Fragmented | Missing |
| 7194at7088 | Complete | Missing |
| 7199at7088 | Fragmented | Missing |
| 7200at7088 | Complete | Missing |
| 7201at7088 | Complete | Complete |
| 7204at7088 | Complete | Missing |
| 7205at7088 | Complete | Missing |
| 7206at7088 | Complete | Missing |
| 7207at7088 | Missing | Missing |
| 7219at7088 | Complete | Missing |
| 721at7088 | Complete | Missing |
| 7220at7088 | Complete | Missing |
| 7223at7088 | Missing | Missing |
| 7227at7088 | Missing | Missing |
| 7229at7088 | Duplicated | Missing |
| 7230at7088 | Complete | Missing |
| 7237at7088 | Duplicated | Missing |
| 7238at7088 | Missing | Missing |
| 723at7088 | Complete | Missing |
| 7246at7088 | Duplicated | Missing |
| 7248at7088 | Complete | Complete |
| 724at7088 | Complete | Missing |
| 7253at7088 | Complete | Missing |
| 7254at7088 | Complete | Missing |
| 7258at7088 | Duplicated | Complete |
| 7260at7088 | Complete | Missing |
| 7263at7088 | Complete | Missing |
| 7264at7088 | Complete | Missing |
| 7267at7088 | Complete | Missing |
| 7268at7088 | Complete | Missing |
| 7271at7088 | Complete | Missing |
| 7272at7088 | Complete | Missing |
| 7277at7088 | Duplicated | Missing |
| 7283at7088 | Complete | Missing |
| 7285at7088 | Complete | Missing |
| 7286at7088 | Complete | Missing |
| 7288at7088 | Complete | Missing |
| 7290at7088 | Duplicated | Missing |
| 7295at7088 | Complete | Missing |
| 7298at7088 | Complete | Missing |
| 7299at7088 | Complete | Missing |
| 72at7088 | Duplicated | Missing |
| 7301at7088 | Complete | Missing |
| 7303at7088 | Duplicated | Missing |
| 7307at7088 | Duplicated | Missing |
| 7308at7088 | Complete | Missing |
| 730at7088 | Complete | Missing |
| 7313at7088 | Duplicated | Missing |
| 7314at7088 | Complete | Missing |
| 731at7088 | Complete | Missing |
| 7324at7088 | Complete | Missing |
| 7326at7088 | Complete | Missing |
| 7331at7088 | Complete | Missing |
| 7332at7088 | Complete | Missing |
| 7335at7088 | Complete | Missing |
| 7336at7088 | Complete | Missing |
| 7342at7088 | Complete | Complete |
| 7348at7088 | Complete | Missing |
| 7350at7088 | Complete | Missing |
| 7354at7088 | Complete | Complete |
| 7355at7088 | Complete | Missing |
| 7356at7088 | Complete | Missing |
| 7357at7088 | Complete | Missing |
| 7360at7088 | Complete | Missing |
| 7361at7088 | Complete | Missing |
| 7363at7088 | Complete | Missing |
| 7370at7088 | Complete | Missing |
| 7371at7088 | Complete | Missing |
| 7376at7088 | Complete | Missing |
| 7377at7088 | Complete | Missing |
| 7378at7088 | Complete | Missing |
| 7379at7088 | Complete | Missing |
| 7380at7088 | Duplicated | Complete |
| 7382at7088 | Complete | Missing |
| 7385at7088 | Fragmented | Missing |
| 7392at7088 | Complete | Missing |
| 7395at7088 | Complete | Missing |
| 7397at7088 | Complete | Missing |
| 7400at7088 | Complete | Missing |
| 7406at7088 | Complete | Missing |
| 7409at7088 | Complete | Missing |
| 7410at7088 | Complete | Missing |
| 7415at7088 | Complete | Missing |
| 7416at7088 | Complete | Missing |
| 7419at7088 | Complete | Missing |
| 741at7088 | Complete | Missing |
| 7420at7088 | Complete | Missing |
| 7427at7088 | Duplicated | Missing |
| 7429at7088 | Complete | Missing |
| 742at7088 | Complete | Missing |
| 7430at7088 | Complete | Missing |
| 7431at7088 | Complete | Missing |
| 7432at7088 | Complete | Missing |
| 7435at7088 | Complete | Missing |
| 7436at7088 | Complete | Complete |
| 7442at7088 | Complete | Missing |
| 7446at7088 | Complete | Missing |
| 7447at7088 | Complete | Missing |
| 7449at7088 | Complete | Missing |
| 7454at7088 | Missing | Missing |
| 7456at7088 | Complete | Missing |
| 7459at7088 | Complete | Missing |
| 7461at7088 | Missing | Missing |
| 7465at7088 | Missing | Fragmented |
| 7466at7088 | Complete | Missing |
| 7471at7088 | Complete | Missing |
| 7472at7088 | Complete | Missing |
| 7473at7088 | Complete | Missing |
| 7475at7088 | Complete | Missing |
| 7476at7088 | Duplicated | Missing |
| 7478at7088 | Complete | Missing |
| 7479at7088 | Fragmented | Missing |
| 7481at7088 | Complete | Missing |
| 7492at7088 | Complete | Missing |
| 7494at7088 | Complete | Missing |
| 7495at7088 | Complete | Missing |
| 7497at7088 | Complete | Missing |
| 74at7088 | Complete | Missing |
| 7500at7088 | Fragmented | Missing |
| 7501at7088 | Complete | Missing |
| 7506at7088 | Complete | Missing |
| 7508at7088 | Missing | Missing |
| 7512at7088 | Complete | Duplicated |
| 7513at7088 | Complete | Missing |
| 7520at7088 | Missing | Missing |
| 7523at7088 | Complete | Complete |
| 7527at7088 | Complete | Missing |
| 752at7088 | Complete | Missing |
| 7530at7088 | Complete | Missing |
| 7544at7088 | Complete | Missing |
| 7550at7088 | Complete | Missing |
| 7555at7088 | Fragmented | Fragmented |
| 7556at7088 | Complete | Missing |
| 7559at7088 | Complete | Missing |
| 7560at7088 | Complete | Missing |
| 7561at7088 | Complete | Missing |
| 7566at7088 | Complete | Missing |
| 7567at7088 | Complete | Missing |
| 7568at7088 | Complete | Missing |
| 756at7088 | Missing | Missing |
| 7573at7088 | Complete | Missing |
| 7574at7088 | Complete | Missing |
| 7576at7088 | Complete | Missing |
| 7577at7088 | Complete | Missing |
| 757at7088 | Complete | Missing |
| 7584at7088 | Complete | Missing |
| 7586at7088 | Complete | Complete |
| 758at7088 | Complete | Missing |
| 7591at7088 | Complete | Missing |
| 7592at7088 | Complete | Missing |
| 7594at7088 | Complete | Missing |
| 7595at7088 | Complete | Missing |
| 7597at7088 | Missing | Missing |
| 7598at7088 | Complete | Missing |
| 7607at7088 | Complete | Missing |
| 7608at7088 | Complete | Missing |
| 7611at7088 | Complete | Fragmented |
| 7614at7088 | Complete | Missing |
| 7618at7088 | Complete | Missing |
| 761at7088 | Complete | Missing |
| 7622at7088 | Complete | Missing |
| 7627at7088 | Complete | Missing |
| 7629at7088 | Complete | Missing |
| 7633at7088 | Fragmented | Missing |
| 7635at7088 | Complete | Missing |
| 7642at7088 | Complete | Complete |
| 764at7088 | Complete | Missing |
| 7654at7088 | Complete | Missing |
| 7656at7088 | Duplicated | Missing |
| 7659at7088 | Complete | Missing |
| 765at7088 | Fragmented | Duplicated |
| 7664at7088 | Complete | Missing |
| 7667at7088 | Fragmented | Missing |
| 7668at7088 | Duplicated | Missing |
| 7669at7088 | Complete | Missing |
| 7670at7088 | Complete | Missing |
| 7672at7088 | Complete | Missing |
| 7674at7088 | Complete | Missing |
| 7675at7088 | Duplicated | Missing |
| 7676at7088 | Duplicated | Missing |
| 7678at7088 | Complete | Missing |
| 7681at7088 | Complete | Missing |
| 7682at7088 | Complete | Missing |
| 7690at7088 | Complete | Missing |
| 7696at7088 | Complete | Missing |
| 7698at7088 | Complete | Missing |
| 7699at7088 | Complete | Missing |
| 769at7088 | Complete | Missing |
| 7705at7088 | Complete | Missing |
| 7706at7088 | Fragmented | Missing |
| 7707at7088 | Complete | Missing |
| 7708at7088 | Complete | Missing |
| 7712at7088 | Complete | Missing |
| 7714at7088 | Missing | Missing |
| 7719at7088 | Complete | Missing |
| 7720at7088 | Complete | Missing |
| 7721at7088 | Complete | Missing |
| 7722at7088 | Complete | Missing |
| 7723at7088 | Complete | Complete |
| 7726at7088 | Complete | Missing |
| 7727at7088 | Duplicated | Missing |
| 7729at7088 | Missing | Missing |
| 772at7088 | Complete | Missing |
| 7731at7088 | Complete | Missing |
| 7732at7088 | Complete | Missing |
| 7737at7088 | Complete | Missing |
| 7738at7088 | Complete | Missing |
| 7742at7088 | Complete | Missing |
| 7744at7088 | Complete | Missing |
| 7749at7088 | Complete | Missing |
| 774at7088 | Complete | Fragmented |
| 7751at7088 | Complete | Missing |
| 7754at7088 | Complete | Missing |
| 7755at7088 | Fragmented | Fragmented |
| 775at7088 | Complete | Missing |
| 7763at7088 | Complete | Missing |
| 7765at7088 | Complete | Missing |
| 7768at7088 | Complete | Missing |
| 7770at7088 | Complete | Missing |
| 7772at7088 | Missing | Missing |
| 7773at7088 | Complete | Missing |
| 7774at7088 | Duplicated | Missing |
| 7777at7088 | Complete | Fragmented |
| 7778at7088 | Missing | Missing |
| 7783at7088 | Duplicated | Missing |
| 7787at7088 | Complete | Missing |
| 7789at7088 | Complete | Missing |
| 7790at7088 | Complete | Missing |
| 7791at7088 | Complete | Missing |
| 7792at7088 | Complete | Missing |
| 7797at7088 | Complete | Missing |
| 7798at7088 | Complete | Missing |
| 7805at7088 | Duplicated | Missing |
| 780at7088 | Complete | Missing |
| 7811at7088 | Complete | Missing |
| 7813at7088 | Complete | Missing |
| 7815at7088 | Complete | Missing |
| 7817at7088 | Missing | Missing |
| 7819at7088 | Complete | Missing |
| 781at7088 | Complete | Missing |
| 7821at7088 | Missing | Missing |
| 7823at7088 | Complete | Missing |
| 7829at7088 | Complete | Missing |
| 7830at7088 | Complete | Complete |
| 7833at7088 | Complete | Missing |
| 7837at7088 | Complete | Missing |
| 7838at7088 | Complete | Missing |
| 7847at7088 | Complete | Missing |
| 7853at7088 | Complete | Missing |
| 7854at7088 | Duplicated | Missing |
| 7855at7088 | Complete | Missing |
| 7856at7088 | Complete | Missing |
| 7857at7088 | Missing | Missing |
| 7858at7088 | Complete | Missing |
| 7860at7088 | Complete | Missing |
| 7862at7088 | Complete | Missing |
| 7869at7088 | Complete | Missing |
| 7871at7088 | Complete | Missing |
| 7876at7088 | Missing | Missing |
| 787at7088 | Complete | Missing |
| 7882at7088 | Complete | Missing |
| 7884at7088 | Complete | Missing |
| 7887at7088 | Duplicated | Missing |
| 7890at7088 | Complete | Missing |
| 7894at7088 | Complete | Missing |
| 7897at7088 | Complete | Missing |
| 7899at7088 | Complete | Missing |
| 78at7088 | Missing | Missing |
| 7900at7088 | Complete | Missing |
| 7901at7088 | Complete | Missing |
| 7902at7088 | Missing | Missing |
| 7904at7088 | Complete | Missing |
| 7905at7088 | Complete | Missing |
| 7909at7088 | Duplicated | Missing |
| 790at7088 | Complete | Missing |
| 7911at7088 | Complete | Missing |
| 7914at7088 | Complete | Missing |
| 7915at7088 | Complete | Missing |
| 7918at7088 | Fragmented | Missing |
| 7919at7088 | Duplicated | Missing |
| 791at7088 | Missing | Missing |
| 7920at7088 | Complete | Missing |
| 7921at7088 | Complete | Missing |
| 7926at7088 | Complete | Missing |
| 7928at7088 | Complete | Missing |
| 7932at7088 | Complete | Missing |
| 7933at7088 | Missing | Missing |
| 7935at7088 | Complete | Missing |
| 793at7088 | Complete | Missing |
| 7940at7088 | Complete | Missing |
| 7948at7088 | Complete | Missing |
| 794at7088 | Missing | Missing |
| 7952at7088 | Complete | Missing |
| 7953at7088 | Complete | Missing |
| 7956at7088 | Complete | Missing |
| 795at7088 | Complete | Missing |
| 7966at7088 | Missing | Missing |
| 7967at7088 | Complete | Missing |
| 7968at7088 | Complete | Missing |
| 796at7088 | Complete | Complete |
| 7970at7088 | Complete | Missing |
| 7972at7088 | Complete | Missing |
| 7975at7088 | Complete | Missing |
| 7976at7088 | Complete | Missing |
| 7978at7088 | Missing | Missing |
| 7980at7088 | Complete | Missing |
| 7984at7088 | Complete | Missing |
| 7986at7088 | Duplicated | Missing |
| 7987at7088 | Complete | Missing |
| 7988at7088 | Fragmented | Missing |
| 7989at7088 | Complete | Missing |
| 7990at7088 | Complete | Missing |
| 7991at7088 | Complete | Missing |
| 7993at7088 | Complete | Missing |
| 7994at7088 | Complete | Missing |
| 7996at7088 | Complete | Missing |
| 7997at7088 | Complete | Missing |
| 799at7088 | Complete | Missing |
| 8001at7088 | Duplicated | Missing |
| 8006at7088 | Complete | Missing |
| 8007at7088 | Complete | Missing |
| 8008at7088 | Complete | Missing |
| 8009at7088 | Missing | Missing |
| 800at7088 | Complete | Missing |
| 8010at7088 | Duplicated | Missing |
| 8011at7088 | Complete | Missing |
| 8012at7088 | Complete | Missing |
| 8013at7088 | Complete | Missing |
| 8015at7088 | Complete | Missing |
| 8019at7088 | Duplicated | Missing |
| 802at7088 | Duplicated | Missing |
| 8031at7088 | Duplicated | Missing |
| 8032at7088 | Complete | Missing |
| 8033at7088 | Complete | Duplicated |
| 8034at7088 | Complete | Missing |
| 8039at7088 | Complete | Missing |
| 8040at7088 | Complete | Missing |
| 8043at7088 | Missing | Fragmented |
| 8044at7088 | Complete | Missing |
| 8045at7088 | Complete | Missing |
| 8046at7088 | Complete | Missing |
| 8048at7088 | Complete | Missing |
| 8050at7088 | Complete | Missing |
| 8051at7088 | Complete | Missing |
| 8052at7088 | Complete | Missing |
| 8053at7088 | Complete | Missing |
| 8055at7088 | Complete | Missing |
| 8057at7088 | Complete | Missing |
| 805at7088 | Complete | Missing |
| 8065at7088 | Complete | Missing |
| 8067at7088 | Complete | Missing |
| 806at7088 | Complete | Missing |
| 8071at7088 | Complete | Missing |
| 8072at7088 | Complete | Missing |
| 8073at7088 | Complete | Missing |
| 8079at7088 | Complete | Missing |
| 8080at7088 | Complete | Missing |
| 8081at7088 | Complete | Missing |
| 8084at7088 | Complete | Missing |
| 8088at7088 | Complete | Missing |
| 8089at7088 | Complete | Missing |
| 8097at7088 | Missing | Missing |
| 8098at7088 | Complete | Missing |
| 8100at7088 | Complete | Missing |
| 8101at7088 | Complete | Missing |
| 8102at7088 | Complete | Missing |
| 8103at7088 | Duplicated | Missing |
| 8106at7088 | Complete | Missing |
| 8107at7088 | Complete | Missing |
| 8111at7088 | Complete | Missing |
| 8116at7088 | Complete | Missing |
| 8117at7088 | Complete | Missing |
| 8118at7088 | Complete | Missing |
| 8120at7088 | Complete | Missing |
| 8122at7088 | Complete | Missing |
| 8129at7088 | Complete | Missing |
| 8131at7088 | Complete | Missing |
| 8132at7088 | Complete | Missing |
| 8136at7088 | Complete | Missing |
| 8139at7088 | Missing | Missing |
| 8144at7088 | Complete | Missing |
| 8148at7088 | Complete | Complete |
| 8149at7088 | Complete | Missing |
| 8150at7088 | Complete | Missing |
| 8151at7088 | Complete | Complete |
| 8153at7088 | Complete | Missing |
| 8156at7088 | Duplicated | Missing |
| 8166at7088 | Complete | Missing |
| 8167at7088 | Complete | Missing |
| 8171at7088 | Complete | Missing |
| 8172at7088 | Missing | Missing |
| 8174at7088 | Complete | Missing |
| 8175at7088 | Complete | Missing |
| 8177at7088 | Complete | Missing |
| 817at7088 | Complete | Missing |
| 8180at7088 | Complete | Missing |
| 8182at7088 | Complete | Missing |
| 8183at7088 | Complete | Missing |
| 8185at7088 | Complete | Missing |
| 8186at7088 | Complete | Missing |
| 818at7088 | Complete | Missing |
| 8190at7088 | Complete | Missing |
| 8201at7088 | Complete | Missing |
| 8204at7088 | Fragmented | Missing |
| 8205at7088 | Complete | Missing |
| 8211at7088 | Complete | Missing |
| 8212at7088 | Complete | Missing |
| 8214at7088 | Complete | Missing |
| 8215at7088 | Complete | Missing |
| 8217at7088 | Complete | Missing |
| 8219at7088 | Missing | Missing |
| 821at7088 | Complete | Missing |
| 8222at7088 | Complete | Missing |
| 822at7088 | Complete | Missing |
| 8235at7088 | Complete | Missing |
| 8236at7088 | Complete | Duplicated |
| 8238at7088 | Complete | Missing |
| 8240at7088 | Duplicated | Missing |
| 8247at7088 | Complete | Missing |
| 8248at7088 | Complete | Missing |
| 8254at7088 | Complete | Missing |
| 8255at7088 | Complete | Missing |
| 8258at7088 | Complete | Missing |
| 8259at7088 | Complete | Missing |
| 8264at7088 | Missing | Fragmented |
| 826at7088 | Complete | Missing |
| 8270at7088 | Fragmented | Missing |
| 8271at7088 | Complete | Missing |
| 8273at7088 | Complete | Missing |
| 8274at7088 | Fragmented | Missing |
| 8275at7088 | Complete | Missing |
| 8276at7088 | Complete | Missing |
| 8277at7088 | Complete | Missing |
| 8280at7088 | Complete | Missing |
| 8282at7088 | Complete | Missing |
| 8286at7088 | Complete | Missing |
| 828at7088 | Missing | Missing |
| 8298at7088 | Missing | Missing |
| 829at7088 | Complete | Complete |
| 8300at7088 | Complete | Missing |
| 8301at7088 | Complete | Missing |
| 8306at7088 | Duplicated | Missing |
| 8310at7088 | Complete | Missing |
| 8312at7088 | Complete | Missing |
| 8313at7088 | Complete | Missing |
| 8314at7088 | Complete | Missing |
| 8317at7088 | Missing | Missing |
| 8323at7088 | Complete | Missing |
| 8324at7088 | Complete | Missing |
| 8327at7088 | Complete | Missing |
| 8340at7088 | Complete | Missing |
| 8342at7088 | Complete | Missing |
| 8343at7088 | Complete | Missing |
| 8344at7088 | Complete | Missing |
| 8346at7088 | Duplicated | Missing |
| 8352at7088 | Complete | Missing |
| 8354at7088 | Missing | Missing |
| 8356at7088 | Missing | Missing |
| 8359at7088 | Complete | Missing |
| 835at7088 | Complete | Missing |
| 8365at7088 | Complete | Missing |
| 8367at7088 | Fragmented | Missing |
| 8370at7088 | Complete | Missing |
| 8371at7088 | Complete | Missing |
| 8372at7088 | Complete | Missing |
| 8373at7088 | Complete | Missing |
| 8375at7088 | Missing | Complete |
| 8377at7088 | Complete | Fragmented |
| 8380at7088 | Complete | Missing |
| 8384at7088 | Complete | Missing |
| 8385at7088 | Complete | Missing |
| 8386at7088 | Fragmented | Missing |
| 8388at7088 | Complete | Missing |
| 8392at7088 | Complete | Missing |
| 8393at7088 | Complete | Missing |
| 8394at7088 | Complete | Missing |
| 8396at7088 | Complete | Missing |
| 839at7088 | Complete | Missing |
| 83at7088 | Complete | Missing |
| 8400at7088 | Complete | Missing |
| 8402at7088 | Complete | Missing |
| 8403at7088 | Complete | Missing |
| 8406at7088 | Complete | Missing |
| 8408at7088 | Complete | Missing |
| 840at7088 | Complete | Missing |
| 8416at7088 | Complete | Missing |
| 8418at7088 | Complete | Missing |
| 841at7088 | Complete | Missing |
| 8420at7088 | Complete | Missing |
| 8426at7088 | Complete | Missing |
| 8427at7088 | Duplicated | Missing |
| 8432at7088 | Missing | Missing |
| 8438at7088 | Missing | Fragmented |
| 8440at7088 | Complete | Missing |
| 8441at7088 | Complete | Missing |
| 8443at7088 | Duplicated | Missing |
| 844at7088 | Complete | Missing |
| 8453at7088 | Fragmented | Missing |
| 845at7088 | Complete | Missing |
| 8464at7088 | Complete | Missing |
| 8465at7088 | Complete | Missing |
| 8467at7088 | Missing | Missing |
| 8469at7088 | Fragmented | Missing |
| 8471at7088 | Complete | Missing |
| 8476at7088 | Complete | Missing |
| 8477at7088 | Missing | Missing |
| 8479at7088 | Duplicated | Missing |
| 8483at7088 | Missing | Missing |
| 8484at7088 | Complete | Missing |
| 8487at7088 | Complete | Missing |
| 8491at7088 | Complete | Missing |
| 8492at7088 | Duplicated | Missing |
| 8493at7088 | Fragmented | Missing |
| 849at7088 | Complete | Fragmented |
| 84at7088 | Duplicated | Missing |
| 8504at7088 | Complete | Missing |
| 8509at7088 | Complete | Missing |
| 8514at7088 | Complete | Missing |
| 8515at7088 | Complete | Missing |
| 8517at7088 | Complete | Missing |
| 851at7088 | Complete | Missing |
| 8521at7088 | Complete | Missing |
| 8522at7088 | Complete | Missing |
| 8524at7088 | Complete | Missing |
| 8529at7088 | Complete | Missing |
| 8536at7088 | Complete | Missing |
| 853at7088 | Complete | Fragmented |
| 8549at7088 | Complete | Missing |
| 8550at7088 | Fragmented | Fragmented |
| 8553at7088 | Complete | Missing |
| 8554at7088 | Duplicated | Missing |
| 8559at7088 | Duplicated | Missing |
| 8564at7088 | Complete | Missing |
| 8570at7088 | Complete | Missing |
| 8571at7088 | Complete | Missing |
| 8572at7088 | Complete | Missing |
| 8578at7088 | Complete | Complete |
| 8579at7088 | Complete | Missing |
| 8582at7088 | Complete | Missing |
| 8584at7088 | Complete | Missing |
| 8585at7088 | Duplicated | Complete |
| 8586at7088 | Complete | Missing |
| 8587at7088 | Complete | Missing |
| 8591at7088 | Complete | Missing |
| 8595at7088 | Complete | Missing |
| 8596at7088 | Duplicated | Missing |
| 8605at7088 | Complete | Missing |
| 8607at7088 | Complete | Missing |
| 860at7088 | Complete | Missing |
| 8611at7088 | Complete | Missing |
| 8612at7088 | Complete | Missing |
| 8613at7088 | Duplicated | Missing |
| 8615at7088 | Complete | Missing |
| 8618at7088 | Fragmented | Complete |
| 861at7088 | Duplicated | Missing |
| 8620at7088 | Complete | Missing |
| 8625at7088 | Complete | Missing |
| 8628at7088 | Complete | Missing |
| 8630at7088 | Complete | Missing |
| 8633at7088 | Complete | Missing |
| 8635at7088 | Complete | Missing |
| 8636at7088 | Complete | Missing |
| 8637at7088 | Complete | Missing |
| 8638at7088 | Complete | Missing |
| 8642at7088 | Complete | Missing |
| 8643at7088 | Duplicated | Missing |
| 8644at7088 | Complete | Missing |
| 8650at7088 | Complete | Missing |
| 8652at7088 | Complete | Missing |
| 8653at7088 | Complete | Missing |
| 8659at7088 | Complete | Missing |
| 865at7088 | Complete | Missing |
| 8665at7088 | Complete | Missing |
| 8667at7088 | Complete | Missing |
| 8669at7088 | Complete | Missing |
| 866at7088 | Complete | Missing |
| 8672at7088 | Complete | Missing |
| 8673at7088 | Complete | Missing |
| 867at7088 | Complete | Missing |
| 8685at7088 | Duplicated | Missing |
| 8691at7088 | Complete | Missing |
| 8694at7088 | Missing | Missing |
| 8696at7088 | Complete | Missing |
| 8698at7088 | Missing | Missing |
| 86at7088 | Complete | Missing |
| 8700at7088 | Fragmented | Missing |
| 8703at7088 | Complete | Missing |
| 8708at7088 | Complete | Missing |
| 8709at7088 | Complete | Missing |
| 8710at7088 | Missing | Fragmented |
| 8711at7088 | Complete | Missing |
| 8712at7088 | Fragmented | Missing |
| 8715at7088 | Complete | Missing |
| 8716at7088 | Complete | Fragmented |
| 8718at7088 | Duplicated | Missing |
| 8719at7088 | Complete | Missing |
| 871at7088 | Complete | Fragmented |
| 8724at7088 | Complete | Missing |
| 8732at7088 | Complete | Missing |
| 8733at7088 | Complete | Missing |
| 8735at7088 | Complete | Missing |
| 8738at7088 | Complete | Missing |
| 8739at7088 | Complete | Missing |
| 8742at7088 | Complete | Missing |
| 8745at7088 | Complete | Missing |
| 8751at7088 | Complete | Missing |
| 8753at7088 | Complete | Missing |
| 8757at7088 | Complete | Missing |
| 875at7088 | Complete | Missing |
| 8760at7088 | Complete | Missing |
| 8763at7088 | Complete | Missing |
| 8764at7088 | Complete | Missing |
| 8767at7088 | Fragmented | Missing |
| 8769at7088 | Complete | Missing |
| 8770at7088 | Complete | Missing |
| 8777at7088 | Complete | Missing |
| 8778at7088 | Complete | Missing |
| 8779at7088 | Complete | Missing |
| 877at7088 | Complete | Missing |
| 8781at7088 | Complete | Missing |
| 8786at7088 | Complete | Fragmented |
| 8787at7088 | Complete | Missing |
| 8789at7088 | Complete | Missing |
| 8793at7088 | Complete | Missing |
| 8794at7088 | Complete | Missing |
| 8798at7088 | Complete | Missing |
| 879at7088 | Complete | Missing |
| 8804at7088 | Duplicated | Missing |
| 8806at7088 | Complete | Missing |
| 8807at7088 | Complete | Missing |
| 8809at7088 | Complete | Missing |
| 8811at7088 | Complete | Missing |
| 8812at7088 | Complete | Missing |
| 8817at7088 | Complete | Missing |
| 8818at7088 | Complete | Missing |
| 8819at7088 | Complete | Missing |
| 8820at7088 | Complete | Missing |
| 8829at7088 | Missing | Missing |
| 8834at7088 | Complete | Missing |
| 883at7088 | Complete | Missing |
| 8841at7088 | Complete | Missing |
| 8842at7088 | Complete | Missing |
| 8846at7088 | Complete | Missing |
| 8849at7088 | Complete | Missing |
| 8854at7088 | Complete | Missing |
| 8859at7088 | Complete | Complete |
| 885at7088 | Complete | Missing |
| 8860at7088 | Complete | Missing |
| 8861at7088 | Complete | Missing |
| 8862at7088 | Complete | Missing |
| 8863at7088 | Complete | Missing |
| 8864at7088 | Complete | Missing |
| 886at7088 | Complete | Missing |
| 8873at7088 | Complete | Missing |
| 8874at7088 | Missing | Missing |
| 8875at7088 | Complete | Missing |
| 8877at7088 | Complete | Missing |
| 8881at7088 | Complete | Complete |
| 8882at7088 | Complete | Missing |
| 8885at7088 | Duplicated | Missing |
| 8886at7088 | Duplicated | Missing |
| 8890at7088 | Complete | Missing |
| 8895at7088 | Complete | Missing |
| 8896at7088 | Complete | Missing |
| 8897at7088 | Complete | Missing |
| 8898at7088 | Complete | Missing |
| 88at7088 | Complete | Missing |
| 8900at7088 | Fragmented | Missing |
| 8901at7088 | Complete | Missing |
| 8902at7088 | Complete | Missing |
| 8908at7088 | Missing | Missing |
| 890at7088 | Complete | Missing |
| 8912at7088 | Complete | Missing |
| 8914at7088 | Complete | Missing |
| 8915at7088 | Complete | Missing |
| 8916at7088 | Complete | Missing |
| 8920at7088 | Complete | Missing |
| 8923at7088 | Complete | Missing |
| 8927at7088 | Complete | Missing |
| 8928at7088 | Complete | Missing |
| 8930at7088 | Complete | Missing |
| 8931at7088 | Complete | Missing |
| 8934at7088 | Complete | Missing |
| 8937at7088 | Complete | Missing |
| 893at7088 | Complete | Missing |
| 8946at7088 | Complete | Missing |
| 8953at7088 | Complete | Missing |
| 8954at7088 | Complete | Missing |
| 8955at7088 | Complete | Missing |
| 8957at7088 | Complete | Missing |
| 895at7088 | Complete | Missing |
| 8962at7088 | Complete | Missing |
| 8967at7088 | Duplicated | Missing |
| 8971at7088 | Complete | Fragmented |
| 8973at7088 | Complete | Complete |
| 8974at7088 | Complete | Missing |
| 8976at7088 | Complete | Missing |
| 8978at7088 | Complete | Missing |
| 897at7088 | Complete | Missing |
| 8981at7088 | Fragmented | Missing |
| 8982at7088 | Complete | Missing |
| 8984at7088 | Fragmented | Missing |
| 8986at7088 | Missing | Missing |
| 8988at7088 | Complete | Missing |
| 8990at7088 | Complete | Missing |
| 8993at7088 | Complete | Missing |
| 8994at7088 | Complete | Missing |
| 899at7088 | Complete | Missing |
| 9004at7088 | Duplicated | Missing |
| 9006at7088 | Complete | Missing |
| 9012at7088 | Complete | Missing |
| 901at7088 | Complete | Missing |
| 9020at7088 | Complete | Missing |
| 9021at7088 | Duplicated | Missing |
| 9025at7088 | Complete | Missing |
| 9031at7088 | Complete | Missing |
| 9032at7088 | Complete | Fragmented |
| 9036at7088 | Missing | Missing |
| 903at7088 | Complete | Missing |
| 9044at7088 | Complete | Missing |
| 9047at7088 | Complete | Missing |
| 9052at7088 | Duplicated | Missing |
| 9053at7088 | Complete | Missing |
| 9055at7088 | Complete | Missing |
| 9056at7088 | Complete | Missing |
| 9060at7088 | Complete | Fragmented |
| 9061at7088 | Complete | Missing |
| 9062at7088 | Complete | Missing |
| 9067at7088 | Complete | Missing |
| 9068at7088 | Complete | Missing |
| 9069at7088 | Complete | Missing |
| 9074at7088 | Complete | Missing |
| 9081at7088 | Complete | Missing |
| 9088at7088 | Complete | Missing |
| 9098at7088 | Complete | Missing |
| 9099at7088 | Complete | Missing |
| 909at7088 | Complete | Missing |
| 9101at7088 | Complete | Missing |
| 9102at7088 | Complete | Missing |
| 9103at7088 | Complete | Missing |
| 9106at7088 | Complete | Missing |
| 9108at7088 | Complete | Missing |
| 9113at7088 | Complete | Missing |
| 9117at7088 | Complete | Missing |
| 9119at7088 | Complete | Missing |
| 911at7088 | Complete | Complete |
| 9121at7088 | Complete | Missing |
| 9123at7088 | Complete | Missing |
| 9124at7088 | Complete | Missing |
| 9125at7088 | Complete | Missing |
| 9127at7088 | Complete | Fragmented |
| 912at7088 | Complete | Missing |
| 9131at7088 | Complete | Missing |
| 9134at7088 | Complete | Missing |
| 9135at7088 | Complete | Missing |
| 9136at7088 | Missing | Missing |
| 913at7088 | Complete | Missing |
| 9142at7088 | Complete | Missing |
| 9144at7088 | Complete | Missing |
| 9145at7088 | Complete | Missing |
| 9146at7088 | Complete | Missing |
| 9149at7088 | Complete | Missing |
| 9150at7088 | Fragmented | Missing |
| 9155at7088 | Complete | Missing |
| 9162at7088 | Complete | Missing |
| 9169at7088 | Complete | Missing |
| 9171at7088 | Complete | Missing |
| 9176at7088 | Complete | Missing |
| 9179at7088 | Complete | Missing |
| 917at7088 | Complete | Missing |
| 9180at7088 | Complete | Missing |
| 9182at7088 | Complete | Missing |
| 9185at7088 | Complete | Missing |
| 9187at7088 | Complete | Missing |
| 9188at7088 | Complete | Missing |
| 9191at7088 | Complete | Missing |
| 9194at7088 | Complete | Missing |
| 9198at7088 | Complete | Missing |
| 9200at7088 | Missing | Missing |
| 9205at7088 | Complete | Missing |
| 9209at7088 | Complete | Missing |
| 9210at7088 | Missing | Missing |
| 9211at7088 | Complete | Missing |
| 9213at7088 | Complete | Missing |
| 9214at7088 | Complete | Missing |
| 9216at7088 | Missing | Missing |
| 9217at7088 | Complete | Missing |
| 9218at7088 | Complete | Missing |
| 9222at7088 | Complete | Missing |
| 9224at7088 | Complete | Missing |
| 9232at7088 | Complete | Missing |
| 9236at7088 | Duplicated | Missing |
| 9237at7088 | Complete | Missing |
| 9240at7088 | Complete | Missing |
| 9241at7088 | Complete | Missing |
| 9243at7088 | Complete | Missing |
| 9246at7088 | Complete | Missing |
| 924at7088 | Complete | Missing |
| 9252at7088 | Complete | Fragmented |
| 9254at7088 | Complete | Missing |
| 9258at7088 | Missing | Missing |
| 9260at7088 | Complete | Missing |
| 9265at7088 | Complete | Missing |
| 9271at7088 | Complete | Missing |
| 9272at7088 | Complete | Missing |
| 9274at7088 | Complete | Missing |
| 9275at7088 | Complete | Missing |
| 9276at7088 | Complete | Missing |
| 9278at7088 | Complete | Missing |
| 927at7088 | Complete | Missing |
| 9282at7088 | Complete | Missing |
| 9285at7088 | Complete | Missing |
| 9286at7088 | Duplicated | Missing |
| 9288at7088 | Complete | Missing |
| 9289at7088 | Complete | Missing |
| 9290at7088 | Missing | Missing |
| 9292at7088 | Complete | Missing |
| 9293at7088 | Fragmented | Missing |
| 9294at7088 | Complete | Missing |
| 9297at7088 | Complete | Missing |
| 929at7088 | Duplicated | Missing |
| 9301at7088 | Complete | Missing |
| 9304at7088 | Complete | Missing |
| 9309at7088 | Complete | Missing |
| 9316at7088 | Complete | Missing |
| 9319at7088 | Complete | Missing |
| 9322at7088 | Complete | Missing |
| 9326at7088 | Missing | Missing |
| 9331at7088 | Duplicated | Missing |
| 9332at7088 | Duplicated | Missing |
| 9333at7088 | Complete | Missing |
| 9336at7088 | Complete | Missing |
| 9337at7088 | Complete | Missing |
| 9338at7088 | Complete | Missing |
| 9339at7088 | Complete | Missing |
| 933at7088 | Missing | Missing |
| 9343at7088 | Complete | Missing |
| 9345at7088 | Complete | Missing |
| 934at7088 | Complete | Missing |
| 9360at7088 | Complete | Missing |
| 9362at7088 | Duplicated | Missing |
| 9364at7088 | Complete | Missing |
| 9365at7088 | Complete | Missing |
| 9366at7088 | Complete | Missing |
| 9367at7088 | Missing | Missing |
| 9374at7088 | Complete | Missing |
| 9380at7088 | Complete | Missing |
| 9381at7088 | Complete | Missing |
| 9386at7088 | Duplicated | Missing |
| 9388at7088 | Complete | Missing |
| 9389at7088 | Complete | Missing |
| 938at7088 | Complete | Missing |
| 9392at7088 | Complete | Missing |
| 9395at7088 | Complete | Missing |
| 9397at7088 | Complete | Missing |
| 9401at7088 | Missing | Missing |
| 9402at7088 | Complete | Fragmented |
| 9409at7088 | Missing | Missing |
| 9413at7088 | Complete | Missing |
| 9417at7088 | Complete | Missing |
| 9419at7088 | Complete | Missing |
| 9430at7088 | Duplicated | Missing |
| 9431at7088 | Complete | Missing |
| 9432at7088 | Complete | Missing |
| 9435at7088 | Complete | Missing |
| 9436at7088 | Complete | Missing |
| 9439at7088 | Complete | Missing |
| 9441at7088 | Complete | Missing |
| 9442at7088 | Complete | Missing |
| 9443at7088 | Complete | Missing |
| 9448at7088 | Complete | Missing |
| 944at7088 | Duplicated | Missing |
| 9451at7088 | Complete | Missing |
| 9452at7088 | Complete | Missing |
| 9458at7088 | Complete | Missing |
| 9465at7088 | Complete | Missing |
| 9468at7088 | Complete | Missing |
| 946at7088 | Complete | Missing |
| 9470at7088 | Complete | Complete |
| 9472at7088 | Complete | Missing |
| 9475at7088 | Complete | Missing |
| 9477at7088 | Complete | Complete |
| 947at7088 | Complete | Missing |
| 9480at7088 | Complete | Missing |
| 9481at7088 | Complete | Missing |
| 9492at7088 | Complete | Missing |
| 9494at7088 | Duplicated | Missing |
| 949at7088 | Complete | Missing |
| 9503at7088 | Complete | Missing |
| 9505at7088 | Complete | Missing |
| 9506at7088 | Duplicated | Missing |
| 950at7088 | Complete | Missing |
| 9511at7088 | Complete | Missing |
| 9513at7088 | Fragmented | Missing |
| 9515at7088 | Complete | Missing |
| 9517at7088 | Complete | Missing |
| 9518at7088 | Complete | Missing |
| 9519at7088 | Complete | Missing |
| 951at7088 | Complete | Missing |
| 9521at7088 | Complete | Missing |
| 9524at7088 | Duplicated | Missing |
| 9526at7088 | Complete | Missing |
| 9527at7088 | Complete | Fragmented |
| 9528at7088 | Duplicated | Missing |
| 9533at7088 | Complete | Missing |
| 9534at7088 | Complete | Missing |
| 9535at7088 | Complete | Missing |
| 9543at7088 | Complete | Missing |
| 9544at7088 | Missing | Missing |
| 9548at7088 | Complete | Missing |
| 9549at7088 | Duplicated | Missing |
| 954at7088 | Complete | Fragmented |
| 9554at7088 | Complete | Missing |
| 9556at7088 | Complete | Missing |
| 9559at7088 | Missing | Missing |
| 9563at7088 | Complete | Fragmented |
| 9564at7088 | Fragmented | Fragmented |
| 9567at7088 | Complete | Missing |
| 9568at7088 | Complete | Missing |
| 9571at7088 | Complete | Missing |
| 9575at7088 | Complete | Missing |
| 9576at7088 | Duplicated | Missing |
| 9577at7088 | Complete | Missing |
| 9580at7088 | Missing | Missing |
| 9582at7088 | Complete | Missing |
| 9595at7088 | Complete | Missing |
| 9597at7088 | Missing | Missing |
| 9598at7088 | Missing | Missing |
| 9601at7088 | Complete | Missing |
| 9603at7088 | Duplicated | Missing |
| 9604at7088 | Complete | Missing |
| 9609at7088 | Complete | Missing |
| 9611at7088 | Complete | Missing |
| 9618at7088 | Complete | Missing |
| 9619at7088 | Complete | Missing |
| 961at7088 | Complete | Complete |
| 9623at7088 | Complete | Missing |
| 9628at7088 | Duplicated | Missing |
| 962at7088 | Complete | Missing |
| 9630at7088 | Complete | Missing |
| 9631at7088 | Complete | Missing |
| 9638at7088 | Complete | Missing |
| 963at7088 | Complete | Missing |
| 9640at7088 | Complete | Missing |
| 9647at7088 | Complete | Missing |
| 9648at7088 | Complete | Missing |
| 964at7088 | Complete | Missing |
| 9651at7088 | Complete | Missing |
| 9655at7088 | Complete | Missing |
| 9656at7088 | Duplicated | Missing |
| 9657at7088 | Complete | Missing |
| 9662at7088 | Complete | Missing |
| 9663at7088 | Complete | Missing |
| 9665at7088 | Complete | Missing |
| 9666at7088 | Complete | Missing |
| 9669at7088 | Complete | Complete |
| 966at7088 | Complete | Missing |
| 9680at7088 | Complete | Missing |
| 9682at7088 | Complete | Missing |
| 9688at7088 | Complete | Missing |
| 9689at7088 | Complete | Missing |
| 968at7088 | Complete | Missing |
| 9693at7088 | Complete | Missing |
| 9694at7088 | Complete | Complete |
| 9695at7088 | Duplicated | Missing |
| 9698at7088 | Complete | Missing |
| 9699at7088 | Complete | Missing |
| 96at7088 | Complete | Missing |
| 9701at7088 | Complete | Missing |
| 9709at7088 | Fragmented | Missing |
| 9710at7088 | Complete | Missing |
| 9711at7088 | Complete | Missing |
| 9713at7088 | Complete | Missing |
| 9715at7088 | Complete | Missing |
| 9721at7088 | Complete | Missing |
| 9722at7088 | Complete | Missing |
| 9724at7088 | Complete | Fragmented |
| 9729at7088 | Complete | Missing |
| 972at7088 | Complete | Missing |
| 9730at7088 | Complete | Missing |
| 9731at7088 | Complete | Missing |
| 9737at7088 | Missing | Missing |
| 9739at7088 | Complete | Missing |
| 9745at7088 | Complete | Missing |
| 9748at7088 | Complete | Missing |
| 9749at7088 | Complete | Missing |
| 974at7088 | Complete | Missing |
| 9750at7088 | Complete | Missing |
| 9752at7088 | Complete | Missing |
| 9755at7088 | Missing | Missing |
| 9759at7088 | Complete | Missing |
| 975at7088 | Complete | Missing |
| 9760at7088 | Duplicated | Missing |
| 9765at7088 | Complete | Missing |
| 9768at7088 | Complete | Missing |
| 9770at7088 | Complete | Missing |
| 9775at7088 | Complete | Missing |
| 9777at7088 | Complete | Missing |
| 9780at7088 | Complete | Fragmented |
| 9781at7088 | Duplicated | Missing |
| 9782at7088 | Complete | Fragmented |
| 9784at7088 | Complete | Missing |
| 9785at7088 | Missing | Missing |
| 9788at7088 | Complete | Complete |
| 978at7088 | Complete | Fragmented |
| 9790at7088 | Complete | Missing |
| 9791at7088 | Complete | Missing |
| 9793at7088 | Missing | Missing |
| 979at7088 | Duplicated | Missing |
| 9800at7088 | Complete | Missing |
| 9802at7088 | Complete | Missing |
| 9803at7088 | Complete | Missing |
| 9807at7088 | Complete | Missing |
| 9809at7088 | Complete | Complete |
| 980at7088 | Complete | Missing |
| 9810at7088 | Complete | Missing |
| 9817at7088 | Complete | Missing |
| 9818at7088 | Complete | Missing |
| 9819at7088 | Complete | Missing |
| 981at7088 | Complete | Missing |
| 9820at7088 | Complete | Missing |
| 9824at7088 | Complete | Missing |
| 9825at7088 | Complete | Missing |
| 9828at7088 | Complete | Missing |
| 982at7088 | Complete | Missing |
| 9830at7088 | Complete | Missing |
| 9835at7088 | Complete | Missing |
| 9836at7088 | Complete | Missing |
| 9837at7088 | Complete | Missing |
| 9839at7088 | Complete | Missing |
| 983at7088 | Complete | Missing |
| 9840at7088 | Complete | Missing |
| 9846at7088 | Complete | Fragmented |
| 9847at7088 | Fragmented | Missing |
| 9848at7088 | Complete | Missing |
| 984at7088 | Complete | Missing |
| 9850at7088 | Missing | Missing |
| 9860at7088 | Complete | Missing |
| 9861at7088 | Complete | Missing |
| 9862at7088 | Complete | Missing |
| 9864at7088 | Complete | Missing |
| 9865at7088 | Complete | Missing |
| 9866at7088 | Complete | Missing |
| 9867at7088 | Complete | Missing |
| 986at7088 | Complete | Missing |
| 9871at7088 | Complete | Missing |
| 9874at7088 | Complete | Missing |
| 9878at7088 | Complete | Missing |
| 9881at7088 | Complete | Missing |
| 9884at7088 | Missing | Missing |
| 9885at7088 | Complete | Missing |
| 988at7088 | Complete | Missing |
| 9890at7088 | Duplicated | Missing |
| 9891at7088 | Complete | Missing |
| 9896at7088 | Complete | Missing |
| 9897at7088 | Complete | Missing |
| 9899at7088 | Complete | Missing |
| 989at7088 | Complete | Missing |
| 9900at7088 | Complete | Missing |
| 9903at7088 | Complete | Missing |
| 9904at7088 | Complete | Complete |
| 9911at7088 | Complete | Missing |
| 9914at7088 | Complete | Missing |
| 9918at7088 | Complete | Missing |
| 9926at7088 | Complete | Missing |
| 9927at7088 | Complete | Missing |
| 992at7088 | Complete | Missing |
| 9931at7088 | Complete | Missing |
| 9935at7088 | Complete | Missing |
| 993at7088 | Complete | Missing |
| 9943at7088 | Complete | Missing |
| 9947at7088 | Complete | Missing |
| 9949at7088 | Complete | Missing |
| 994at7088 | Complete | Missing |
| 9952at7088 | Complete | Missing |
| 9956at7088 | Duplicated | Missing |
| 9957at7088 | Complete | Missing |
| 9960at7088 | Complete | Missing |
| 9962at7088 | Complete | Missing |
| 9965at7088 | Complete | Missing |
| 9967at7088 | Complete | Missing |
| 9976at7088 | Complete | Missing |
| 9977at7088 | Complete | Missing |
| 9978at7088 | Complete | Missing |
| 9981at7088 | Complete | Missing |
| 9985at7088 | Complete | Missing |
| 9987at7088 | Complete | Complete |
| 998at7088 | Fragmented | Missing |
| 9990at7088 | Complete | Missing |
| 9992at7088 | Complete | Missing |
| 9999at7088 | Complete | Missing |
| 999at7088 | Complete | Missing |
