# Supplementary material for: Is the extreme within-population genome size variation real in Spodoptera frugiperda?
Source: PLoS One. 2025 Sep 30;20(9):e0332711. doi: 10.1371/journal.pone.0332711 (PMC12483198; doi:10.1371/journal.pone.0332711)
Supplement: S3 Table — (DOCX) [file pone.0332711.s003.docx]

Table S3. The genes showing 100% protein sequence identity between reference genome assemblies and non-reference sequences.

| non-refernce | reference |
| --- | --- |
| augustus-A4_jcf7180001994341-processed-gene-0.0-mRNA-1 | SFR08232 |
| maker-C9_jcf7180002135347-augustus-gene-0.1-mRNA-1 | SFR19601 |
| augustus-B3_jcf7180000805897-processed-gene-0.0-mRNA-1 | SFR08923 |
| maker-A6_jcf7180001618336-augustus-gene-0.1-mRNA-1 | SFR08430 |
| augustus-A3_jcf7180002059679-processed-gene-0.1-mRNA-1 | SFR03807 |
| augustus-B5_jcf7180001613801-processed-gene-0.3-mRNA-1 | SFR10847 |
| augustus-B5_jcf7180001613801-processed-gene-0.4-mRNA-1 | SFR10847 |
| maker-B5_jcf7180001718822-augustus-gene-0.0-mRNA-1 | SFR21448 |
| maker-B5_jcf7180001718822-augustus-gene-0.0-mRNA-1 | SFR21447 |
| maker-B5_jcf7180001718822-augustus-gene-0.0-mRNA-1 | SFR20896 |
| maker-B5_jcf7180001718822-augustus-gene-0.0-mRNA-1 | SFR20895 |
| maker-A4_jcf7180002074457-augustus-gene-0.1-mRNA-1 | SFR09763 |
| augustus-C1_jcf7180001811820-processed-gene-0.0-mRNA-1 | SFR00586 |
| maker-KE-10_jcf7180003305229-augustus-gene-0.0-mRNA-1 | SFR22145 |
| augustus-B2_jcf7180001564838-processed-gene-0.0-mRNA-1 | SFR14533 |
| maker-B9_jcf7180001583917-augustus-gene-0.0-mRNA-1 | SFR21221 |
| maker-A10_jcf7180002077372-augustus-gene-0.0-mRNA-1 | SFR21474 |
| maker-D2_jcf7180002016344-augustus-gene-0.1-mRNA-1 | SFR01719 |
| maker-C10_jcf7180001635094-augustus-gene-0.1-mRNA-1 | SFR02538 |
| maker-C3_jcf7180001778625-augustus-gene-0.0-mRNA-1 | SFR07338 |
| augustus-A3_jcf7180002004556-processed-gene-0.2-mRNA-1 | SFR03221 |
| maker-C9_jcf7180001993378-augustus-gene-0.1-mRNA-1 | SFR18166 |
| augustus-A7_jcf7180002042323-processed-gene-0.0-mRNA-1 | SFR07531 |
| augustus-A9_jcf7180001982933-processed-gene-0.0-mRNA-1 | SFR01313 |
| maker-D3_jcf7180002087083-augustus-gene-0.0-mRNA-1 | SFR11322 |
| maker-A1_jcf7180001990518-augustus-gene-0.0-mRNA-1 | SFR06134 |
| maker-FJ01_3_jcf7180001536045-augustus-gene-0.0-mRNA-1 | SFR12899 |
| maker-B3_jcf7180000736447-augustus-gene-0.0-mRNA-1 | SFR19385 |
| maker-C6_jcf7180001993400-augustus-gene-0.1-mRNA-1 | SFR21098 |
| maker-KE-7_jcf7180002479513-augustus-gene-0.1-mRNA-1 | SFR20653 |
| maker-KE-7_jcf7180002479513-augustus-gene-0.1-mRNA-1 | SFR12362 |
| maker-C6_jcf7180001963932-augustus-gene-0.0-mRNA-1 | SFR22145 |
| maker-A2_jcf7180001803167-augustus-gene-0.1-mRNA-1 | SFR16217 |
| augustus-JC01_jcf7180001286708-processed-gene-0.0-mRNA-1 | SFR14500 |
| maker-GX03_jcf7180001368607-augustus-gene-0.0-mRNA-1 | SFR21221 |
| augustus-D2_jcf7180001924617-processed-gene-0.0-mRNA-1 | SFR18566 |
| maker-A6_jcf7180001729106-augustus-gene-0.0-mRNA-1 | SFR10969 |
| augustus-C9_jcf7180002016460-processed-gene-0.0-mRNA-1 | SFR02528 |
| maker-A9_jcf7180001832777-augustus-gene-0.1-mRNA-1 | SFR12145 |
| maker-A9_jcf7180001832777-augustus-gene-0.1-mRNA-1 | SFR18370 |
| augustus-C1_jcf7180001861354-processed-gene-0.0-mRNA-1 | SFR10356 |
| maker-B8_jcf7180001593364-augustus-gene-0.0-mRNA-1 | SFR10791 |
| maker-C2_jcf7180001739911-augustus-gene-0.0-mRNA-1 | SFR02538 |
| augustus-C9_jcf7180001958156-processed-gene-0.1-mRNA-1 | SFR20654 |
| augustus-C9_jcf7180001958156-processed-gene-0.1-mRNA-1 | SFR18369 |
| maker-C1_jcf7180001976972-augustus-gene-0.1-mRNA-1 | SFR13001 |
| maker-B2_jcf7180001594088-augustus-gene-0.1-mRNA-1 | SFR01994 |
| maker-B2_jcf7180001594088-augustus-gene-0.1-mRNA-1 | SFR03623 |
| maker-B4_jcf7180001636075-augustus-gene-0.0-mRNA-1 | SFR02215 |
| maker-A9_jcf7180001983355-augustus-gene-0.1-mRNA-1 | SFR00348 |
| maker-D2_jcf7180001904727-augustus-gene-0.0-mRNA-1 | SFR10791 |
| maker-MH03_jcf7180001600449-augustus-gene-0.0-mRNA-1 | SFR00369 |
| maker-B6_jcf7180001682025-augustus-gene-0.0-mRNA-1 | SFR00602 |
| maker-D8_jcf7180001941172-augustus-gene-0.0-mRNA-1 | SFR15293 |
| maker-B1_jcf7180001487607-augustus-gene-0.1-mRNA-1 | SFR15293 |
| maker-KE-7_jcf7180002538790-augustus-gene-0.0-mRNA-1 | SFR03369 |
| augustus-FAW5.2_jcf7180001529191-processed-gene-0.0-mRNA-1 | SFR05501 |
| maker-GX04_jcf7180001150051-augustus-gene-0.1-mRNA-1 | SFR11999 |
| maker-KE-4_jcf7180006946986-augustus-gene-0.1-mRNA-1 | SFR05615 |
| maker-C10_jcf7180001736870-augustus-gene-0.0-mRNA-1 | SFR07338 |
| augustus-ML3_jcf7180001827194-processed-gene-0.0-mRNA-1 | SFR08038 |
| augustus-A4_jcf7180002064132-processed-gene-0.0-mRNA-1 | SFR16637 |
| augustus-A10_jcf7180002275407-processed-gene-0.0-mRNA-1 | SFR06226 |
| maker-D1_jcf7180001980067-augustus-gene-0.0-mRNA-1 | SFR06620 |
| augustus-B6_jcf7180001598535-processed-gene-0.0-mRNA-1 | SFR17137 |
| augustus-D2_jcf7180001932096-processed-gene-0.0-mRNA-1 | SFR07531 |
| augustus-D1_jcf7180001962116-processed-gene-0.0-mRNA-1 | SFR05578 |
| augustus-D1_jcf7180001962116-processed-gene-0.0-mRNA-1 | SFR05575 |
| augustus-C6_jcf7180001984688-processed-gene-0.0-mRNA-1 | SFR10417 |
| maker-A7_jcf7180001869112-augustus-gene-0.0-mRNA-1 | SFR19848 |
| maker-D2_jcf7180001921213-augustus-gene-0.0-mRNA-1 | SFR12345 |
| maker-A5_jcf7180002029619-augustus-gene-0.1-mRNA-1 | SFR20166 |
| augustus-A3_jcf7180001944291-processed-gene-0.0-mRNA-1 | SFR00372 |
| maker-D6_jcf7180002052701-augustus-gene-0.0-mRNA-1 | SFR18166 |
| maker-A4_jcf7180002065213-augustus-gene-0.1-mRNA-1 | SFR08531 |
| augustus-KE-4_jcf7180006838181-processed-gene-0.0-mRNA-1 | SFR22145 |
| maker-B2_jcf7180001608844-augustus-gene-0.1-mRNA-1 | SFR17800 |
| maker-D3_jcf7180002134861-augustus-gene-0.0-mRNA-1 | SFR17571 |
| maker-D8_jcf7180002055439-augustus-gene-0.1-mRNA-1 | SFR02815 |
| maker-FJ01_2_jcf7180001284627-augustus-gene-0.0-mRNA-1 | SFR04200 |
| maker-D1_jcf7180002200088-augustus-gene-0.0-mRNA-1 | SFR16813 |
| maker-C1_jcf7180001826882-augustus-gene-0.0-mRNA-1 | SFR04295 |
| maker-LL06_jcf7180001303943-augustus-gene-0.0-mRNA-1 | SFR15449 |
| augustus-C3_jcf7180001947257-processed-gene-0.0-mRNA-1 | SFR19451 |
| maker-LL09_jcf7180001536298-augustus-gene-0.1-mRNA-1 | SFR03221 |
| maker-A3_jcf7180001984943-augustus-gene-0.1-mRNA-1 | SFR16119 |
| augustus-C9_jcf7180002143913-processed-gene-0.0-mRNA-1 | SFR19167 |
| maker-D2_jcf7180002033264-augustus-gene-0.1-mRNA-1 | SFR05261 |
| maker-D6_jcf7180002107623-augustus-gene-0.0-mRNA-1 | SFR08904 |
| maker-KE-9_jcf7180003257783-augustus-gene-0.0-mRNA-1 | SFR00460 |
| maker-ML3_jcf7180001817783-augustus-gene-0.0-mRNA-1 | SFR15449 |
| maker-A7_jcf7180001868704-augustus-gene-0.0-mRNA-1 | SFR20654 |
| maker-A7_jcf7180001868704-augustus-gene-0.0-mRNA-1 | SFR18369 |
| augustus-C8_jcf7180001809302-processed-gene-0.0-mRNA-1 | SFR01774 |
| augustus-C7_jcf7180002097425-processed-gene-0.0-mRNA-1 | SFR22189 |
| maker-D6_jcf7180002093872-augustus-gene-0.1-mRNA-1 | SFR06528 |
| maker-D7_jcf7180002000722-augustus-gene-0.1-mRNA-1 | SFR21851 |
| augustus-A8_jcf7180001987632-processed-gene-0.0-mRNA-1 | SFR04723 |
| maker-B9_jcf7180001565082-augustus-gene-0.0-mRNA-1 | SFR05626 |
| maker-BS02_jcf7180001549453-augustus-gene-0.1-mRNA-1 | SFR08989 |
| maker-B6_jcf7180001586955-augustus-gene-0.0-mRNA-1 | SFR20213 |
| maker-B5_jcf7180001584001-augustus-gene-0.1-mRNA-1 | SFR01329 |
| maker-B10_jcf7180001612488-augustus-gene-0.0-mRNA-1 | SFR12257 |
| maker-B6_jcf7180001568127-augustus-gene-0.1-mRNA-1 | SFR11202 |
| maker-A2_jcf7180001762947-augustus-gene-0.1-mRNA-1 | SFR10074 |
| maker-KE-4_jcf7180006717484-augustus-gene-0.1-mRNA-1 | SFR14353 |
| augustus-C1_jcf7180001946302-processed-gene-0.2-mRNA-1 | SFR16120 |
| maker-A4_jcf7180001990586-augustus-gene-0.0-mRNA-1 | SFR12257 |
| augustus-D5_jcf7180001826093-processed-gene-0.0-mRNA-1 | SFR11346 |
| augustus-C1_jcf7180001820286-processed-gene-0.0-mRNA-1 | SFR15256 |
| maker-A2_jcf7180001872447-augustus-gene-0.0-mRNA-1 | SFR18723 |
| augustus-B9_jcf7180001486072-processed-gene-0.0-mRNA-1 | SFR16493 |
| augustus-GX03_jcf7180001383401-processed-gene-0.0-mRNA-1 | SFR13510 |
| maker-MH01_jcf7180001449304-augustus-gene-0.1-mRNA-1 | SFR14286 |
| maker-A6_jcf7180001569831-augustus-gene-0.1-mRNA-1 | SFR17305 |
| maker-LL05_jcf7180001455481-augustus-gene-0.0-mRNA-1 | SFR12345 |
| maker-ML3_jcf7180002033491-augustus-gene-0.0-mRNA-1 | SFR12272 |
| augustus-C10_jcf7180001737345-processed-gene-0.0-mRNA-1 | SFR03563 |
| maker-B1_jcf7180001477361-augustus-gene-0.1-mRNA-1 | SFR14722 |
| maker-C6_jcf7180001949786-augustus-gene-0.1-mRNA-1 | SFR12435 |
| maker-C6_jcf7180001949786-augustus-gene-0.1-mRNA-1 | SFR18094 |
| maker-A10_jcf7180002300378-augustus-gene-0.0-mRNA-1 | SFR12230 |
| maker-KE-8_jcf7180003032669-augustus-gene-0.1-mRNA-1 | SFR17305 |
| maker-D9_jcf7180001862603-augustus-gene-0.1-mRNA-1 | SFR16428 |
| augustus-KE-8_jcf7180002753626-processed-gene-0.2-mRNA-1 | SFR03355 |
| maker-D3_jcf7180002087002-augustus-gene-0.0-mRNA-1 | SFR20956 |
| maker-C9_jcf7180002124936-augustus-gene-0.1-mRNA-1 | SFR21493 |
| augustus-KE-7_jcf7180002570134-processed-gene-0.0-mRNA-1 | SFR14457 |
| augustus-A8_jcf7180001992325-processed-gene-0.0-mRNA-1 | SFR09654 |
| augustus-B5_jcf7180001723852-processed-gene-0.0-mRNA-1 | SFR06004 |
| augustus-ML01_1_jcf7180001480386-processed-gene-0.3-mRNA-1 | SFR21493 |
| maker-D6_jcf7180002007278-augustus-gene-0.0-mRNA-1 | SFR17504 |
| maker-C9_jcf7180002095462-augustus-gene-0.0-mRNA-1 | SFR14626 |
| augustus-D4_jcf7180002094152-processed-gene-0.0-mRNA-1 | SFR03905 |
| maker-B4_jcf7180001659113-augustus-gene-0.1-mRNA-1 | SFR14646 |
| maker-LL06_jcf7180001223908-augustus-gene-0.1-mRNA-1 | SFR06620 |
| maker-A9_jcf7180001982603-augustus-gene-0.1-mRNA-1 | SFR00700 |
| maker-A4_jcf7180002064127-augustus-gene-0.0-mRNA-1 | SFR12720 |
| maker-A4_jcf7180002064127-augustus-gene-0.0-mRNA-1 | SFR13586 |
| maker-B4_jcf7180001630455-augustus-gene-0.0-mRNA-1 | SFR09160 |
| maker-B2_jcf7180001472750-augustus-gene-0.0-mRNA-1 | SFR11202 |
| maker-ML01_3_jcf7180001353275-augustus-gene-0.1-mRNA-1 | SFR19977 |
| maker-KE-6_jcf7180005394862-augustus-gene-0.0-mRNA-1 | SFR02568 |
| maker-B10_jcf7180001488762-augustus-gene-0.1-mRNA-1 | SFR18723 |
| augustus-A10_jcf7180002274408-processed-gene-0.0-mRNA-1 | SFR05219 |
| augustus-D10_jcf7180002220159-processed-gene-0.0-mRNA-1 | SFR01719 |
| augustus-A5_jcf7180002032047-processed-gene-0.0-mRNA-1 | SFR08038 |
| augustus-C7_jcf7180002090019-processed-gene-0.3-mRNA-1 | SFR15203 |
| maker-D9_jcf7180001921240-augustus-gene-0.1-mRNA-1 | SFR18641 |
| maker-KE-7_jcf7180002573782-augustus-gene-0.1-mRNA-1 | SFR03355 |
| maker-D9_jcf7180001880546-augustus-gene-0.0-mRNA-1 | SFR12838 |
| maker-D6_jcf7180002075702-augustus-gene-0.1-mRNA-1 | SFR19617 |
| augustus-B1_jcf7180001508139-processed-gene-0.0-mRNA-1 | SFR15641 |
| augustus-B1_jcf7180001508139-processed-gene-0.0-mRNA-1 | SFR15641 |
| maker-GX01_jcf7180001594755-augustus-gene-0.0-mRNA-1 | SFR03221 |
| maker-B10_jcf7180001496663-augustus-gene-0.0-mRNA-1 | SFR06004 |
| augustus-C4_jcf7180001831602-processed-gene-0.0-mRNA-1 | SFR15235 |
| augustus-C4_jcf7180001831602-processed-gene-0.0-mRNA-1 | SFR01097 |
| augustus-FAW1.1_jcf7180001303879-processed-gene-0.0-mRNA-1 | SFR13561 |
| maker-A10_jcf7180002274633-augustus-gene-0.1-mRNA-1 | SFR08531 |
| maker-C2_jcf7180001833205-augustus-gene-0.1-mRNA-1 | SFR16407 |
| maker-BS01_jcf7180001526345-augustus-gene-0.0-mRNA-1 | SFR14646 |
| maker-D10_jcf7180002012524-augustus-gene-0.0-mRNA-1 | SFR00817 |
| maker-D4_jcf7180002248386-augustus-gene-0.1-mRNA-1 | SFR21681 |
| maker-D4_jcf7180002248386-augustus-gene-0.1-mRNA-1 | SFR19533 |
| augustus-KE-4_jcf7180006969208-processed-gene-0.0-mRNA-1 | SFR17417 |
| augustus-KE-4_jcf7180006969208-processed-gene-0.0-mRNA-1 | SFR14146 |
| maker-A5_jcf7180002141742-augustus-gene-0.0-mRNA-1 | SFR12008 |
| maker-A1_jcf7180001881230-augustus-gene-0.0-mRNA-1 | SFR10791 |
| augustus-A9_jcf7180001785332-processed-gene-0.0-mRNA-1 | SFR09805 |
| augustus-C2_jcf7180001792421-processed-gene-0.0-mRNA-1 | SFR20764 |
| maker-GX02_jcf7180001588054-augustus-gene-0.0-mRNA-1 | SFR18792 |
| augustus-A3_jcf7180002078744-processed-gene-0.0-mRNA-1 | SFR10741 |
| maker-D1_jcf7180002199899-augustus-gene-0.0-mRNA-1 | SFR15277 |
| augustus-GX01_jcf7180001466016-processed-gene-0.0-mRNA-1 | SFR16427 |
| maker-BS01_jcf7180001662170-augustus-gene-0.0-mRNA-1 | SFR02478 |
| maker-BS01_jcf7180001662170-augustus-gene-0.0-mRNA-1 | SFR02692 |
| augustus-A5_jcf7180002106377-processed-gene-0.2-mRNA-1 | SFR01329 |
| maker-KE-4_jcf7180006628437-augustus-gene-0.1-mRNA-1 | SFR12365 |
| maker-FAW2.1_jcf7180001080572-augustus-gene-0.0-mRNA-1 | SFR19984 |
| maker-C8_jcf7180001740711-augustus-gene-0.1-mRNA-1 | SFR06396 |
| maker-D10_jcf7180002184353-augustus-gene-0.0-mRNA-1 | SFR05222 |
| maker-KE-10_jcf7180003427714-augustus-gene-0.0-mRNA-1 | SFR19351 |
| maker-C2_jcf7180001916542-augustus-gene-0.0-mRNA-1 | SFR13561 |
| augustus-A5_jcf7180001938379-processed-gene-0.0-mRNA-1 | SFR10861 |
| augustus-D3_jcf7180002108309-processed-gene-0.3-mRNA-1 | SFR15139 |
| maker-A5_jcf7180002066171-augustus-gene-0.0-mRNA-1 | SFR22117 |
| maker-A8_jcf7180001977381-augustus-gene-0.1-mRNA-1 | SFR00395 |
| augustus-A3_jcf7180002118495-processed-gene-0.0-mRNA-1 | SFR03355 |
| augustus-D4_jcf7180002132543-processed-gene-0.2-mRNA-1 | SFR21792 |
| maker-B2_jcf7180001624393-augustus-gene-0.1-mRNA-1 | SFR10847 |
| augustus-KE-4_jcf7180007017547-processed-gene-0.0-mRNA-1 | SFR09529 |
| maker-A9_jcf7180001955779-augustus-gene-0.1-mRNA-1 | SFR22016 |
| maker-A1_jcf7180001978507-augustus-gene-0.0-mRNA-1 | SFR20438 |
| augustus-B2_jcf7180001594103-processed-gene-0.0-mRNA-1 | SFR07744 |
| augustus-B2_jcf7180001594103-processed-gene-0.0-mRNA-1 | SFR09181 |
| augustus-LJ01_jcf7180001335764-processed-gene-0.0-mRNA-1 | SFR16842 |
| maker-A9_jcf7180001983627-augustus-gene-0.0-mRNA-1 | SFR04694 |
| maker-LL09_jcf7180001553089-augustus-gene-0.0-mRNA-1 | SFR13997 |
| maker-B4_jcf7180001535740-augustus-gene-0.1-mRNA-1 | SFR17566 |
| maker-KE-7_jcf7180002445377-augustus-gene-0.0-mRNA-1 | SFR22190 |
| maker-KE-7_jcf7180002445377-augustus-gene-0.0-mRNA-1 | SFR13043 |
| maker-A6_jcf7180001733821-augustus-gene-0.0-mRNA-1 | SFR09162 |
| maker-A9_jcf7180001959184-augustus-gene-0.0-mRNA-1 | SFR18001 |
| maker-B5_jcf7180001753458-augustus-gene-0.1-mRNA-1 | SFR11202 |
| augustus-B6_jcf7180001638361-processed-gene-0.0-mRNA-1 | SFR22145 |
| augustus-A8_jcf7180002016674-processed-gene-0.0-mRNA-1 | SFR02414 |
| maker-D8_jcf7180002034765-augustus-gene-0.0-mRNA-1 | SFR02538 |
| maker-A10_jcf7180002280165-augustus-gene-0.0-mRNA-1 | SFR13010 |
| maker-A10_jcf7180002280165-augustus-gene-0.0-mRNA-1 | SFR03391 |
| maker-KE-4_jcf7180006629539-augustus-gene-0.1-mRNA-1 | SFR04713 |
| augustus-A9_jcf7180001775478-processed-gene-0.0-mRNA-1 | SFR17504 |
| augustus-D8_jcf7180002083688-processed-gene-0.0-mRNA-1 | SFR07393 |
| maker-D10_jcf7180002170813-augustus-gene-0.0-mRNA-1 | SFR15623 |
| maker-D10_jcf7180002170813-augustus-gene-0.0-mRNA-1 | SFR15623 |
| maker-D3_jcf7180001980840-augustus-gene-0.0-mRNA-1 | SFR15467 |
| maker-A2_jcf7180001747588-augustus-gene-0.1-mRNA-1 | SFR10969 |
| maker-A9_jcf7180001833787-augustus-gene-0.0-mRNA-1 | SFR10111 |
| maker-C10_jcf7180001674561-augustus-gene-0.1-mRNA-1 | SFR00492 |
| maker-D4_jcf7180002113848-augustus-gene-0.1-mRNA-1 | SFR08904 |
| maker-A7_jcf7180002044650-augustus-gene-0.1-mRNA-1 | SFR05225 |
| maker-A7_jcf7180002044650-augustus-gene-0.1-mRNA-1 | SFR12493 |
| augustus-B5_jcf7180001729679-processed-gene-0.0-mRNA-1 | SFR02763 |
| augustus-A6_jcf7180001635886-processed-gene-0.0-mRNA-1 | SFR05017 |
| maker-A2_jcf7180001944041-augustus-gene-0.0-mRNA-1 | SFR07007 |
| maker-B8_jcf7180001760892-augustus-gene-0.1-mRNA-1 | SFR04642 |
| maker-D7_jcf7180001848818-augustus-gene-0.1-mRNA-1 | SFR15840 |
| maker-C3_jcf7180001804158-augustus-gene-0.0-mRNA-1 | SFR22145 |
| augustus-A7_jcf7180001946657-processed-gene-0.2-mRNA-1 | SFR09207 |
| maker-FJ01_3_jcf7180001455264-augustus-gene-0.1-mRNA-1 | SFR17593 |
| maker-B8_jcf7180001752946-augustus-gene-0.1-mRNA-1 | SFR20845 |
| maker-D6_jcf7180002183076-augustus-gene-0.1-mRNA-1 | SFR20928 |
| maker-GX02_jcf7180001518500-augustus-gene-0.0-mRNA-1 | SFR09208 |
| maker-A10_jcf7180002184866-augustus-gene-0.1-mRNA-1 | SFR06396 |
| maker-B8_jcf7180001738549-augustus-gene-0.1-mRNA-1 | SFR08989 |
| maker-B9_jcf7180001504001-augustus-gene-0.0-mRNA-1 | SFR12362 |
| augustus-B9_jcf7180001579515-processed-gene-0.0-mRNA-1 | SFR11890 |
| maker-A3_jcf7180002118286-augustus-gene-0.0-mRNA-1 | SFR16467 |
| maker-A3_jcf7180002118286-augustus-gene-0.0-mRNA-1 | SFR05222 |
| maker-B1_jcf7180001486004-augustus-gene-0.0-mRNA-1 | SFR00462 |
| maker-D3_jcf7180001944033-augustus-gene-0.0-mRNA-1 | SFR14074 |
| maker-D1_jcf7180001975247-augustus-gene-0.0-mRNA-1 | SFR02250 |
| augustus-A8_jcf7180002135338-processed-gene-0.0-mRNA-1 | SFR16628 |
| augustus-GX03_jcf7180001456228-processed-gene-0.0-mRNA-1 | SFR05985 |
| maker-D6_jcf7180002142564-augustus-gene-0.1-mRNA-1 | SFR04318 |
| maker-A6_jcf7180001753998-augustus-gene-0.0-mRNA-1 | SFR04295 |
| maker-A5_jcf7180002002485-augustus-gene-0.0-mRNA-1 | SFR06877 |
| maker-A8_jcf7180002093787-augustus-gene-0.1-mRNA-1 | SFR07769 |
| maker-D4_jcf7180002282022-augustus-gene-0.0-mRNA-1 | SFR11865 |
| maker-KE-7_jcf7180002441511-augustus-gene-0.1-mRNA-1 | SFR09654 |
| maker-FAW5.2_jcf7180001421973-augustus-gene-0.0-mRNA-1 | SFR10057 |
| augustus-ML3_jcf7180001878211-processed-gene-0.0-mRNA-1 | SFR17448 |
| maker-ML1_jcf7180001999101-augustus-gene-0.1-mRNA-1 | SFR00635 |
| augustus-B8_jcf7180001766791-processed-gene-0.0-mRNA-1 | SFR07565 |
| maker-A2_jcf7180001819718-augustus-gene-0.1-mRNA-1 | SFR00637 |
| maker-B5_jcf7180001578426-augustus-gene-0.1-mRNA-1 | SFR20654 |
| maker-B5_jcf7180001578426-augustus-gene-0.1-mRNA-1 | SFR18369 |
| augustus-A8_jcf7180002104451-processed-gene-0.0-mRNA-1 | SFR15683 |
| maker-A4_jcf7180001892334-augustus-gene-0.0-mRNA-1 | SFR06488 |
| maker-A4_jcf7180001892334-augustus-gene-0.0-mRNA-1 | SFR20111 |
| maker-C2_jcf7180001794182-augustus-gene-0.0-mRNA-1 | SFR04438 |
| maker-C2_jcf7180001794182-augustus-gene-0.0-mRNA-1 | SFR08482 |
| maker-FAW1.1_jcf7180001405437-augustus-gene-0.0-mRNA-1 | SFR07639 |
| maker-KE-5_jcf7180003431768-augustus-gene-0.1-mRNA-1 | SFR08304 |
| maker-KE-5_jcf7180003431768-augustus-gene-0.1-mRNA-1 | SFR08242 |
| maker-KE-8_jcf7180002797255-augustus-gene-0.0-mRNA-1 | SFR06877 |
| maker-A5_jcf7180001948334-augustus-gene-0.0-mRNA-1 | SFR14531 |
| maker-FAW1.1_jcf7180001376194-augustus-gene-0.0-mRNA-1 | SFR06015 |
| maker-ML01_1_jcf7180001489152-augustus-gene-0.1-mRNA-1 | SFR15293 |
| maker-ML3_jcf7180001875952-augustus-gene-0.0-mRNA-1 | SFR10289 |
| maker-C9_jcf7180002007583-augustus-gene-0.0-mRNA-1 | SFR00686 |
| maker-FJ01_3_jcf7180001402485-augustus-gene-0.1-mRNA-1 | SFR02920 |
| maker-ML01_3_jcf7180001350817-augustus-gene-0.0-mRNA-1 | SFR03971 |
| augustus-D7_jcf7180001920128-processed-gene-0.0-mRNA-1 | SFR10217 |
| maker-A8_jcf7180002021728-augustus-gene-0.1-mRNA-1 | SFR19617 |
| maker-KE-10_jcf7180003499773-augustus-gene-0.0-mRNA-1 | SFR06877 |
| maker-A9_jcf7180001957808-augustus-gene-0.0-mRNA-1 | SFR03053 |
| augustus-A9_jcf7180001816131-processed-gene-0.0-mRNA-1 | SFR13736 |
| maker-C2_jcf7180001742036-augustus-gene-0.1-mRNA-1 | SFR21493 |
| augustus-KE-7_jcf7180002601202-processed-gene-0.0-mRNA-1 | SFR18824 |
| augustus-LL04_jcf7180001366900-processed-gene-0.0-mRNA-1 | SFR03257 |
| maker-A8_jcf7180002012319-augustus-gene-0.0-mRNA-1 | SFR06877 |
| maker-FAW2.2_jcf7180001094672-augustus-gene-0.0-mRNA-1 | SFR14868 |
| augustus-KE-5_jcf7180003178106-processed-gene-0.0-mRNA-1 | SFR11472 |
| augustus-A5_jcf7180002055078-processed-gene-0.0-mRNA-1 | SFR10217 |
| maker-D8_jcf7180001882242-augustus-gene-0.0-mRNA-1 | SFR19013 |
| maker-ML01_3_jcf7180001421837-augustus-gene-0.0-mRNA-1 | SFR12265 |
| maker-C10_jcf7180001602848-augustus-gene-0.1-mRNA-1 | SFR20396 |
| augustus-C2_jcf7180001903025-processed-gene-0.0-mRNA-1 | SFR08260 |
| maker-A8_jcf7180002086962-augustus-gene-0.0-mRNA-1 | SFR08440 |
| maker-A4_jcf7180002069866-augustus-gene-0.1-mRNA-1 | SFR16841 |
| maker-KE-8_jcf7180002793368-augustus-gene-0.0-mRNA-1 | SFR19613 |
| maker-B8_jcf7180001691051-augustus-gene-0.0-mRNA-1 | SFR13564 |
| maker-B5_jcf7180001592602-augustus-gene-0.1-mRNA-1 | SFR18005 |
| augustus-A2_jcf7180001925165-processed-gene-0.0-mRNA-1 | SFR14176 |
| maker-D2_jcf7180002015375-augustus-gene-0.1-mRNA-1 | SFR09735 |
| augustus-A3_jcf7180002001340-processed-gene-0.0-mRNA-1 | SFR13244 |
| augustus-A4_jcf7180001940224-processed-gene-0.0-mRNA-1 | SFR20921 |
| maker-C4_jcf7180001771392-augustus-gene-0.0-mRNA-1 | SFR13005 |
| maker-KE-3_jcf7180003594612-augustus-gene-0.1-mRNA-1 | SFR02351 |
| augustus-C9_jcf7180002137924-processed-gene-0.0-mRNA-1 | SFR18996 |
| maker-A7_jcf7180001873850-augustus-gene-0.0-mRNA-1 | SFR09761 |
| maker-C8_jcf7180001664257-augustus-gene-0.0-mRNA-1 | SFR00462 |
| maker-A10_jcf7180002273277-augustus-gene-0.1-mRNA-1 | SFR12362 |
| maker-A8_jcf7180002046062-augustus-gene-0.0-mRNA-1 | SFR08904 |
| augustus-FAW1.1_jcf7180001428370-processed-gene-0.0-mRNA-1 | SFR13807 |
| maker-GX01_jcf7180001514943-augustus-gene-0.0-mRNA-1 | SFR16401 |
| maker-GX01_jcf7180001514943-augustus-gene-0.0-mRNA-1 | SFR13404 |
| maker-KE-6_jcf7180005410882-augustus-gene-0.1-mRNA-1 | SFR06763 |
| maker-A2_jcf7180001924186-augustus-gene-0.0-mRNA-1 | SFR20822 |
| maker-D6_jcf7180002179541-augustus-gene-0.1-mRNA-1 | SFR15203 |
| augustus-C2_jcf7180001933049-processed-gene-0.2-mRNA-1 | SFR02920 |
| augustus-A4_jcf7180002002826-processed-gene-0.2-mRNA-1 | SFR19351 |
| augustus-A8_jcf7180001984311-processed-gene-0.0-mRNA-1 | SFR19507 |
| augustus-MH01_jcf7180001510685-processed-gene-0.0-mRNA-1 | SFR21782 |
| augustus-MH01_jcf7180001510685-processed-gene-0.0-mRNA-1 | SFR12261 |
| maker-KE-3_jcf7180003493381-augustus-gene-0.1-mRNA-1 | SFR17093 |
| maker-KE-3_jcf7180003493381-augustus-gene-0.1-mRNA-1 | SFR17088 |
| maker-D9_jcf7180001938454-augustus-gene-0.0-mRNA-1 | SFR01251 |
| maker-D9_jcf7180001938454-augustus-gene-0.0-mRNA-1 | SFR01348 |
| augustus-C7_jcf7180002139020-processed-gene-0.0-mRNA-1 | SFR11819 |
| augustus-C1_jcf7180001939503-processed-gene-0.2-mRNA-1 | SFR21414 |
| augustus-C1_jcf7180001939503-processed-gene-0.2-mRNA-1 | SFR06438 |
| augustus-GX01_jcf7180001475085-processed-gene-0.0-mRNA-1 | SFR18566 |
| maker-A4_jcf7180002062825-augustus-gene-0.0-mRNA-1 | SFR21639 |
| maker-A5_jcf7180002001179-augustus-gene-0.1-mRNA-1 | SFR09761 |
| maker-C1_jcf7180001977832-augustus-gene-0.1-mRNA-1 | SFR11576 |
| augustus-A8_jcf7180002145267-processed-gene-0.0-mRNA-1 | SFR01914 |
| augustus-A8_jcf7180002145267-processed-gene-0.0-mRNA-1 | SFR12276 |
| maker-C7_jcf7180002090517-augustus-gene-0.0-mRNA-1 | SFR08010 |
| augustus-A3_jcf7180001992271-processed-gene-0.0-mRNA-1 | SFR05927 |
| augustus-A3_jcf7180001992271-processed-gene-0.0-mRNA-1 | SFR12796 |
| augustus-KE-9_jcf7180003150314-processed-gene-0.0-mRNA-1 | SFR12265 |
| maker-D8_jcf7180001929279-augustus-gene-0.1-mRNA-1 | SFR19013 |
| maker-BS01_jcf7180001676733-augustus-gene-0.0-mRNA-1 | SFR06763 |
| maker-C7_jcf7180002102478-augustus-gene-0.1-mRNA-1 | SFR12362 |
| augustus-GX02_jcf7180001448541-processed-gene-0.3-mRNA-1 | SFR06498 |
| augustus-ML3_jcf7180001830285-processed-gene-0.0-mRNA-1 | SFR14939 |
| augustus-ML3_jcf7180001830285-processed-gene-0.0-mRNA-1 | SFR18064 |
| maker-C4_jcf7180001944017-augustus-gene-0.1-mRNA-1 | SFR05110 |
| maker-C3_jcf7180001968666-augustus-gene-0.0-mRNA-1 | SFR08866 |
| maker-B9_jcf7180001482266-augustus-gene-0.1-mRNA-1 | SFR10765 |
| maker-A7_jcf7180001900766-augustus-gene-0.0-mRNA-1 | SFR06498 |
| augustus-KE-8_jcf7180002777099-processed-gene-0.2-mRNA-1 | SFR07863 |
| maker-C10_jcf7180001680825-augustus-gene-0.1-mRNA-1 | SFR10250 |
| maker-D6_jcf7180002199923-augustus-gene-0.0-mRNA-1 | SFR10183 |
| maker-A1_jcf7180001977411-augustus-gene-0.0-mRNA-1 | SFR06498 |
| augustus-C4_jcf7180001867276-processed-gene-0.0-mRNA-1 | SFR17305 |
| maker-C7_jcf7180002092301-augustus-gene-0.0-mRNA-1 | SFR15126 |
| maker-A10_jcf7180002078218-augustus-gene-0.1-mRNA-1 | SFR11202 |
| augustus-A6_jcf7180001730173-processed-gene-0.0-mRNA-1 | SFR17253 |
| maker-A4_jcf7180001889499-augustus-gene-0.1-mRNA-1 | SFR03355 |
| augustus-C6_jcf7180002027598-processed-gene-0.0-mRNA-1 | SFR12838 |
| maker-A4_jcf7180001933423-augustus-gene-0.0-mRNA-1 | SFR16467 |
| maker-A4_jcf7180001933423-augustus-gene-0.0-mRNA-1 | SFR05222 |
| augustus-A10_jcf7180002147127-processed-gene-0.0-mRNA-1 | SFR13213 |
| maker-D8_jcf7180001934139-augustus-gene-0.0-mRNA-1 | SFR19037 |
| augustus-FJ01_2_jcf7180001257566-processed-gene-0.0-mRNA-1 | SFR09918 |
| maker-KE-6_jcf7180005303175-augustus-gene-0.0-mRNA-1 | SFR11202 |
| augustus-A10_jcf7180002309463-processed-gene-0.0-mRNA-1 | SFR03096 |
| maker-A2_jcf7180001800177-augustus-gene-0.1-mRNA-1 | SFR19613 |
| augustus-FAW5.2_jcf7180001426734-processed-gene-0.0-mRNA-1 | SFR21016 |
| maker-KE-7_jcf7180002688520-augustus-gene-0.0-mRNA-1 | SFR12804 |
| maker-KE-7_jcf7180002688520-augustus-gene-0.0-mRNA-1 | SFR12260 |
| maker-ML3_jcf7180001830672-augustus-gene-0.1-mRNA-1 | SFR15293 |
| augustus-ML1_jcf7180002097420-processed-gene-0.0-mRNA-1 | SFR22117 |
| augustus-KE-3_jcf7180003446961-processed-gene-0.0-mRNA-1 | SFR18177 |
| maker-A8_jcf7180002158747-augustus-gene-0.0-mRNA-1 | SFR15069 |
| augustus-C9_jcf7180002124042-processed-gene-0.0-mRNA-1 | SFR06396 |
| maker-D7_jcf7180001852775-augustus-gene-0.0-mRNA-1 | SFR17818 |
| augustus-D4_jcf7180002096159-processed-gene-0.0-mRNA-1 | SFR20654 |
| augustus-D4_jcf7180002096159-processed-gene-0.0-mRNA-1 | SFR18369 |
| maker-B9_jcf7180001504466-augustus-gene-0.0-mRNA-1 | SFR12608 |
| augustus-LL01_2_jcf7180001197440-processed-gene-0.0-mRNA-1 | SFR19488 |
| maker-A1_jcf7180001973009-augustus-gene-0.1-mRNA-1 | SFR04589 |
| augustus-GX03_jcf7180001387294-processed-gene-0.0-mRNA-1 | SFR04696 |
| augustus-B5_jcf7180001607035-processed-gene-0.0-mRNA-1 | SFR17435 |
| maker-KE-10_jcf7180003273330-augustus-gene-0.1-mRNA-1 | SFR16163 |
| maker-B8_jcf7180001684425-augustus-gene-0.0-mRNA-1 | SFR20928 |
| augustus-ML1_jcf7180001929992-processed-gene-0.2-mRNA-1 | SFR12442 |
| maker-B6_jcf7180001567133-augustus-gene-0.0-mRNA-1 | SFR09629 |
| maker-A10_jcf7180002080427-augustus-gene-0.0-mRNA-1 | SFR10057 |
| maker-A7_jcf7180001856515-augustus-gene-0.1-mRNA-1 | SFR10752 |
| augustus-B1_jcf7180001476514-processed-gene-0.0-mRNA-1 | SFR09012 |
| augustus-C9_jcf7180002123791-processed-gene-0.0-mRNA-1 | SFR12442 |
| maker-A7_jcf7180001869642-augustus-gene-0.1-mRNA-1 | SFR18773 |
| maker-A10_jcf7180002093305-augustus-gene-0.0-mRNA-1 | SFR12004 |
| maker-A9_jcf7180001832018-augustus-gene-0.0-mRNA-1 | SFR10234 |
| maker-BS02_jcf7180001519694-augustus-gene-0.0-mRNA-1 | SFR20994 |
| maker-A5_jcf7180002130821-augustus-gene-0.0-mRNA-1 | SFR17872 |
| augustus-FAW2.1_jcf7180001079485-processed-gene-0.0-mRNA-1 | SFR11904 |
| augustus-FAW2.1_jcf7180001079485-processed-gene-0.0-mRNA-1 | SFR11905 |
| augustus-A9_jcf7180001846698-processed-gene-0.0-mRNA-1 | SFR08614 |
| augustus-A5_jcf7180002021531-processed-gene-0.2-mRNA-1 | SFR03526 |
| augustus-A5_jcf7180002021531-processed-gene-0.2-mRNA-1 | SFR08872 |
| maker-KE-5_jcf7180003421211-augustus-gene-0.0-mRNA-1 | SFR17920 |
| maker-KE-5_jcf7180003431898-augustus-gene-0.1-mRNA-1 | SFR03221 |
| augustus-D3_jcf7180002072135-processed-gene-0.0-mRNA-1 | SFR10662 |
| maker-A3_jcf7180002118223-augustus-gene-0.1-mRNA-1 | SFR20928 |
| augustus-D7_jcf7180001894461-processed-gene-0.0-mRNA-1 | SFR16951 |
| augustus-D7_jcf7180001894461-processed-gene-0.0-mRNA-1 | SFR16687 |
| maker-A4_jcf7180001896755-augustus-gene-0.1-mRNA-1 | SFR02855 |
| maker-A5_jcf7180002127121-augustus-gene-0.1-mRNA-1 | SFR02509 |
| augustus-B5_jcf7180001688455-processed-gene-0.0-mRNA-1 | SFR03096 |
| maker-KE-3_jcf7180003435451-augustus-gene-0.0-mRNA-1 | SFR16467 |
| maker-KE-3_jcf7180003435451-augustus-gene-0.0-mRNA-1 | SFR05222 |
| augustus-A10_jcf7180002077203-processed-gene-0.2-mRNA-1 | SFR14500 |
| augustus-C6_jcf7180002027566-processed-gene-0.0-mRNA-1 | SFR13581 |
| maker-B8_jcf7180001600243-augustus-gene-0.0-mRNA-1 | SFR12362 |
| augustus-FAW2.1_jcf7180001101150-processed-gene-0.0-mRNA-1 | SFR04375 |
| augustus-FAW2.1_jcf7180001101150-processed-gene-0.0-mRNA-1 | SFR08271 |
| maker-A9_jcf7180001961628-augustus-gene-0.1-mRNA-1 | SFR02626 |
| maker-A3_jcf7180002001541-augustus-gene-0.0-mRNA-1 | SFR17403 |
| maker-KE-10_jcf7180003319611-augustus-gene-0.0-mRNA-1 | SFR09185 |
| maker-A7_jcf7180002073271-augustus-gene-0.1-mRNA-1 | SFR09185 |
| maker-A4_jcf7180001943158-augustus-gene-0.0-mRNA-1 | SFR12668 |
| maker-LL01_3_jcf7180001447339-augustus-gene-0.0-mRNA-1 | SFR16067 |
| maker-LL01_3_jcf7180001447339-augustus-gene-0.0-mRNA-1 | SFR18287 |
| maker-A7_jcf7180002043838-augustus-gene-0.1-mRNA-1 | SFR21851 |
| maker-C8_jcf7180001795792-augustus-gene-0.0-mRNA-1 | SFR09381 |
| maker-FAW5.2_jcf7180001417763-augustus-gene-0.1-mRNA-1 | SFR04676 |
| augustus-FAW5.2_jcf7180001391470-processed-gene-0.0-mRNA-1 | SFR18097 |
| maker-A8_jcf7180002126611-augustus-gene-0.0-mRNA-1 | SFR16784 |
| augustus-D8_jcf7180001931432-processed-gene-0.0-mRNA-1 | SFR19362 |
| augustus-D8_jcf7180001931432-processed-gene-0.0-mRNA-1 | SFR08845 |
| maker-ML3_jcf7180001921366-augustus-gene-0.0-mRNA-1 | SFR18586 |
| maker-B9_jcf7180001556731-augustus-gene-0.1-mRNA-1 | SFR07338 |
| augustus-A7_jcf7180001944822-processed-gene-0.0-mRNA-1 | SFR15791 |
| augustus-A1_jcf7180001914006-processed-gene-0.0-mRNA-1 | SFR06488 |
| augustus-A1_jcf7180001914006-processed-gene-0.0-mRNA-1 | SFR20111 |
| augustus-LL01_3_jcf7180001402411-processed-gene-0.0-mRNA-1 | SFR19700 |
| maker-JC01_jcf7180001381925-augustus-gene-0.0-mRNA-1 | SFR09923 |
| augustus-KE-4_jcf7180007063808-processed-gene-0.0-mRNA-1 | SFR18543 |
| maker-FAW1.1_jcf7180001328797-augustus-gene-0.0-mRNA-1 | SFR22145 |
| maker-A10_jcf7180002078721-augustus-gene-0.0-mRNA-1 | SFR08944 |
| augustus-A7_jcf7180002073128-processed-gene-0.0-mRNA-1 | SFR12365 |
| maker-KE-9_jcf7180003149538-augustus-gene-0.1-mRNA-1 | SFR08226 |
| maker-A5_jcf7180001949204-augustus-gene-0.1-mRNA-1 | SFR02818 |
| maker-C1_jcf7180001894447-augustus-gene-0.1-mRNA-1 | SFR12265 |
| maker-KE-10_jcf7180003382932-augustus-gene-0.1-mRNA-1 | SFR01851 |
| maker-D4_jcf7180002239916-augustus-gene-0.0-mRNA-1 | SFR04694 |
| augustus-A10_jcf7180002211285-processed-gene-0.2-mRNA-1 | SFR17404 |
| maker-D7_jcf7180001844819-augustus-gene-0.0-mRNA-1 | SFR18512 |
| augustus-D8_jcf7180002052903-processed-gene-0.0-mRNA-1 | SFR07686 |
| augustus-D8_jcf7180002052903-processed-gene-0.0-mRNA-1 | SFR15233 |
| augustus-B4_jcf7180001628499-processed-gene-0.1-mRNA-1 | SFR20654 |
| augustus-B4_jcf7180001628499-processed-gene-0.1-mRNA-1 | SFR18369 |
| maker-ML1_jcf7180002061956-augustus-gene-0.1-mRNA-1 | SFR04916 |
| maker-D7_jcf7180001843926-augustus-gene-0.0-mRNA-1 | SFR03221 |
| augustus-D6_jcf7180002115001-processed-gene-0.0-mRNA-1 | SFR15235 |
| augustus-D6_jcf7180002115001-processed-gene-0.0-mRNA-1 | SFR01097 |
| augustus-B5_jcf7180001720771-processed-gene-0.0-mRNA-1 | SFR22189 |
| augustus-A5_jcf7180001957141-processed-gene-0.0-mRNA-1 | SFR14104 |
| augustus-D4_jcf7180002281989-processed-gene-0.2-mRNA-1 | SFR20867 |
| augustus-A9_jcf7180001813348-processed-gene-0.1-mRNA-1 | SFR17093 |
| augustus-A9_jcf7180001813348-processed-gene-0.1-mRNA-1 | SFR17088 |
| maker-ML1_jcf7180002068862-augustus-gene-0.1-mRNA-1 | SFR08812 |
| maker-A9_jcf7180001791857-augustus-gene-0.1-mRNA-1 | SFR14299 |
| augustus-D2_jcf7180002018989-processed-gene-0.0-mRNA-1 | SFR19222 |
| augustus-C6_jcf7180001906154-processed-gene-0.0-mRNA-1 | SFR08434 |
| maker-D8_jcf7180001910524-augustus-gene-0.0-mRNA-1 | SFR04524 |
| maker-A10_jcf7180002204553-augustus-gene-0.2-mRNA-1 | SFR03479 |
| maker-A5_jcf7180002133897-augustus-gene-0.0-mRNA-1 | SFR22145 |
| augustus-FJ01_3_jcf7180001527756-processed-gene-0.0-mRNA-1 | SFR16637 |
| augustus-ML01_1_jcf7180001443129-processed-gene-0.0-mRNA-1 | SFR06877 |
| maker-D5_jcf7180001827048-augustus-gene-0.1-mRNA-1 | SFR18211 |
| augustus-A5_jcf7180002146200-processed-gene-0.0-mRNA-1 | SFR18388 |
| maker-GX04_jcf7180001142653-augustus-gene-0.1-mRNA-1 | SFR18482 |
| maker-D9_jcf7180001762666-augustus-gene-0.1-mRNA-1 | SFR07863 |
| augustus-FAW1.1_jcf7180001335088-processed-gene-0.0-mRNA-1 | SFR01286 |
| maker-BS02_jcf7180001420850-augustus-gene-0.1-mRNA-1 | SFR00462 |
| augustus-A5_jcf7180002162448-processed-gene-0.0-mRNA-1 | SFR04416 |
| maker-A8_jcf7180002069803-augustus-gene-0.0-mRNA-1 | SFR04551 |
| maker-C6_jcf7180001920174-augustus-gene-0.0-mRNA-1 | SFR12367 |
| maker-C6_jcf7180001920174-augustus-gene-0.0-mRNA-1 | SFR08544 |
| maker-A7_jcf7180001919257-augustus-gene-0.1-mRNA-1 | SFR14103 |
| maker-A3_jcf7180002081456-augustus-gene-0.0-mRNA-1 | SFR01914 |
| maker-A3_jcf7180002081456-augustus-gene-0.0-mRNA-1 | SFR12276 |
| maker-D8_jcf7180002035010-augustus-gene-0.0-mRNA-1 | SFR09888 |
| maker-A10_jcf7180002291340-augustus-gene-0.1-mRNA-1 | SFR12983 |
| maker-GX02_jcf7180001569773-augustus-gene-0.1-mRNA-1 | SFR03096 |
| maker-D4_jcf7180002280928-augustus-gene-0.1-mRNA-1 | SFR04438 |
| maker-D4_jcf7180002280928-augustus-gene-0.1-mRNA-1 | SFR08482 |
| maker-B8_jcf7180001697558-augustus-gene-0.0-mRNA-1 | SFR19848 |
| augustus-B9_jcf7180001437382-processed-gene-0.1-mRNA-1 | SFR15641 |
| augustus-B9_jcf7180001437382-processed-gene-0.1-mRNA-1 | SFR15641 |
| maker-LJ01_jcf7180001413395-augustus-gene-0.1-mRNA-1 | SFR18388 |
| augustus-A7_jcf7180001920194-processed-gene-0.0-mRNA-1 | SFR02763 |
| maker-A9_jcf7180001962541-augustus-gene-0.1-mRNA-1 | SFR17872 |
| maker-D2_jcf7180001986730-augustus-gene-0.0-mRNA-1 | SFR15170 |
| maker-C2_jcf7180001754574-augustus-gene-0.1-mRNA-1 | SFR20867 |
| maker-A4_jcf7180001888989-augustus-gene-0.1-mRNA-1 | SFR03945 |
| augustus-C7_jcf7180001919519-processed-gene-0.3-mRNA-1 | SFR21851 |
| maker-B9_jcf7180001585544-augustus-gene-0.1-mRNA-1 | SFR05515 |
| maker-A10_jcf7180002170532-augustus-gene-0.1-mRNA-1 | SFR00846 |
| maker-A10_jcf7180002170532-augustus-gene-0.1-mRNA-1 | SFR03047 |
| maker-D1_jcf7180001962950-augustus-gene-0.1-mRNA-1 | SFR19848 |
| augustus-A10_jcf7180002088012-processed-gene-0.0-mRNA-1 | SFR19351 |
| maker-B4_jcf7180001516342-augustus-gene-0.0-mRNA-1 | SFR12362 |
| augustus-C10_jcf7180001698963-processed-gene-0.0-mRNA-1 | SFR12118 |
| augustus-C9_jcf7180002176120-processed-gene-0.0-mRNA-1 | SFR17566 |
| maker-C3_jcf7180001795739-augustus-gene-0.0-mRNA-1 | SFR04169 |
| augustus-A8_jcf7180002021630-processed-gene-0.0-mRNA-1 | SFR21483 |
| augustus-B10_jcf7180001550608-processed-gene-0.0-mRNA-1 | SFR16196 |
| maker-A5_jcf7180002136407-augustus-gene-0.1-mRNA-1 | SFR18024 |
| maker-ML3_jcf7180001837065-augustus-gene-0.0-mRNA-1 | SFR04551 |
| augustus-C10_jcf7180001739190-processed-gene-0.0-mRNA-1 | SFR10217 |
| maker-C6_jcf7180002025391-augustus-gene-0.1-mRNA-1 | SFR02438 |
| maker-ML3_jcf7180001991615-augustus-gene-0.1-mRNA-1 | SFR08645 |
| maker-LL06_jcf7180001257781-augustus-gene-0.1-mRNA-1 | SFR15449 |
| maker-C6_jcf7180001932516-augustus-gene-0.0-mRNA-1 | SFR21493 |
| maker-D1_jcf7180001962117-augustus-gene-0.0-mRNA-1 | SFR06877 |
| maker-D2_jcf7180001863227-augustus-gene-0.1-mRNA-1 | SFR00470 |
| maker-LL06_jcf7180001233601-augustus-gene-0.1-mRNA-1 | SFR05531 |
| augustus-A8_jcf7180002000217-processed-gene-0.2-mRNA-1 | SFR14500 |
| maker-B1_jcf7180001633045-augustus-gene-0.0-mRNA-1 | SFR06498 |
| maker-C8_jcf7180001668533-augustus-gene-0.1-mRNA-1 | SFR04943 |
| augustus-D1_jcf7180001988783-processed-gene-0.0-mRNA-1 | SFR19179 |
| augustus-D1_jcf7180001988783-processed-gene-0.0-mRNA-1 | SFR18488 |
| maker-A4_jcf7180001913240-augustus-gene-0.1-mRNA-1 | SFR18388 |
| augustus-B4_jcf7180001592613-processed-gene-0.0-mRNA-1 | SFR07696 |
| maker-D9_jcf7180001751981-augustus-gene-0.1-mRNA-1 | SFR14963 |
| maker-BS01_jcf7180001676686-augustus-gene-0.0-mRNA-1 | SFR09185 |
| augustus-A7_jcf7180001924399-processed-gene-0.0-mRNA-1 | SFR18288 |
| augustus-A7_jcf7180001924399-processed-gene-0.0-mRNA-1 | SFR08205 |
| maker-D4_jcf7180002302080-augustus-gene-0.1-mRNA-1 | SFR19126 |
| maker-A1_jcf7180001924121-augustus-gene-0.1-mRNA-1 | SFR00470 |
| maker-A2_jcf7180001890367-augustus-gene-0.1-mRNA-1 | SFR16485 |
| maker-A2_jcf7180001890367-augustus-gene-0.1-mRNA-1 | SFR16488 |
| maker-FAW1.1_jcf7180001318815-augustus-gene-0.1-mRNA-1 | SFR10212 |
| maker-D3_jcf7180001995722-augustus-gene-0.1-mRNA-1 | SFR17193 |
| maker-D8_jcf7180001953242-augustus-gene-0.0-mRNA-1 | SFR09983 |
| maker-B4_jcf7180001515892-augustus-gene-0.0-mRNA-1 | SFR18723 |
| maker-D1_jcf7180002162291-augustus-gene-0.0-mRNA-1 | SFR09366 |
| augustus-C8_jcf7180001738682-processed-gene-0.0-mRNA-1 | SFR04169 |
| augustus-A7_jcf7180001878762-processed-gene-0.0-mRNA-1 | SFR08835 |
| augustus-D6_jcf7180002182938-processed-gene-0.0-mRNA-1 | SFR07763 |
| augustus-D6_jcf7180002190980-processed-gene-0.0-mRNA-1 | SFR22189 |
| maker-B5_jcf7180001727657-augustus-gene-0.0-mRNA-1 | SFR03640 |
| maker-A2_jcf7180001768388-augustus-gene-0.0-mRNA-1 | SFR21200 |
| maker-D6_jcf7180002180587-augustus-gene-0.0-mRNA-1 | SFR08251 |
| maker-D6_jcf7180002180587-augustus-gene-0.0-mRNA-1 | SFR06801 |
| maker-C9_jcf7180001973191-augustus-gene-0.0-mRNA-1 | SFR13001 |
| augustus-A1_jcf7180001874694-processed-gene-0.0-mRNA-1 | SFR12626 |
| maker-D2_jcf7180002035622-augustus-gene-0.1-mRNA-1 | SFR15293 |
| maker-A10_jcf7180002288554-augustus-gene-0.0-mRNA-1 | SFR06620 |
| maker-D8_jcf7180001958383-augustus-gene-0.0-mRNA-1 | SFR19504 |
| maker-C7_jcf7180001946969-augustus-gene-0.1-mRNA-1 | SFR12215 |
| maker-A5_jcf7180001999199-augustus-gene-0.0-mRNA-1 | SFR21902 |
| maker-ML01_3_jcf7180001333730-augustus-gene-0.0-mRNA-1 | SFR08528 |
| maker-D1_jcf7180002199558-augustus-gene-0.1-mRNA-1 | SFR20418 |
| augustus-KE-4_jcf7180007073185-processed-gene-0.0-mRNA-1 | SFR00377 |
| augustus-B1_jcf7180001505581-processed-gene-0.0-mRNA-1 | SFR19378 |
| maker-C7_jcf7180002095192-augustus-gene-0.0-mRNA-1 | SFR18134 |
| maker-B2_jcf7180001589854-augustus-gene-0.1-mRNA-1 | SFR21474 |
| augustus-A8_jcf7180002032824-processed-gene-0.0-mRNA-1 | SFR13425 |
| augustus-B8_jcf7180001768714-processed-gene-0.0-mRNA-1 | SFR19617 |
| maker-A9_jcf7180001982389-augustus-gene-0.1-mRNA-1 | SFR19621 |
| augustus-ML3_jcf7180002032649-processed-gene-0.2-mRNA-1 | SFR01223 |
| maker-ML2_jcf7180001768628-augustus-gene-0.0-mRNA-1 | SFR14856 |
| maker-C1_jcf7180001803590-augustus-gene-0.0-mRNA-1 | SFR11614 |
| maker-ML01_1_jcf7180001464500-augustus-gene-0.1-mRNA-1 | SFR08926 |
| maker-GX01_jcf7180001592710-augustus-gene-0.1-mRNA-1 | SFR11598 |
| maker-GX01_jcf7180001592710-augustus-gene-0.1-mRNA-1 | SFR10885 |
| maker-A9_jcf7180001954786-augustus-gene-0.2-mRNA-1 | SFR03479 |
| maker-C3_jcf7180001931828-augustus-gene-0.0-mRNA-1 | SFR13001 |
| maker-ML4_jcf7180001656022-augustus-gene-0.0-mRNA-1 | SFR06422 |
| maker-D3_jcf7180001934582-augustus-gene-0.1-mRNA-1 | SFR17437 |
| augustus-A2_jcf7180001812675-processed-gene-0.2-mRNA-1 | SFR16966 |
| maker-A10_jcf7180002283101-augustus-gene-0.1-mRNA-1 | SFR21071 |
| maker-KE-8_jcf7180002736668-augustus-gene-0.1-mRNA-1 | SFR03096 |
| maker-D5_jcf7180001811364-augustus-gene-0.1-mRNA-1 | SFR17396 |
| augustus-A10_jcf7180002270771-processed-gene-0.0-mRNA-1 | SFR20680 |
| augustus-A9_jcf7180001834353-processed-gene-0.0-mRNA-1 | SFR12626 |
| augustus-LJ01_jcf7180001329052-processed-gene-0.0-mRNA-1 | SFR06172 |
| augustus-LJ01_jcf7180001329052-processed-gene-0.0-mRNA-1 | SFR13962 |
| maker-D3_jcf7180002091953-augustus-gene-0.1-mRNA-1 | SFR12004 |
| maker-FJ01_1_jcf7180001284926-augustus-gene-0.0-mRNA-1 | SFR14103 |
| augustus-D5_jcf7180001729070-processed-gene-0.2-mRNA-1 | SFR17253 |
| augustus-A9_jcf7180001918878-processed-gene-0.0-mRNA-1 | SFR02019 |
| augustus-D10_jcf7180002016424-processed-gene-0.0-mRNA-1 | SFR00805 |
| augustus-A5_jcf7180002135874-processed-gene-0.0-mRNA-1 | SFR17945 |
| maker-C1_jcf7180001888578-augustus-gene-0.0-mRNA-1 | SFR16821 |
| maker-A5_jcf7180002160165-augustus-gene-0.1-mRNA-1 | SFR11202 |
| maker-MH03_jcf7180001575088-augustus-gene-0.0-mRNA-1 | SFR05225 |
| maker-MH03_jcf7180001575088-augustus-gene-0.0-mRNA-1 | SFR12493 |
| maker-A8_jcf7180002021803-augustus-gene-0.0-mRNA-1 | SFR05240 |
| maker-D8_jcf7180002034189-augustus-gene-0.1-mRNA-1 | SFR20822 |
| maker-D3_jcf7180002108737-augustus-gene-0.1-mRNA-1 | SFR12498 |
| maker-D3_jcf7180002108737-augustus-gene-0.1-mRNA-1 | SFR06033 |
| augustus-A7_jcf7180001919446-processed-gene-0.0-mRNA-1 | SFR02763 |
| maker-FJ01_1_jcf7180001377011-augustus-gene-0.0-mRNA-1 | SFR18586 |
| augustus-GX01_jcf7180001466047-processed-gene-0.2-mRNA-1 | SFR21792 |
| augustus-MH03_jcf7180001579801-processed-gene-0.0-mRNA-1 | SFR17615 |
| maker-A9_jcf7180001840935-augustus-gene-0.0-mRNA-1 | SFR16810 |
| augustus-A10_jcf7180002310527-processed-gene-0.0-mRNA-1 | SFR00268 |
| maker-D5_jcf7180001710631-augustus-gene-0.1-mRNA-1 | SFR16219 |
| maker-D2_jcf7180002058806-augustus-gene-0.1-mRNA-1 | SFR06523 |
| maker-B5_jcf7180001638303-augustus-gene-0.1-mRNA-1 | SFR02568 |
| maker-B5_jcf7180001638303-augustus-gene-0.1-mRNA-1 | SFR02568 |
| maker-B8_jcf7180001619969-augustus-gene-0.1-mRNA-1 | SFR08956 |
| maker-KE-10_jcf7180003426508-augustus-gene-0.2-mRNA-1 | SFR03355 |
| augustus-C7_jcf7180002106274-processed-gene-0.0-mRNA-1 | SFR19167 |
| maker-LL05_jcf7180001422850-augustus-gene-0.0-mRNA-1 | SFR06877 |
| maker-D10_jcf7180002060145-augustus-gene-0.1-mRNA-1 | SFR04533 |
| augustus-A10_jcf7180002219070-processed-gene-0.0-mRNA-1 | SFR14176 |
| maker-C7_jcf7180002138817-augustus-gene-0.1-mRNA-1 | SFR14700 |
| augustus-D2_jcf7180002018181-processed-gene-0.0-mRNA-1 | SFR07917 |
| augustus-D2_jcf7180002018181-processed-gene-0.0-mRNA-1 | SFR21908 |
| maker-A9_jcf7180001984243-augustus-gene-0.1-mRNA-1 | SFR11322 |
| maker-A7_jcf7180002015996-augustus-gene-0.1-mRNA-1 | SFR14055 |
| maker-A7_jcf7180002015996-augustus-gene-0.1-mRNA-1 | SFR14048 |
| augustus-LL06_jcf7180001258478-processed-gene-0.0-mRNA-1 | SFR19363 |
| augustus-LL06_jcf7180001258478-processed-gene-0.0-mRNA-1 | SFR13369 |
| augustus-A8_jcf7180002016268-processed-gene-0.0-mRNA-1 | SFR14500 |
| maker-ML3_jcf7180001993849-augustus-gene-0.1-mRNA-1 | SFR07007 |
| maker-KE-8_jcf7180002756950-augustus-gene-0.0-mRNA-1 | SFR06877 |
| augustus-LL04_jcf7180001390276-processed-gene-0.0-mRNA-1 | SFR03257 |
| maker-A5_jcf7180001971964-augustus-gene-0.1-mRNA-1 | SFR03479 |
| maker-D4_jcf7180002176043-augustus-gene-0.1-mRNA-1 | SFR16467 |
| maker-D4_jcf7180002176043-augustus-gene-0.1-mRNA-1 | SFR05222 |
| maker-A3_jcf7180001998929-augustus-gene-0.0-mRNA-1 | SFR21552 |
| augustus-LJ01_jcf7180001300900-processed-gene-0.0-mRNA-1 | SFR14500 |
| maker-D8_jcf7180001888129-augustus-gene-0.0-mRNA-1 | SFR10218 |
| maker-FJ01_1_jcf7180001324650-augustus-gene-0.0-mRNA-1 | SFR02991 |
| augustus-D10_jcf7180002022611-processed-gene-0.0-mRNA-1 | SFR01625 |
| augustus-ML3_jcf7180002029922-processed-gene-0.0-mRNA-1 | SFR12444 |
| maker-A2_jcf7180001905191-augustus-gene-0.0-mRNA-1 | SFR06134 |
| augustus-D9_jcf7180001759587-processed-gene-0.0-mRNA-1 | SFR19118 |
| maker-B8_jcf7180001768432-augustus-gene-0.1-mRNA-1 | SFR22145 |
| maker-A6_jcf7180001634289-augustus-gene-0.1-mRNA-1 | SFR03807 |
| maker-KE-9_jcf7180003152487-augustus-gene-0.1-mRNA-1 | SFR08220 |
| maker-C7_jcf7180002135155-augustus-gene-0.1-mRNA-1 | SFR13010 |
| maker-C7_jcf7180002135155-augustus-gene-0.1-mRNA-1 | SFR03391 |
| maker-LL06_jcf7180001259855-augustus-gene-0.1-mRNA-1 | SFR10008 |
| maker-A10_jcf7180002129433-augustus-gene-0.0-mRNA-1 | SFR09978 |
| maker-KE-4_jcf7180006926927-augustus-gene-0.1-mRNA-1 | SFR15618 |
| augustus-A4_jcf7180001888655-processed-gene-0.1-mRNA-1 | SFR05500 |
| augustus-GX02_jcf7180001586437-processed-gene-0.0-mRNA-1 | SFR03257 |
| maker-ML01_3_jcf7180001461221-augustus-gene-0.1-mRNA-1 | SFR03640 |
| maker-B1_jcf7180001593761-augustus-gene-0.0-mRNA-1 | SFR01720 |
| maker-A3_jcf7180002127365-augustus-gene-0.1-mRNA-1 | SFR06134 |
| maker-B4_jcf7180001587286-augustus-gene-0.1-mRNA-1 | SFR08531 |
| maker-MH03_jcf7180001584248-augustus-gene-0.1-mRNA-1 | SFR03355 |
| maker-A7_jcf7180001920913-augustus-gene-0.1-mRNA-1 | SFR06655 |
| maker-KE-8_jcf7180002758570-augustus-gene-0.1-mRNA-1 | SFR02039 |
| maker-B2_jcf7180001495853-augustus-gene-0.1-mRNA-1 | SFR20213 |
| augustus-A4_jcf7180001942232-processed-gene-0.0-mRNA-1 | SFR13010 |
| augustus-A4_jcf7180001942232-processed-gene-0.0-mRNA-1 | SFR03391 |
| augustus-A4_jcf7180001967221-processed-gene-0.2-mRNA-1 | SFR01329 |
| maker-A1_jcf7180001972070-augustus-gene-0.0-mRNA-1 | SFR02515 |
| maker-BS01_jcf7180001646685-augustus-gene-0.0-mRNA-1 | SFR06576 |
| maker-D4_jcf7180002283631-augustus-gene-0.0-mRNA-1 | SFR14444 |
| maker-D2_jcf7180002063705-augustus-gene-0.1-mRNA-1 | SFR16033 |
| augustus-A10_jcf7180002188151-processed-gene-0.0-mRNA-1 | SFR04956 |
| maker-D8_jcf7180001965969-augustus-gene-0.1-mRNA-1 | SFR19621 |
| maker-D1_jcf7180002200665-augustus-gene-0.1-mRNA-1 | SFR14021 |
| maker-ML1_jcf7180002063804-augustus-gene-0.1-mRNA-1 | SFR17501 |
| augustus-C2_jcf7180001932404-processed-gene-0.0-mRNA-1 | SFR13635 |
| maker-FAW2.1_jcf7180001100730-augustus-gene-0.0-mRNA-1 | SFR22145 |
| maker-A10_jcf7180002278344-augustus-gene-0.1-mRNA-1 | SFR15629 |
| augustus-A10_jcf7180002092549-processed-gene-0.0-mRNA-1 | SFR10752 |
| maker-A10_jcf7180002148545-augustus-gene-0.0-mRNA-1 | SFR07806 |
| maker-A4_jcf7180001938946-augustus-gene-0.1-mRNA-1 | SFR04642 |
| augustus-FAW1.1_jcf7180001287268-processed-gene-0.0-mRNA-1 | SFR13478 |
| maker-D4_jcf7180002098181-augustus-gene-0.0-mRNA-1 | SFR03355 |
| augustus-D1_jcf7180002038870-processed-gene-0.0-mRNA-1 | SFR17305 |
| maker-A5_jcf7180002014657-augustus-gene-0.1-mRNA-1 | SFR11713 |
| maker-C10_jcf7180001686660-augustus-gene-0.1-mRNA-1 | SFR06015 |
| augustus-D3_jcf7180002087254-processed-gene-0.0-mRNA-1 | SFR17593 |
| augustus-D1_jcf7180002031553-processed-gene-0.0-mRNA-1 | SFR16951 |
| augustus-D1_jcf7180002031553-processed-gene-0.0-mRNA-1 | SFR16687 |
| augustus-A2_jcf7180001933645-processed-gene-0.0-mRNA-1 | SFR16534 |
| maker-C2_jcf7180001932425-augustus-gene-0.0-mRNA-1 | SFR22145 |
| maker-A9_jcf7180001860314-augustus-gene-0.1-mRNA-1 | SFR17945 |
| augustus-A10_jcf7180002310463-processed-gene-0.0-mRNA-1 | SFR03019 |
| augustus-A10_jcf7180002310463-processed-gene-0.0-mRNA-1 | SFR09934 |
| maker-A2_jcf7180001928785-augustus-gene-0.0-mRNA-1 | SFR02565 |
| augustus-FAW2.1_jcf7180001067736-processed-gene-0.0-mRNA-1 | SFR19287 |
| augustus-FAW2.1_jcf7180001067736-processed-gene-0.0-mRNA-1 | SFR14129 |
| augustus-D4_jcf7180002162692-processed-gene-0.0-mRNA-1 | SFR13123 |
| maker-D6_jcf7180002192136-augustus-gene-0.1-mRNA-1 | SFR19037 |
| maker-A8_jcf7180002127075-augustus-gene-0.0-mRNA-1 | SFR04592 |
| maker-C2_jcf7180001900701-augustus-gene-0.1-mRNA-1 | SFR11834 |
| augustus-KE-5_jcf7180003123182-processed-gene-0.0-mRNA-1 | SFR14457 |
| augustus-A6_jcf7180001635538-processed-gene-0.0-mRNA-1 | SFR04788 |
| maker-A5_jcf7180002162911-augustus-gene-0.0-mRNA-1 | SFR06498 |
| maker-C4_jcf7180001753774-augustus-gene-0.1-mRNA-1 | SFR19621 |
| maker-A7_jcf7180002051578-augustus-gene-0.1-mRNA-1 | SFR00175 |
| maker-KE-7_jcf7180002616565-augustus-gene-0.1-mRNA-1 | SFR20928 |
| maker-A2_jcf7180001798839-augustus-gene-0.0-mRNA-1 | SFR20438 |
| maker-B3_jcf7180000740907-augustus-gene-0.0-mRNA-1 | SFR17085 |
| augustus-FJ01_2_jcf7180001292286-processed-gene-0.0-mRNA-1 | SFR08531 |
| maker-GX04_jcf7180001208245-augustus-gene-0.0-mRNA-1 | SFR05105 |
| augustus-D4_jcf7180002212268-processed-gene-0.0-mRNA-1 | SFR16147 |
| maker-C6_jcf7180001866213-augustus-gene-0.1-mRNA-1 | SFR11834 |
| augustus-D6_jcf7180002078155-processed-gene-0.0-mRNA-1 | SFR03864 |
| augustus-D6_jcf7180002078155-processed-gene-0.0-mRNA-1 | SFR17001 |
| augustus-D6_jcf7180002078155-processed-gene-0.0-mRNA-1 | SFR07114 |
| augustus-GX03_jcf7180001464419-processed-gene-0.0-mRNA-1 | SFR05062 |
| augustus-BS02_jcf7180001416176-processed-gene-0.0-mRNA-1 | SFR13478 |
| maker-KE-7_jcf7180002456630-augustus-gene-0.0-mRNA-1 | SFR10289 |
| augustus-C8_jcf7180001695855-processed-gene-0.0-mRNA-1 | SFR18532 |
| augustus-C8_jcf7180001695855-processed-gene-0.0-mRNA-1 | SFR14027 |
| augustus-FAW1.1_jcf7180001406143-processed-gene-0.0-mRNA-1 | SFR08861 |
| maker-GX03_jcf7180001332607-augustus-gene-0.0-mRNA-1 | SFR11789 |
| maker-GX03_jcf7180001332607-augustus-gene-0.0-mRNA-1 | SFR11786 |
| maker-D4_jcf7180002109768-augustus-gene-0.1-mRNA-1 | SFR15623 |
| maker-D4_jcf7180002109768-augustus-gene-0.1-mRNA-1 | SFR15623 |
| maker-D10_jcf7180002190147-augustus-gene-0.1-mRNA-1 | SFR18681 |
| augustus-LL01_3_jcf7180001482361-processed-gene-0.2-mRNA-1 | SFR19351 |
| augustus-A6_jcf7180001576096-processed-gene-0.0-mRNA-1 | SFR06523 |
| maker-KE-7_jcf7180002606935-augustus-gene-0.1-mRNA-1 | SFR03835 |
| augustus-A4_jcf7180002030973-processed-gene-0.0-mRNA-1 | SFR09922 |
| maker-D6_jcf7180002097429-augustus-gene-0.1-mRNA-1 | SFR17305 |
| maker-B5_jcf7180001687313-augustus-gene-0.0-mRNA-1 | SFR08655 |
| augustus-A10_jcf7180002309838-processed-gene-0.0-mRNA-1 | SFR19378 |
| maker-D9_jcf7180001806666-augustus-gene-0.1-mRNA-1 | SFR05531 |
| maker-ML01_3_jcf7180001322970-augustus-gene-0.0-mRNA-1 | SFR06396 |
| augustus-D8_jcf7180001926499-processed-gene-0.1-mRNA-1 | SFR20654 |
| augustus-D8_jcf7180001926499-processed-gene-0.1-mRNA-1 | SFR18369 |
| augustus-C1_jcf7180001976524-processed-gene-0.0-mRNA-1 | SFR12987 |
| maker-ML3_jcf7180001868322-augustus-gene-0.0-mRNA-1 | SFR20396 |
| augustus-BS01_jcf7180001532714-processed-gene-0.0-mRNA-1 | SFR13994 |
| augustus-C6_jcf7180001922135-processed-gene-0.0-mRNA-1 | SFR04200 |
| augustus-KE-4_jcf7180006681144-processed-gene-0.0-mRNA-1 | SFR11890 |
| maker-C7_jcf7180002139202-augustus-gene-0.0-mRNA-1 | SFR10061 |
| augustus-A2_jcf7180001883283-processed-gene-0.0-mRNA-1 | SFR20788 |
| maker-B8_jcf7180001610460-augustus-gene-0.1-mRNA-1 | SFR22145 |
| maker-C6_jcf7180002027851-augustus-gene-0.0-mRNA-1 | SFR17652 |
| augustus-ML01_1_jcf7180001480592-processed-gene-0.0-mRNA-1 | SFR16904 |
| augustus-ML3_jcf7180002007034-processed-gene-0.2-mRNA-1 | SFR13586 |
| augustus-ML3_jcf7180002007034-processed-gene-0.2-mRNA-1 | SFR12720 |
| maker-A7_jcf7180001930803-augustus-gene-0.0-mRNA-1 | SFR10765 |
| maker-FAW1.1_jcf7180001428725-augustus-gene-0.0-mRNA-1 | SFR08989 |
| maker-B9_jcf7180001545587-augustus-gene-0.0-mRNA-1 | SFR03355 |
| augustus-B6_jcf7180001701182-processed-gene-0.0-mRNA-1 | SFR15656 |
| augustus-B6_jcf7180001701182-processed-gene-0.0-mRNA-1 | SFR09599 |
| augustus-D5_jcf7180001817500-processed-gene-0.0-mRNA-1 | SFR12345 |
| maker-A3_jcf7180002001619-augustus-gene-0.0-mRNA-1 | SFR03479 |
| maker-GX02_jcf7180001451849-augustus-gene-0.1-mRNA-1 | SFR04599 |
| maker-A10_jcf7180002313131-augustus-gene-0.1-mRNA-1 | SFR16837 |
| augustus-A8_jcf7180001997648-processed-gene-0.0-mRNA-1 | SFR11890 |
| maker-ML3_jcf7180001873783-augustus-gene-0.0-mRNA-1 | SFR18512 |
| maker-A10_jcf7180002096992-augustus-gene-0.1-mRNA-1 | SFR15972 |
| augustus-ML3_jcf7180002010845-processed-gene-0.0-mRNA-1 | SFR03096 |
| augustus-C7_jcf7180001917482-processed-gene-0.0-mRNA-1 | SFR06396 |
| maker-A5_jcf7180002014545-augustus-gene-0.0-mRNA-1 | SFR09530 |
| maker-GX02_jcf7180001448410-augustus-gene-0.1-mRNA-1 | SFR00369 |
| augustus-BS02_jcf7180001485231-processed-gene-0.0-mRNA-1 | SFR14353 |
| augustus-A5_jcf7180001983952-processed-gene-0.0-mRNA-1 | SFR18571 |
| maker-BS02_jcf7180001514613-augustus-gene-0.0-mRNA-1 | SFR06200 |
| maker-C10_jcf7180001637146-augustus-gene-0.1-mRNA-1 | SFR06877 |
| augustus-C3_jcf7180001964638-processed-gene-0.0-mRNA-1 | SFR17157 |
| maker-C10_jcf7180001648858-augustus-gene-0.1-mRNA-1 | SFR08655 |
| augustus-C4_jcf7180001915245-processed-gene-0.0-mRNA-1 | SFR09358 |
| maker-LL09_jcf7180001537492-augustus-gene-0.0-mRNA-1 | SFR19706 |
| maker-LL09_jcf7180001537492-augustus-gene-0.0-mRNA-1 | SFR01289 |
| maker-KE-7_jcf7180002495371-augustus-gene-0.0-mRNA-1 | SFR03369 |
| maker-B3_jcf7180000740266-augustus-gene-0.1-mRNA-1 | SFR09169 |
| maker-D9_jcf7180001793823-augustus-gene-0.1-mRNA-1 | SFR02538 |
| maker-LL04_jcf7180001429226-augustus-gene-0.1-mRNA-1 | SFR21200 |
| maker-GX03_jcf7180001375797-augustus-gene-0.1-mRNA-1 | SFR03640 |
| augustus-A1_jcf7180001972702-processed-gene-0.0-mRNA-1 | SFR03870 |
| augustus-A1_jcf7180001972702-processed-gene-0.0-mRNA-1 | SFR18166 |
| maker-C1_jcf7180001790554-augustus-gene-0.0-mRNA-1 | SFR04533 |
| maker-D3_jcf7180001929146-augustus-gene-0.0-mRNA-1 | SFR18166 |
| maker-C1_jcf7180001800231-augustus-gene-0.1-mRNA-1 | SFR05371 |
| maker-A4_jcf7180001942832-augustus-gene-0.0-mRNA-1 | SFR01223 |
| augustus-LL04_jcf7180001486937-processed-gene-0.0-mRNA-1 | SFR08904 |
| maker-D1_jcf7180002154617-augustus-gene-0.1-mRNA-1 | SFR01617 |
| maker-KE-7_jcf7180002494491-augustus-gene-0.2-mRNA-1 | SFR17814 |
| maker-B6_jcf7180001661827-augustus-gene-0.0-mRNA-1 | SFR09604 |
| augustus-KE-5_jcf7180003362983-processed-gene-0.0-mRNA-1 | SFR09012 |
| maker-A3_jcf7180002030113-augustus-gene-0.1-mRNA-1 | SFR21480 |
| augustus-D6_jcf7180002016839-processed-gene-0.0-mRNA-1 | SFR03729 |
| augustus-A9_jcf7180001779958-processed-gene-0.1-mRNA-1 | SFR08527 |
| maker-A4_jcf7180001937351-augustus-gene-0.1-mRNA-1 | SFR02538 |
| maker-A4_jcf7180002036464-augustus-gene-0.0-mRNA-1 | SFR19975 |
| augustus-A7_jcf7180001944040-processed-gene-0.2-mRNA-1 | SFR22122 |
| augustus-B6_jcf7180001673337-processed-gene-0.0-mRNA-1 | SFR12095 |
| maker-BS01_jcf7180001518325-augustus-gene-0.0-mRNA-1 | SFR05531 |
| maker-LL04_jcf7180001426085-augustus-gene-0.1-mRNA-1 | SFR16452 |
| maker-B10_jcf7180001613701-augustus-gene-0.0-mRNA-1 | SFR06877 |
| maker-A7_jcf7180002047624-augustus-gene-0.0-mRNA-1 | SFR11838 |
| augustus-A7_jcf7180001924040-processed-gene-0.0-mRNA-1 | SFR07865 |
| maker-KE-6_jcf7180005200332-augustus-gene-0.0-mRNA-1 | SFR08960 |
| augustus-D5_jcf7180001812552-processed-gene-0.0-mRNA-1 | SFR00684 |
| maker-A10_jcf7180002171012-augustus-gene-0.0-mRNA-1 | SFR03735 |
| maker-A9_jcf7180001771350-augustus-gene-0.1-mRNA-1 | SFR02538 |
| augustus-A3_jcf7180002114580-processed-gene-0.0-mRNA-1 | SFR07531 |
| maker-C6_jcf7180001888524-augustus-gene-0.0-mRNA-1 | SFR11573 |
| maker-LL04_jcf7180001465511-augustus-gene-0.0-mRNA-1 | SFR05105 |
| maker-ML3_jcf7180001831738-augustus-gene-0.1-mRNA-1 | SFR07207 |
| augustus-B5_jcf7180001750870-processed-gene-0.0-mRNA-1 | SFR21483 |
| maker-D5_jcf7180001741926-augustus-gene-0.0-mRNA-1 | SFR18166 |
| augustus-KE-10_jcf7180003467765-processed-gene-0.0-mRNA-1 | SFR14353 |
| maker-B10_jcf7180001493615-augustus-gene-0.1-mRNA-1 | SFR21472 |
| maker-A6_jcf7180001566925-augustus-gene-0.0-mRNA-1 | SFR03479 |
| maker-LL01_3_jcf7180001523543-augustus-gene-0.1-mRNA-1 | SFR00462 |
| maker-D4_jcf7180002332787-augustus-gene-0.0-mRNA-1 | SFR21370 |
| maker-D4_jcf7180002332787-augustus-gene-0.0-mRNA-1 | SFR01541 |
| maker-A10_jcf7180002271168-augustus-gene-0.1-mRNA-1 | SFR19613 |
| maker-D7_jcf7180001844107-augustus-gene-0.0-mRNA-1 | SFR04438 |
| maker-D7_jcf7180001844107-augustus-gene-0.0-mRNA-1 | SFR08482 |
| augustus-LL06_jcf7180001259039-processed-gene-0.0-mRNA-1 | SFR15451 |
| maker-A1_jcf7180001849329-augustus-gene-0.1-mRNA-1 | SFR02763 |
| maker-GX01_jcf7180001528639-augustus-gene-0.0-mRNA-1 | SFR10234 |
| augustus-D1_jcf7180002196776-processed-gene-0.0-mRNA-1 | SFR14556 |
| maker-LL01_2_jcf7180001257274-augustus-gene-0.1-mRNA-1 | SFR12839 |
| maker-B10_jcf7180001621922-augustus-gene-0.1-mRNA-1 | SFR10709 |
| augustus-LL06_jcf7180001364969-processed-gene-0.0-mRNA-1 | SFR02731 |
| augustus-B3_jcf7180000733703-processed-gene-0.0-mRNA-1 | SFR02568 |
| maker-C2_jcf7180001902056-augustus-gene-0.0-mRNA-1 | SFR03373 |
| augustus-A3_jcf7180002055965-processed-gene-0.0-mRNA-1 | SFR02930 |
| maker-A8_jcf7180002139411-augustus-gene-0.0-mRNA-1 | SFR09978 |
| maker-C8_jcf7180001669333-augustus-gene-0.1-mRNA-1 | SFR15293 |
| augustus-D8_jcf7180001887762-processed-gene-0.0-mRNA-1 | SFR09922 |
| maker-D9_jcf7180001912061-augustus-gene-0.1-mRNA-1 | SFR00369 |
| augustus-MH01_jcf7180001474536-processed-gene-0.0-mRNA-1 | SFR03170 |
| augustus-D8_jcf7180001914221-processed-gene-0.0-mRNA-1 | SFR16794 |
| augustus-D8_jcf7180001914221-processed-gene-0.0-mRNA-1 | SFR13268 |
| maker-D10_jcf7180002220020-augustus-gene-0.1-mRNA-1 | SFR19617 |
| maker-FJ01_1_jcf7180001302620-augustus-gene-0.0-mRNA-1 | SFR20928 |
| maker-D3_jcf7180002090311-augustus-gene-0.0-mRNA-1 | SFR05575 |
| maker-D3_jcf7180002090311-augustus-gene-0.0-mRNA-1 | SFR05578 |
| maker-FAW1.1_jcf7180001428432-augustus-gene-0.1-mRNA-1 | SFR05515 |
| maker-JC01_jcf7180001251519-augustus-gene-0.0-mRNA-1 | SFR14489 |
| maker-A1_jcf7180001861714-augustus-gene-0.1-mRNA-1 | SFR07805 |
| maker-FAW5.2_jcf7180001529682-augustus-gene-0.0-mRNA-1 | SFR21900 |
| augustus-FAW2.2_jcf7180001095387-processed-gene-0.1-mRNA-1 | SFR03170 |
| maker-FAW5.2_jcf7180001437090-augustus-gene-0.0-mRNA-1 | SFR21151 |
| maker-FAW5.2_jcf7180001437090-augustus-gene-0.0-mRNA-1 | SFR04126 |
| augustus-C8_jcf7180001657248-processed-gene-0.0-mRNA-1 | SFR08963 |
| maker-A7_jcf7180002058482-augustus-gene-0.1-mRNA-1 | SFR13965 |
| maker-A3_jcf7180001965919-augustus-gene-0.1-mRNA-1 | SFR05515 |
| maker-KE-7_jcf7180002479199-augustus-gene-0.1-mRNA-1 | SFR21630 |
| augustus-KE-8_jcf7180002753718-processed-gene-0.0-mRNA-1 | SFR19399 |
| augustus-C7_jcf7180001965443-processed-gene-0.0-mRNA-1 | SFR19351 |
| augustus-C7_jcf7180001991040-processed-gene-0.2-mRNA-1 | SFR03667 |
| maker-BS01_jcf7180001675359-augustus-gene-0.0-mRNA-1 | SFR18388 |
| maker-A3_jcf7180001967637-augustus-gene-0.0-mRNA-1 | SFR15744 |
| augustus-D7_jcf7180001939521-processed-gene-0.2-mRNA-1 | SFR12365 |
| augustus-A5_jcf7180002000054-processed-gene-0.0-mRNA-1 | SFR19504 |
| maker-KE-9_jcf7180003282786-augustus-gene-0.1-mRNA-1 | SFR16841 |
| maker-FAW5.2_jcf7180001391441-augustus-gene-0.1-mRNA-1 | SFR02920 |
| maker-B9_jcf7180001436942-augustus-gene-0.0-mRNA-1 | SFR18512 |
| maker-D3_jcf7180001967883-augustus-gene-0.0-mRNA-1 | SFR20921 |
| augustus-A9_jcf7180001848837-processed-gene-0.0-mRNA-1 | SFR13510 |
| maker-A2_jcf7180001813191-augustus-gene-0.1-mRNA-1 | SFR00637 |
| maker-D6_jcf7180002236283-augustus-gene-0.1-mRNA-1 | SFR13145 |
| augustus-D4_jcf7180002155173-processed-gene-0.0-mRNA-1 | SFR06004 |
| maker-D10_jcf7180002047703-augustus-gene-0.1-mRNA-1 | SFR00817 |
| maker-D7_jcf7180002043023-augustus-gene-0.0-mRNA-1 | SFR19351 |
| maker-C8_jcf7180001712986-augustus-gene-0.1-mRNA-1 | SFR20867 |
| augustus-C2_jcf7180001757103-processed-gene-0.2-mRNA-1 | SFR16842 |
| maker-B8_jcf7180001736482-augustus-gene-0.0-mRNA-1 | SFR20654 |
| maker-B8_jcf7180001736482-augustus-gene-0.0-mRNA-1 | SFR18369 |
| maker-D1_jcf7180002199299-augustus-gene-0.0-mRNA-1 | SFR10224 |
| maker-D8_jcf7180001984373-augustus-gene-0.1-mRNA-1 | SFR07639 |
| maker-LL06_jcf7180001297791-augustus-gene-0.1-mRNA-1 | SFR02920 |
| maker-C1_jcf7180001835021-augustus-gene-0.0-mRNA-1 | SFR12998 |
| maker-KE-3_jcf7180003561944-augustus-gene-0.0-mRNA-1 | SFR11202 |
| maker-D4_jcf7180002108752-augustus-gene-0.0-mRNA-1 | SFR01549 |
| augustus-A2_jcf7180001772179-processed-gene-0.0-mRNA-1 | SFR07280 |
| augustus-JC01_jcf7180001382577-processed-gene-0.0-mRNA-1 | SFR13807 |
| augustus-FAW2.2_jcf7180001106212-processed-gene-0.2-mRNA-1 | SFR08226 |
| maker-KE-4_jcf7180006664474-augustus-gene-0.0-mRNA-1 | SFR17195 |
| augustus-B9_jcf7180001468367-processed-gene-0.0-mRNA-1 | SFR17999 |
| maker-D4_jcf7180002331569-augustus-gene-0.0-mRNA-1 | SFR22145 |
| augustus-KE-7_jcf7180002694542-processed-gene-0.0-mRNA-1 | SFR02124 |
| maker-D1_jcf7180002165516-augustus-gene-0.0-mRNA-1 | SFR21666 |
| maker-C1_jcf7180001849785-augustus-gene-0.1-mRNA-1 | SFR01179 |
| maker-C1_jcf7180001849785-augustus-gene-0.1-mRNA-1 | SFR11624 |
| augustus-A10_jcf7180002273379-processed-gene-0.0-mRNA-1 | SFR06877 |
| augustus-A4_jcf7180002094153-processed-gene-0.0-mRNA-1 | SFR21991 |
| maker-ML3_jcf7180001842881-augustus-gene-0.0-mRNA-1 | SFR02127 |
| maker-B2_jcf7180001501967-augustus-gene-0.0-mRNA-1 | SFR20701 |
| maker-KE-9_jcf7180003485834-augustus-gene-0.1-mRNA-1 | SFR13213 |
| maker-A1_jcf7180001986733-augustus-gene-0.1-mRNA-1 | SFR16506 |
| augustus-MH03_jcf7180001612118-processed-gene-0.0-mRNA-1 | SFR10250 |
| maker-B10_jcf7180001613194-augustus-gene-0.0-mRNA-1 | SFR10791 |
| augustus-KE-7_jcf7180002527185-processed-gene-0.0-mRNA-1 | SFR04676 |
| maker-D3_jcf7180001928754-augustus-gene-0.1-mRNA-1 | SFR07874 |
| maker-D3_jcf7180001985281-augustus-gene-0.1-mRNA-1 | SFR07338 |
| augustus-D4_jcf7180002102834-processed-gene-0.0-mRNA-1 | SFR22189 |
| maker-D5_jcf7180001796395-augustus-gene-0.0-mRNA-1 | SFR03998 |
| maker-D1_jcf7180002143909-augustus-gene-0.0-mRNA-1 | SFR19504 |
| maker-A2_jcf7180001806808-augustus-gene-0.0-mRNA-1 | SFR02538 |
| augustus-ML3_jcf7180001871981-processed-gene-0.2-mRNA-1 | SFR14211 |
| augustus-D2_jcf7180001997646-processed-gene-0.0-mRNA-1 | SFR18532 |
| augustus-D2_jcf7180001997646-processed-gene-0.0-mRNA-1 | SFR14027 |
| augustus-D6_jcf7180002102058-processed-gene-0.0-mRNA-1 | SFR03928 |
| augustus-D6_jcf7180002102058-processed-gene-0.0-mRNA-1 | SFR18059 |
| augustus-BS01_jcf7180001541459-processed-gene-0.0-mRNA-1 | SFR04694 |
| maker-KE-9_jcf7180003343840-augustus-gene-0.1-mRNA-1 | SFR17301 |
| augustus-KE-9_jcf7180003458057-processed-gene-0.0-mRNA-1 | SFR22145 |
| maker-GX02_jcf7180001549893-augustus-gene-0.0-mRNA-1 | SFR14701 |
| maker-A9_jcf7180001841829-augustus-gene-0.0-mRNA-1 | SFR12448 |
| maker-A10_jcf7180002087693-augustus-gene-0.0-mRNA-1 | SFR02538 |
| maker-FAW2.1_jcf7180001036230-augustus-gene-0.0-mRNA-1 | SFR10212 |
| maker-B8_jcf7180001734339-augustus-gene-0.0-mRNA-1 | SFR03355 |
| augustus-A10_jcf7180002272805-processed-gene-0.2-mRNA-1 | SFR19422 |
| maker-C3_jcf7180001908510-augustus-gene-0.0-mRNA-1 | SFR14875 |
| maker-C3_jcf7180001908510-augustus-gene-0.0-mRNA-1 | SFR10389 |
| maker-A7_jcf7180002071240-augustus-gene-0.0-mRNA-1 | SFR19045 |
| maker-B8_jcf7180001738092-augustus-gene-0.1-mRNA-1 | SFR19617 |
| maker-C1_jcf7180001952549-augustus-gene-0.1-mRNA-1 | SFR04991 |
| maker-B10_jcf7180001550861-augustus-gene-0.0-mRNA-1 | SFR17596 |
| maker-A7_jcf7180001874950-augustus-gene-0.0-mRNA-1 | SFR00393 |
| maker-A8_jcf7180002125121-augustus-gene-0.1-mRNA-1 | SFR10765 |
| maker-FJ01_2_jcf7180001328038-augustus-gene-0.1-mRNA-1 | SFR08645 |
| maker-C10_jcf7180001605255-augustus-gene-0.0-mRNA-1 | SFR11789 |
| maker-C10_jcf7180001605255-augustus-gene-0.0-mRNA-1 | SFR11786 |
| maker-GX04_jcf7180001219031-augustus-gene-0.0-mRNA-1 | SFR22145 |
| maker-LL04_jcf7180001394190-augustus-gene-0.0-mRNA-1 | SFR08989 |
| maker-KE-3_jcf7180003485188-augustus-gene-0.0-mRNA-1 | SFR08904 |
| augustus-C7_jcf7180002095984-processed-gene-0.0-mRNA-1 | SFR11165 |
