# Supplementary figures and images for: Is the extreme within-population genome size variation real in Spodoptera frugiperda?
Source: PLoS One. 2025 Sep 30;20(9):e0332711. doi: 10.1371/journal.pone.0332711 (PMC12483198; doi:10.1371/journal.pone.0332711)

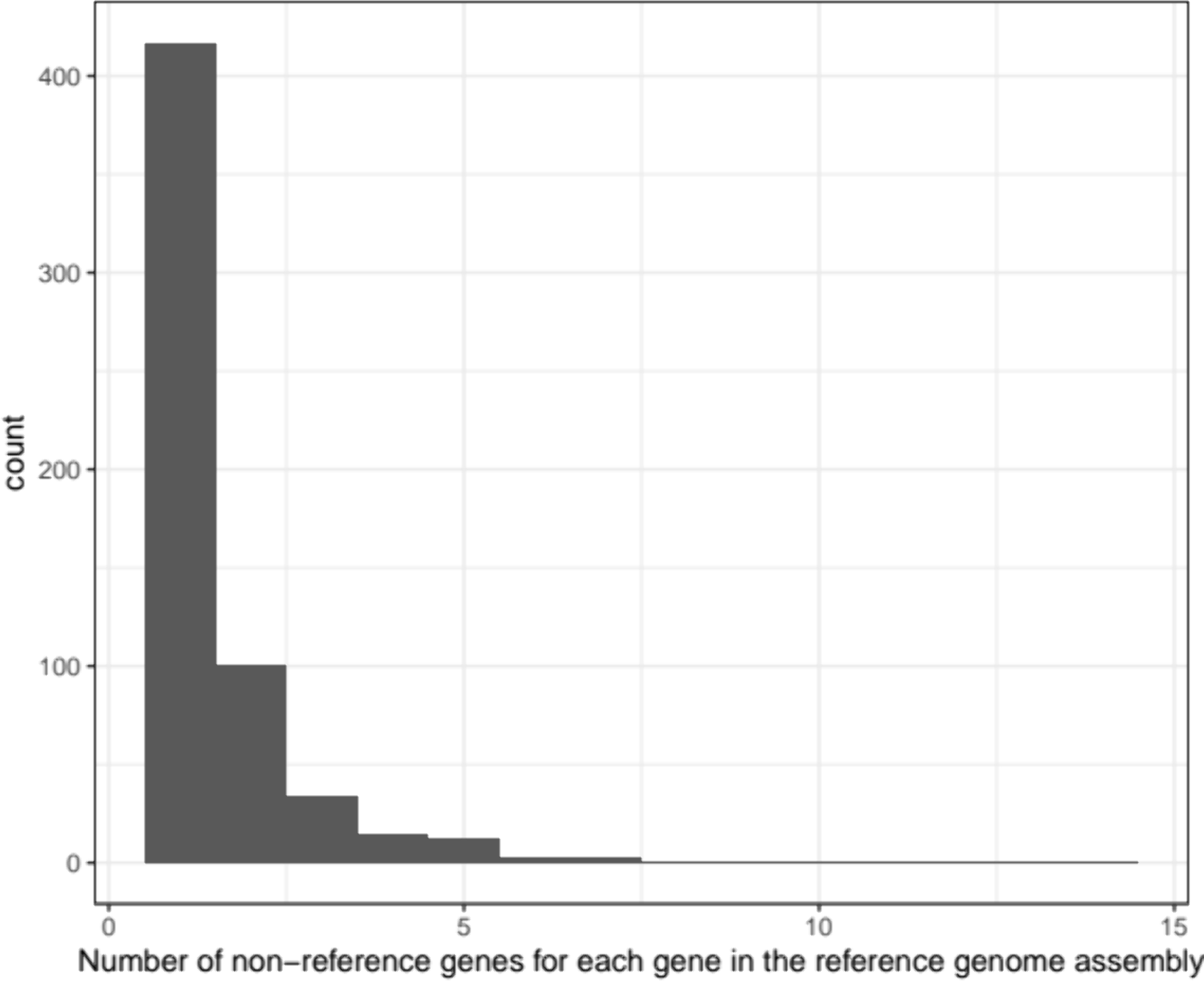

Supplement: S1 Fig — (TIF) [file pone.0332711.s004.tif]

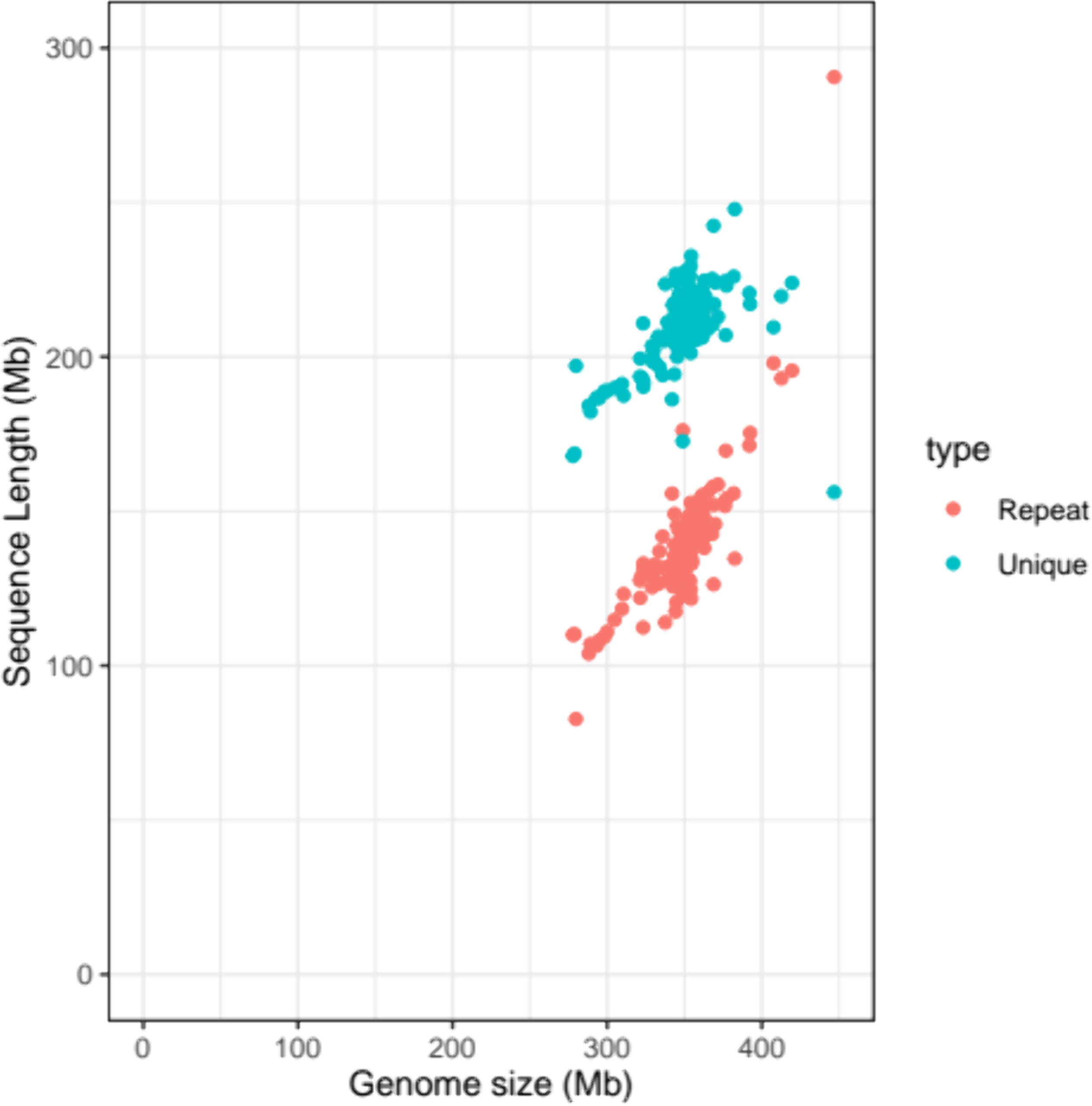

Supplement: S2 Fig — (TIF) [file pone.0332711.s005.tif]
