# Supplementary material for: Is the extreme within-population genome size variation real in Spodoptera frugiperda?
Source: PLoS One. 2025 Sep 30;20(9):e0332711. doi: 10.1371/journal.pone.0332711 (PMC12483198; doi:10.1371/journal.pone.0332711)
Supplement: Attachment — Submitted filename: Rebuttal_auresp_2.pdf [file pone.0332711.s007.pdf]

Reviewer #1: Thank you for your careful revisions to address all the comments raised. After reviewing your responses and the corresponding modifications in the manuscript, I am satisfied with the improvements made.
Regarding the spelling and format issues, the unification of terms, clarification of abbreviations (e.g., "mya"), correction of table number references, and standardization of software name formatting (e.g., specifying "Genome Complexity Estimator (GCE)" for the first mention) have been properly handled, enhancing the consistency and readability of the text.
For the concerns about assembly strategies and genome size, while the exact cause of genome size inflation in Gui et al.'s work remains challenging to pinpoint due to limited data availability, the addition of assembly statistics in Table 1 and relevant text (L211–212) provides valuable context for readers to evaluate assembly quality, which is a reasonable approach within the scope of this study.
The clarification of sample sources (Zhang et al.'s samples mostly from maize fields, with two exceptions from sugarcane) in L213–216 addresses the representativeness issue of geographical population samples, providing necessary context for interpreting host-related analyses.
The inclusion of detailed methodological information and confidence intervals for divergence time estimation (L183–187) strengthens the robustness of the evolutionary stability analysis.
The supplementation of specific details from the original literature for flow cytometry data (L63–65) and the specification of software versions (e.g., Genomescope v1.0 in L224–225) improve the transparency and reproducibility of the research methods.
The addition of specific examples (100% protein sequence identity cases in Table S3, L128; metazoan BUSCO genes present in both reference and non-reference assemblies in L123–126 and L129) effectively supports the verification of non-reference sequence contamination, enhancing the persuasiveness of the related conclusions.
Overall, the revisions have adequately addressed all the raised concerns, and the manuscript now meets the requirements for publication. I recommend accepting the manuscript in its current form.

R1: Thank you for recommending the publication of this manuscript without further revision.

Reviewer #2: The authors have addressed convincingly my initial comments and I believe that the manuscript is now suitable for publication
R2: Thank you for recommending the publication of this manuscript.
